# Age-, body surface area-, and sex-specific reference values for cardiovascular magnetic resonance imaging derived ventricular and atrial size and function for Chinese healthy children

**DOI:** 10.1016/j.jocmr.2025.101885

**Published:** 2025-03-21

**Authors:** Ke Xu, Wei Bai, Zhi Yang, Rong Xu, Lin-jun Xie, Ling-yi Wen, Chuan Fu, Jie-qian Zheng, Xin-mao Ma, Hang Fu, Zhong-qin Zhou, Cheng-cheng Zhu, Xiao-yue Zhou, Hua-yan Xu, Ying-kun Guo

**Affiliations:** a Department of Radiology, West China Second University Hospital, Sichuan University, Key Laboratory of Birth Defects and Related Diseases of Women and Children (Sichuan University), Ministry of Education, Chengdu, China; b Department of Radiology, Chengdu Fifth People’s Hospital, Chengdu, China; c Department of Radiology, University of Washington, Seattle, Washington, USA; d Siemens Healthineers Digital Technology (Shanghai) Co., Ltd., Shanghai, China

**Keywords:** Cardiovascular magnetic resonance, Children, Reference value, Sex, Chamber size

## Abstract

**Background:**

Cardiovascular magnetic resonance (CMR) is crucial for the diagnosis and prognosis of heart disease. However, normal reference values for CMR-derived morphology parameters have not been established for Chinese children. We sought to establish reference values for ventricular and atrial size and function parameters using CMR in healthy Chinese children across a broad age range.

**Methods:**

3T CMR scans were performed in 191 healthy children, aged 4–18 years. We used balanced steady-state free precession sequence for analyzing chamber size and function. Reference percentile curves and tables were generated using the generalized additive model for location scale and shape. A meta-analysis was conducted to compare our results with those of relevant previously published studies.

**Results:**

Boys generally had greater ventricular volumes and masses after normalization for body surface area (BSA) compared with girls. However, in the youngest age group (4–8 years), differences in volumes or masses between sexes were not found. Additionally, differences were not observed in left ventricular and right ventricular ejection fractions between sexes upon stratifying subjects based on age groups. However, after normalizing for BSA, only the maximal right atrial volume remained significantly greater in boys than that in girls. Age-specific and BSA-specific reference curves revealed non-linear relationships between age/BSA and cardiac parameters. Asian children exhibited significantly smaller chamber sizes compared to those seen in Caucasian children.

**Conclusion:**

We report normative CMR ventricular and atrial volume and function in Chinese children based on BSA, age, and sex, which can serve as a reference for future studies and clinical practice.

## Graphical abstract

**Figure:**
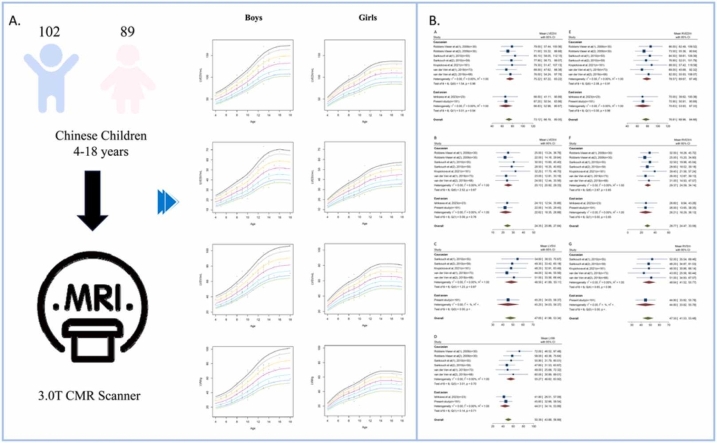

## 1. Introduction

Accurate quantification of cardiac chamber size and function is fundamental for risk stratification, diagnosis, and treatment decisions across a spectrum of cardiovascular conditions [1]. Cardiovascular magnetic resonance (CMR) has been considered the reference standard for the assessment of cardiac structure and function in both adult and pediatric populations due to its high accuracy and reproducibility [2], [3]. Nevertheless, the efficacy of CMR depends on the establishment of reference ranges to distinguish normal cardiac parameters from those in pathological conditions [4].

A recent study including 9088 healthy individuals from the Healthy Hearts Consortium, spanning the full adult age spectrum, established age-, sex-, and ethnicity-specific CMR reference ranges for atrial and ventricular metrics in adults [5]. However, the absence of comprehensive CMR reference ranges for cardiac size and function derived from large, healthy pediatric cohorts remains a major shortcoming for clinical care. Current CMR normal reference ranges for cardiac size and function in children are derived from small, ethnically homogeneous pediatric cohorts which primarily consisted Caucasian children from Europe and the USA [6], [7], [8], [9], [10], [11], [12], [13], [14], [15], [16], [17], limiting their applicability to diverse global populations. Studies comparing cardiac chamber parameters in children of different ethnicities have not been published. We hypothesized that ethnic differences in cardiac parameters are consistent across both children and adults [18], [19], [20].

The objectives of this study were (1) to establish reference values for left ventricular (LV), right ventricular (RV), left atrial (LA), and right atrial (RA) structure and function in healthy Chinese children stratified by body surface area (BSA), age, and sex; and (2) to compare our normal CMR reference values with reference values derived from Caucasian children in previous studies by conducting a meta-analysis.

## 2. Methods

### 2.1 Study population

A total of 201 healthy children were prospectively recruited through a public recruitment poster or by distributing flyers from October 2019 to March 2023. Prior to inclusion and CMR, parents underwent a brief questionnaire regarding their child’s clinical history and family history to ensure that children with significant systemic diseases or genetic conditions were excluded. Height, weight, and resting blood pressure were measured on the day of CMR scanning. Physical examination and electrocardiogram were conducted and medical information, including the absence of pediatrician concerns and emergency department visits since scheduling, was verified to ensure that both inclusion and exclusion criteria were met. The inclusion criteria were children aged between 0 and 18 years with no evidence or history of cardiovascular disease. The exclusion criteria comprised the presence of any disease affecting the cardiovascular system, acute infections, arterial hypertension, arrhythmias, diabetes mellitus, and a family history of cardiac disease. Subjects with contraindications for CMR or those participating in any competitive sporting activities were also excluded from the present study.

The Ethics Committee of West China Second University Hospital of Sichuan University approved the study protocol (No.2022–105). Written informed consent was signed by all parents or legal guardians.

### 2.2 CMR protocol

All CMR examinations were performed without sedation or anesthesia using a 3T whole-body scanner (MAGNETOM Skyra; Siemens Healthineers, Erlangen, Germany) with a dedicated 18-element body coil array. Images were obtained with breath-holding at end-expiration whenever possible. For young children unable to follow breath-holding instructions during the scan, images were acquired while breathing freely. To ensure the successful completion of MR scans in young children without sedation, several child-friendly techniques and specialized pediatric protocols were employed. These methods included preparatory sessions with both parents and children to familiarize them with the scanning process, the use of distraction techniques, such as videos or music, and the presence of a parent or guardian during the scan, when possible. Cine sequences were acquired with retrospective electrocardiographic gating, using a balanced steady-state-free precession pulse (bSSFP) sequence: echo time = 1.23 ms, repetition time = 3.42 ms, flip angle = 60°, slice thickness = 8 mm, slice gap = 2 mm, matrix = 126 × 224 pixels, field of view = 250 × 300 mm^2^, temporal resolution = 44.4 ms, and signal averages = 1 (breath-holding) or 3 (free-breathing). In addition to 8–13 contiguous parallel short-axis slices covering the entire ventricles, 2-chamber (2Ch) and 4-chamber (4Ch) long-axis acquisitions were also acquired.

### 2.3 CMR image analysis

CMR image analysis was performed by two expert radiologists (K.X. and W.B., with 5 and 7 years of CMR experience, respectively) using the dedicated post-processing software Cvi42 (Circle Cardiovascular Imaging, Calgary, Alberta, Canada). Subjects whose scans exhibited inadequate image quality or artifacts that could lead to unreliable measurements were excluded from the analysis.

The cine short-axis stack was used to perform quantitative LV/RV functional and volumetric evaluations applying two different contouring techniques, namely smooth segmentation and anatomic segmentation. Using contouring method 1 (Smooth segmentation), trabeculations and papillary muscles were included in the ventricular blood volume and excluded from the myocardial mass. The LV endocardial and epicardial borders at end-diastole and end-systole were traced using semi-automatic contour detection, with manual correction if necessary. RV endocardial contours were drawn manually. Using contouring method 2 (Anatomic segmentation), trabeculations and papillary muscles were excluded from the ventricular blood volume and included in the myocardial mass. LV and RV endocardial borders were traced using the built-in threshold tool, with manual correction if necessary. LV epicardial borders were also drawn manually.

**Fig. 1:**
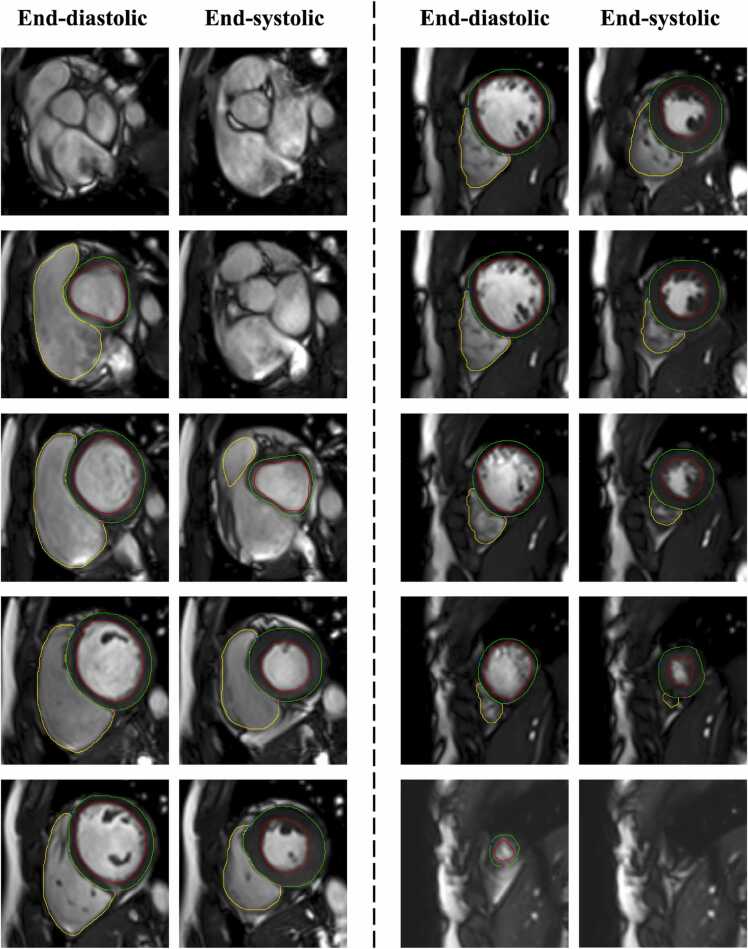
Contouring method with inclusion of trabeculations and papillary muscles in the ventricular volume at end-systole and end-diastole

For quantitative LV volume and function, the most basal slice, surrounded by at least 50% of the myocardium at the end-systolic phase, was considered the base, and the most apical slice with a visible cavity at the end-diastolic phase was considered the apex. The basal slice of the RV was corroborated with the long-axis views, and the RV outflow tract was included in the RV volume. Slices were considered to be in the RV, rather than the RA or pulmonary artery, if myocardium with trabeculations was visible. In addition, the matching long-axis planes were used for additional information on slice localization. Examples of smooth segmentation and anatomic segmentation are illustrated in Fig. 1 and Supplemental Fig. S1, respectively.

We then determined the biventricular end-diastolic volume (EDV), end-systolic volume (ESV), stroke volume (SV), and ejection fraction (EF) data for all subjects. The LV mass (LVM) was obtained from the end-diastolic frames. LVM was derived by the summation of disks method by multiplying myocardial muscle volume by its specific gravity (1.05 g/cm^3^). Parameters of biventricular structure and function were indexed to BSA. BSA was calculated using the Du Bois & Du Bois formula [21]: BSA (m^2^) = 0.007184 × height (cm)^0.725^ × weight (kg)^0.425^.

LA and RA endocardial contours were drawn on 4Ch-views at the times in the cardiac cycle when the atria reached the maximum and minimum size. Maximal and minimal LA volumes (LAV_max_ and LAV_min_) were measured using the biplane area-length method with 4Ch and 2Ch views. Maximal and minimal RA volumes (RAV_max_ and RAV_min_) were calculated using the monoplane area-length method, because the RA could be assessed only in the 4Ch view. An example of measurement is shown in Supplemental Fig. S2.

### 2.4 Intra- and inter-observer variability

Intra- and inter-observer variability were assessed for 20 randomly selected subjects. For the intra-observer analysis, the studies were reanalyzed by the same reviewer with a time interval of at least 2 months. The second radiologist, who was blinded to the results of the first observer, analyzed the same randomly selected patients to assess inter-observer variability.

### 2.5 Meta-analysis

A literature search was performed in PubMed and Embase to identify publications on CMR reference values in children, as described by Kawel‑Boehm [4]. Study selection for the meta-analysis involved a process of title screening, abstract review, and full-text review. The general criteria used for data inclusion in this meta-analysis were as follows:

(1) We selected studies that defined a normal reference range for at least one of our CMR parameters in healthy children (≤18 years-old) with sample sizes ≥50, reported in the English language.
(2) Quantification was required to have been performed using bSSFP sequences at 1.5T or 3T.
(3) Studies selected for quantitative analysis were required to have reported both mean and standard deviation values indexed to BSA.

### 2.6 Statistical analysis

Statistical analysis was performed with IBM SPSS Statistics software v.26.0 (IBM SPSS Statistics, IBM Corporation, Armonk, New York), R software v.4.2.2 (R Foundation for Statistical Computing, Vienna, Austria), and the Stata software v.16.0 (STATA, Stata Corporation, College Station, Texas). Continuous variables were expressed as mean ± standard deviation (SD) or median and inter-quartile ranges. Categorical variables were reported as absolute numbers (percentages). Normality distribution was tested using the Kolmogorov–Smirnov test. In addition, means between any two groups were compared using an independent Student’s *t*-test or the Mann–Whitney U test. Reference percentile curves and tables were generated using the generalized additive model for location scale and shape (GAMLSS) package in R, which is an extended version of the lambda-mu-sigma method by Cole [22]. We used cubic spline as the smoothing function to establish the percentile curves of morphology parameters. Reference values were defined by sex- and age- specific centiles, and sex- and BSA-specific centiles. Within the GAMLSS framework, we assessed the Box-Cox power exponential, Box-Cox Cole and Green, and Box-Cox t distribution families. The optimal model was selected using the principle of minimum Akaike information criterion, and the fit of the percentile curve was assessed with residual vs. fitted plots, density plots of residuals, worm plots, and standard q-q plots. The final GAMLSS model parameters were used to generate centile tables and curves for boys and girls separately. Results from multiple studies reporting normal values for the same CMR parameters were pooled using a random effects meta-analysis model as implemented by the metan command of Stata [23]. This produced a weighted, pooled estimate of the population mean of the CMR parameters in the combined studies. Upper and lower limits were calculated as ± 1.96SDp, where SDp is the pooled standard deviation calculated from the standard deviations reported in each study. Between-study heterogeneity was assessed using the inconsistency index (I²). For subgroup analysis, the mean difference (MD) and related P-values are presented. Intra- and inter-observer variability for continuous CMR variables was assessed using the Bland–Altman method (15). Furthermore, the coefficient of variation (COV) was reported. A p-value of <0.05 was considered statistically significant.

## 3. Results

### 3.1 Demographics

**Fig. 2:**
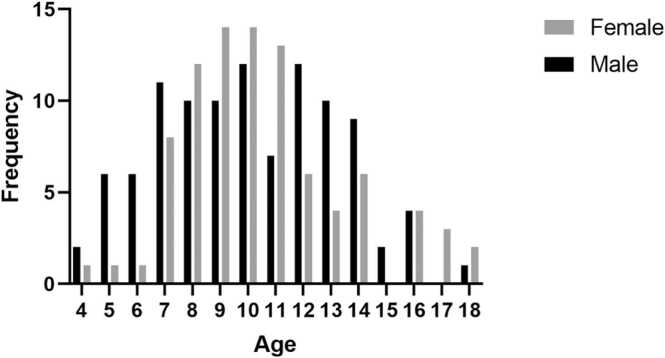
Sex and age distribution

We analyzed cardiac morphology and function in 191 children enrolled in the study. All subjects were Chinese. Subject characteristics are shown in Table 1. The median subject age was 10.0 years (range 4.0–18.0 years). Age distribution between sexes was similar, and the number of children under 6 years of age was limited, as shown in the demographic pyramid graph (Fig. 2). When data were not stratified based on age, significant differences in body size, height, or weight between the sexes were not found. Three age categories were defined to represent young (4–8 years), middle-aged (9–13 years), and older (14–18 years) children.

**Table 1:** Demographic characteristics.

	Total		4–8 years		9–13 years		14–18 years	
	Boys (n = 102)	Girls (n = 89)	Boys (n = 35)	Girls (n = 23)	Boys (n = 51)	Girls (n = 51)	Boys (n = 16)	Girls (n=15)
Age(years)	10.0[7.8–13.0]	10.0[8.0–12.0]	7.0[6.0–8.0]	8.0[7.0–8.0]	11.0[10.0–12.0]	10.0[9.0–11.0]	14.0[14.0–16.0]	16.0[14.0–17.0]
Weight(kg)	34.8[25.8–50.0]	37.0[29.0–45.0]	23.0[19.0–30.0]	28.0[23.0–34.0]	38.0[33.5–51.3]	38.0[31.5–42.0]	55.5[50.0–60.8]	48.5[48.0–59.1]
Height(cm)	143.0[126.0–161.0]	143.0[133.3–155.0]	122.0[114.2–130.0]	130.0[124.0–136.0]	152.0[140.0–161.0]	144.0[139.0–155.0]	169.0[161.1–172.8]	160.0[155.0–165.0]
BSA(m^2^)	1.2[1.0–1.5]	1.2[1.0–1.4]	0.9[0.8–1.0]	1.0[0.9–1.1]	1.3[1.1–1.5]	1.3[1.1–1.4]	1.6[1.5–1.7]	1.5[1.4–1.7]
BMI(kg/ m^2^)	18.0[15.3–19.4]	17.1[15.6–19.5]	15.4[14.5–18.0]	16.4[14.5–17.3]	17.2[16.5–19.3]	16.9[15.6–19.3]	20.4[17.1–21.8]	20.2[19.1–20.8]
Heart rate(bpm)	80.6[71.5–93.2]	81.9[70.6–92.0]	86.1[81.1–102.1]	89.4[77.4–98.3]	75.3[70.4–85.5]	78.5[71.4–86.3]	70.7[64.9–85.9]	70.1[64.0–88.6]

The data are presented as median (inter-quartile range). *BSA* body surface area, *BMI* body mass index

### 3.2 Reference values of ventricular size and function parameters

Of the 191 children, 15 were unable to follow breath-hold instructions; hence, scanning was conducted with subjects breathing freely. Ventricular size and function parameters with inclusion and exclusion of trabeculations and papillary muscles in ventricular volumes are described in Table 2, Table 3, respectively. Irrespective of the underlying method, we observed higher values for ventricular volumes and masses in boys compared to those in girls. In the youngest age group (4–8 years), differences in volumes or masses were not observed between sexes. In the middle-aged and oldest age groups, all volumes and masses indexed to BSA were higher in boys, except for LV end-systolic volume index (LVESVi). However, differences in left ventricular ejection fraction (LVEF) and right ventricular ejection fraction (RVEF) were not observed between sexes and even upon stratification based on age groups. Biventricular EDV, ESV, and SV, with the inclusion of trabeculations and papillary muscles in ventricular volumes, were significantly higher compared to those with the exclusion of trabeculations and papillary muscles. In contrast, LVM, LVEF, and RVEF, with the exclusion of trabeculations and papillary muscles in ventricular volume, were significantly higher compared to values obtained with the inclusion of trabeculations and papillary muscles (Supplemental Table S1).

**Table 2:** Morphology and function parameters of the LV and RV with inclusion of trabeculations and papillary muscles in ventricular volume.

	Total		4–8 years		9–13 years		14–18 years	
	Boys (n = 102)	Girls (n = 89)	Boys (n = 35)	Girls (n = 23)	Boys (n = 51)	Girls (n = 51)	Boys (n = 16)	Girls (n = 15)
LVEDV(mL)	93.7 ± 30.2	88.4 ± 19.9	66.2 ± 14.6	72.0 ± 15.0	101.8 ± 24.1*	89.5 ± 17.2	128.3 ± 21.8*	110.0 ± 12.0
LVESV(mL)	36.3 ± 13.1	34.5 ± 8.6	25.3 ± 6.0	27.3 ± 6.2	39.4 ± 11.3*	34.9 ± 7.2	50.2 ± 11.0	44.0 ± 6.1
LVSV(mL)	57.5 ± 17.6	53.9 ± 11.8	40.9 ± 9.0	44.8 ± 9.3	62.4 ± 13.3*	54.6 ± 10.5	78.1 ± 11.6*	66.0 ± 7.2
LVEF(%)	61.6 ± 2.8	61.2 ± 2.6	61.9 ± 2.6	62.2 ± 2.8	61.6 ± 2.8	61.0 ± 2.3	61.2 ± 2.9	60.1 ± 2.6
LVM(g)	49.6 ± 18.1	44.3 ± 10.7	33.4 ± 8.1	34.7 ± 6.5	54.6 ±15.1*	45.2 ± 9.3	69.4 ± 14.2*	56.3 ± 6.2
RVEDV(mL)	99.8 ± 33.9	91.4 ± 21.4	67.7 ± 16.3	72.8 ± 15.8	109.3 ±24.9*	92.6 ± 17.5	140.6 ± 25.5*	115.9 ± 13.4
RVESV(mL)	42.6 ± 17.5	37.9 ± 10.4	26.9 ± 7.9	28.4 ± 7.4	47.3 ± 13.4*	38.6 ± 8.1	62.3 ± 16.0*	50.1 ± 6.9
RVSV(mL)	57.2 ± 17.4	53.5 ± 12.1	40.8 ± 9.2	44.4 ± 9.4	62.0 ± 13.4*	54.0 ± 10.9	77.3 ± 11.8*	65.7 ± 7.4
RVEF(%)	58.1 ± 4.8	58.8 ± 4.1	60.5 ± 4.1	61.2 ± 4.3	57.2 ± 4.8	58.3 ± 4.0	55.8 ± 4.7	56.8 ± 2.4
LVEDVi(mL/m^2^)	76.1 ± 10.0*	72.3 ± 7.9	72.8 ± 9.9	72.2 ± 8.9	77.2 ± 9.6*	72.3 ± 7.8	79.7 ± 9.8*	72.3 ± 6.8
LVESVi(mL/m^2^)	29.3 ± 4.8	28.1 ± 3.9	27.7 ± 4.1	27.4 ± 4.3	29.7 ± 5.0	28.2 ± 3.7	31.1 ± 5.0	28.9 ± 3.9
LVSVi(mL/m^2^)	46.9 ± 6.0*	44.2 ± 4.8	45.1 ± 6.6	44.9 ± 5.3	47.4 ± 5.5*	44.1 ± 4.8	48.6 ± 5.6*	43.3 ± 3.8
LVMi(g/m^2^)	40.0 ± 6.5*	36.1 ± 4.2	36.7 ± 5.2	34.9 ± 4.0	41.1 ± 6.3*	36.4 ± 4.5	43.1 ± 7.2*	37.0 ± 3.1
RVEDVi(mL/m^2^)	80.7 ± 11.9*	74.5 ± 8.3	74.5 ± 11.9	72.9 ± 9.3	83.0 ± 10.2*	74.9 ± 8.1	86.6 ± 11.7*	76.1 ± 7.1
RVESVi(mL/m^2^)	34.1 ± 7.7*	30.8 ± 5.1	29.5 ± 6.4	28.4 ± 5.3	35.8 ± 7.1*	31.3 ± 5.0	38.5 ± 7.7*	32.9 ± 4.0
RVSVi(mL/m^2^)	46.6 ± 6.0*	43.7 ± 4.9	45.0 ± 6.8	44.5 ± 5.6	47.2 ± 5.1*	43.6 ± 4.9	48.2 ± 6.2*	43.2 ± 3.9

The data are presented as the mean ± standard deviation. *LV* left ventricle, *RV* right ventricle, *LVEDV* left ventricular end-diastolic volume, *LVEDVi* left ventricular end-diastolic volume index, *LVEF* left ventricular ejection fraction, *LVESV* left ventricular end-systolic volume, *LVESVi* left ventricular end-systolic volume index, *LVM* left ventricular mass, *LVMi* left ventricular mass index, *LVSV* left ventricular stroke volume, *LVSVi* left ventricular stroke volume index, *RVEDV* right ventricular end-diastolic volume; *RVEDVi* right ventricular end-diastolic volume index, *RVEF* right ventricular ejection fraction, *RVESV* right ventricular end-systolic volume, *RVESVi* right ventricular end-systolic volume index, *RVSV* right ventricular stroke volume, *RVSVi* right ventricular stroke volume index

* P < 0.05 vs. girls.

**Table 3:** Morphology and function parameters of the LV and RV with exclusion of trabeculations and papillary muscles in ventricular volume.

	Total		4–8 years		9–13 years		14–18 years	
	Boys (n = 102)	Girls (n = 89)	Boys (n = 35)	Girls (n = 23)	Boys (n = 51)	Girls (n = 51)	Boys (n = 16)	Girls (n = 15)
LVEDV(mL)	84.5 ± 27.8	80.5 ± 18.6	59.1 ± 12.9	64.9 ± 13.3	91.8 ± 22.2*	81.5 ± 15.6	116.6 ± 20.2*	101.0 ±12.6
LVESV(mL)	27.6 ± 10.7	26.7 ± 7.1	18.8 ± 4.5	20.8 ± 4.5	30.1 ± 9.3	27.2 ± 5.7	38.9 ± 9.8	34.2 ± 7.0
LVSV(mL)	56.9 ± 17.8	53.8 ± 12.1	40.3 ± 8.8	44.1 ± 9.6	61.8 ± 13.6*	54.3 ± 10.5	77.7 ± 11.9*	66.8 ± 6.9
LVEF(%)	67.7 ± 3.1	66.9 ± 3.0	68.3 ± 2.4	67.9 ± 3.4	67.6 ± 3.1	66.7 ± 2.7	66.9 ± 4.1	66.4 ± 3.5
LVM(g)	59.7 ± 20.6*	52.3 ± 11.9	41.0 ± 11.0	41.5 ± 7.3	65.5 ± 16.5*	53.5 ± 10.4	82.0 ± 15.0*	64.8 ± 8.0
RVEDV(mL)	90.3 ± 32.0	84.3 ± 20.7	60.6 ± 14.2	66.1 ± 15.0	99.0 ± 24.5*	85.2 ± 16.3	127.7 ± 25.2*	109.0 ± 13.7
RVESV(mL)	34.0 ± 15.1	30.7 ± 9.8	21.3 ± 6.5	22.2 ± 6.7	37.6 ± 12.3*	31.1 ± 7.4	50.2 ± 15.4	42.6 ± 8.3
RVSV(mL)	56.4 ± 18.0	53.6 ± 12.1	39.3 ± 9.0	44.0 ± 9.5	61.4 ± 13.5*	54.1 ± 10.7	77.5 ± 12.0*	66.4 ± 6.6
RVEF(%)	63.3 ± 5.3	64.0 ± 4.7	65.1 ± 5.3	66.8 ± 4.6	62.6 ± 4.9	63.6 ± 4.5	61.4 ± 5.7	61.2 ± 3.7
LVEDVi(mL/m^2^)	68.5 ± 9.4*	65.7 ± 7.2	65.2 ± 9.2	65.0 ± 7.6	69.6 ± 8.9*	65.9 ± 7.2	72.5 ± 9.3*	66.3 ± 6.8
LVESVi(mL/m^2^)	22.0 ± 4.2	21.8 ± 3.3	20.7 ± 3.1	20.9 ± 3.3	22.6 ± 4.4	22.0 ± 3.1	24.1 ± 4.8	22.4 ± 4.0
LVSVi(mL/m^2^)	46.5 ± 6.2*	43.9 ± 4.9	44.5 ± 6.8	44.1 ± 5.5	47.0 ± 5.5*	43.9 ± 4.9	48.4 ± 6.0*	43.9 ± 4.0
LVMi(g/m^2^)	48.1 ± 7.2*	42.7 ± 4.3	44.8 ± 7.1	41.7 ± 3.8	49.5 ± 6.4*	43.2 ± 4.6	51.0 ± 7.7*	42.5 ± 4.0
RVEDVi(mL/m^2^)	72.9 ± 11.6*	68.6 ± 7.8	67.0 ± 11.9	66.0 ± 8.5	74.9 ± 9.8*	68.9 ± 7.5	79.1 ± 11.4*	71.5 ± 6.8
RVESVi(mL/m^2^)	27.1 ± 7.1*	24.8 ± 5.1	23.7 ± 6.8	22.0 ± 4.8	28.2 ± 6.2*	25.2 ± 4.8	30.9 ± 7.6*	27.9 ± 4.4
RVSVi(mL/m^2^)	45.8 ± 6.2*	43.8 ± 4.7	43.3 ± 6.6	44.0 ± 5.4	46.7 ± 5.4*	43.7 ± 4.7	48.3 ± 6.2*	43.6 ± 3.7

The data are presented as the mean ± standard deviation. *LV* left ventricle, *RV* right ventricle, *LVEDV* left ventricular end-diastolic volume, *LVEDVi* left ventricular end-diastolic volume index, *LVEF* left ventricular ejection fraction, *LVESV* left ventricular end-systolic volume, *LVESVi* left ventricular end-systolic volume index, *LVM* left ventricular mass, *LVMi* left ventricular mass index, *LVSV* left ventricular stroke volume, *LVSVi* left ventricular stroke volume index, *RVEDV* right ventricular end-diastolic volume, *RVEDVi* right ventricular end-diastolic volume index, *RVEF* right ventricular ejection fraction, *RVESV* right ventricular end-systolic volume, *RVESVi* right ventricular end-systolic volume index, *RVSV* right ventricular stroke volume, *RVSVi* right ventricular stroke volume index

* P < 0.05 vs. girls.

**Fig. 3:**
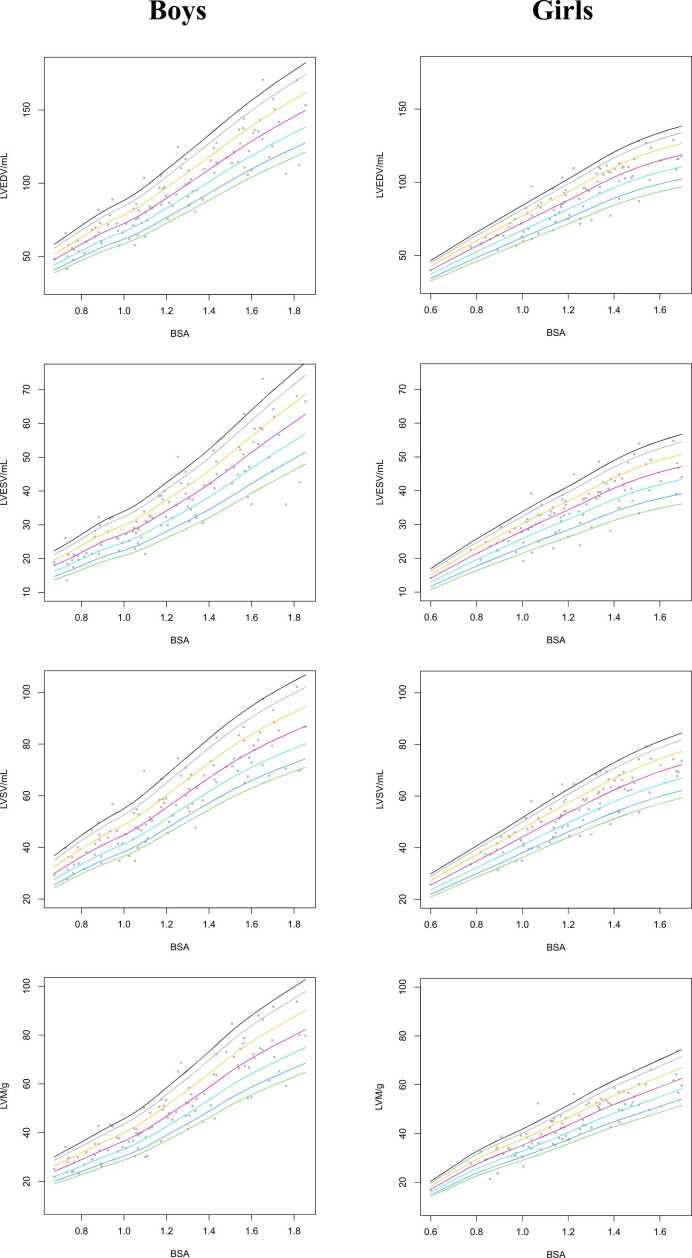
Reference percentiles curves for LVEDV, LVESV, LVSV, and LVM by BSA for boys (left column) and girls (right column). *LVEDV* left ventricular end-diastolic volume, *LVESV* left ventricular end-systolic volume, *LVSV* left ventricular stroke volume, *LVM* left ventricular mass, *BSA* body surface area

**Fig. 4:**
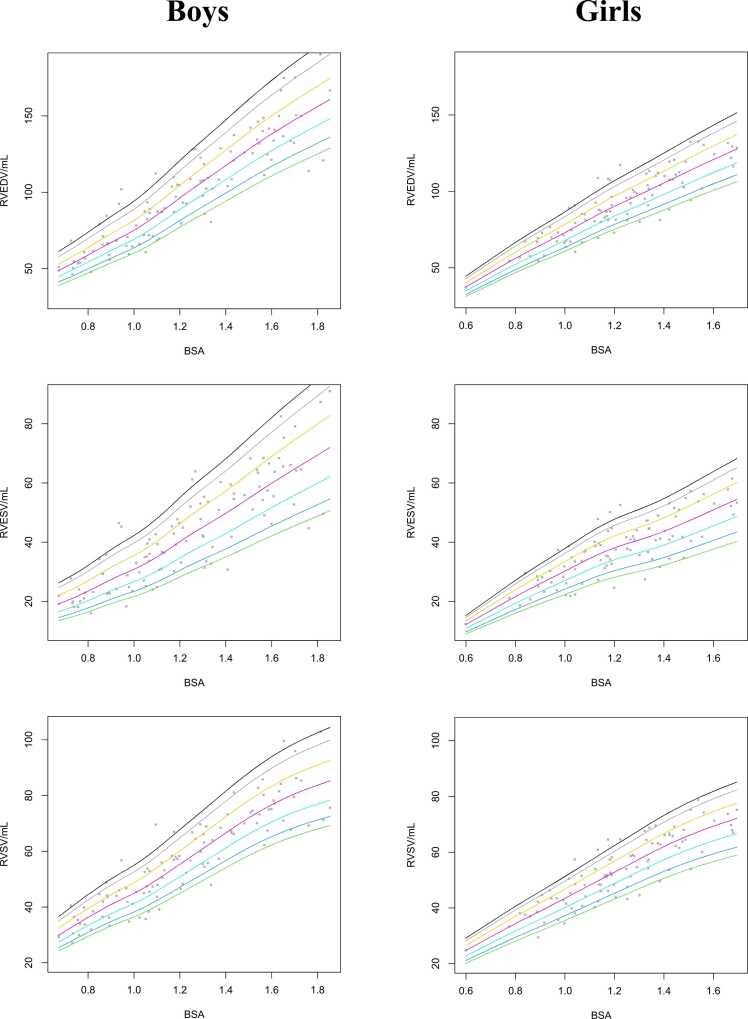
Reference percentiles curves for RVEDV, RVESV, and RVSV by BSA for boys (left column) and girls (right column). *RVEDV* right ventricular end-diastolic volume, *RVESV* right ventricular end-systolic volume, *RVSV* right ventricular stroke volume, *BSA* body surface area

For LV and RV structure, with the inclusion of trabeculations and papillary muscles in ventricular volume, percentile curves based on sex and BSA are shown in Fig. 3, Fig. 4, respectively. These percentile curves show non-linear, complex, relationships between BSA and parameters. Smoothed reference values for LV volumes and masses based on sex and BSA for the 5, 10, 25, 50, 75, 90, and 95 percentiles are presented in Table 4, Table 5, Table 6, Table 7. Sex- and age-specific percentile curves for ventricular parameters with the inclusion of trabeculations and papillary muscles in ventricular volume are shown in Supplemental Figs. S3-S4. Furthermore, centile tables for RVEDV, RVESV, and RVSV stratified based on BSA are provided in Supplemental Tables S2–S4**.** Supplemental Tables S5–S11 present the corresponding centile tables for biventricular structure stratified based on age and sex.

**Table 4:** Centiles of LVEDV with inclusion of trabeculations and papillary muscles in ventricular volume by BSA and sex.

	Boys							Girls						
BSA	5th	10th	25th	50th	75th	90th	95th	5th	10th	25th	50th	75th	90th	95th
0.6	34.0	35.8	38.8	42.0	45.4	48.8	51.1	32.5	34.4	37.2	39.9	42.6	45.0	46.6
0.7	40.6	42.7	46.3	50.1	54.2	58.3	61.0	39.4	41.6	45.0	48.3	51.5	54.5	56.3
0.8	47.2	49.7	53.9	58.4	63.1	67.9	71.1	46.0	48.6	52.6	56.4	60.2	63.6	65.8
0.9	53.5	56.4	61.1	66.1	71.4	76.9	80.5	52.4	55.4	59.9	64.3	68.6	72.5	75.0
1.0	58.6	61.8	67.0	72.5	78.3	84.3	88.2	58.9	62.2	67.3	72.2	77.0	81.5	84.2
1.1	64.9	68.4	74.2	80.3	86.7	93.4	97.7	65.3	69.0	74.6	80.1	85.4	90.3	93.4
1.2	72.6	76.5	83.0	89.8	97.0	104.4	109.3	71.5	75.6	81.7	87.8	93.6	99.0	102.3
1.3	80.4	84.7	91.8	99.4	107.4	115.6	121.0	78.0	82.5	89.2	95.8	102.1	108.0	111.6
1.4	88.4	93.1	100.9	109.3	118.0	127.1	133.0	84.3	89.1	96.3	103.4	110.3	116.7	120.6
1.5	96.4	101.6	110.1	119.2	128.8	138.6	145.1	89.5	94.6	102.3	109.8	117.1	123.9	128.0
1.6	104.0	109.6	118.8	128.6	139.0	149.6	156.6	93.6	98.9	106.9	114.8	122.4	129.5	133.9
1.7	111.1	117.1	126.9	137.4	148.4	159.7	167.2	96.9	102.4	110.8	118.9	126.8	134.1	138.6
1.8	117.6	124.0	134.3	145.4	157.1	169.1	177.1	100.2	105.8	114.4	122.9	131.0	138.6	143.3
1.9	124.1	130.8	141.7	153.5	165.8	178.5	186.8	103.4	109.2	118.1	126.9	135.2	143.1	147.9

The data are presented as the absolute value. *BSA* body surface area, *LVEDV* left ventricular end-diastolic volume

**Table 5:** Centiles of LVESV with inclusion of trabeculations and papillary muscles in ventricular volume by BSA and sex.

	Boys							Girls						
BSA	5th	10th	25th	50th	75th	90th	95th	5th	10th	25th	50th	75th	90th	95th
0.6	12.1	13.0	14.4	15.8	17.3	18.7	19.7	11.0	11.9	13.2	14.4	15.5	16.6	17.3
0.7	14.3	15.4	17.0	18.7	20.5	22.2	23.3	13.8	14.9	16.5	18.0	19.4	20.7	21.6
0.8	16.7	17.9	19.8	21.8	23.8	25.8	27.1	16.4	17.7	19.7	21.4	23.1	24.7	25.8
0.9	19.1	20.5	22.7	25.0	27.3	29.6	31.1	18.9	20.5	22.7	24.7	26.6	28.5	29.7
1.0	20.9	22.4	24.8	27.3	29.9	32.4	34.0	21.5	23.2	25.8	28.1	30.2	32.4	33.7
1.1	23.2	24.9	27.6	30.3	33.2	35.9	37.8	23.9	25.9	28.8	31.3	33.7	36.1	37.6
1.2	26.1	28.1	31.0	34.2	37.4	40.5	42.5	26.3	28.4	31.5	34.3	37.0	39.6	41.3
1.3	29.0	31.2	34.5	38.0	41.5	45.0	47.3	28.7	31.1	34.5	37.6	40.5	43.3	45.2
1.4	32.2	34.5	38.2	42.1	46.0	49.8	52.4	31.2	33.8	37.4	40.7	43.9	47.0	49.0
1.5	35.7	38.3	42.4	46.7	51.0	55.3	58.1	33.2	36.0	39.9	43.4	46.8	50.1	52.2
1.6	39.3	42.2	46.7	51.4	56.2	60.9	64.0	34.8	37.7	41.8	45.5	49.0	52.5	54.8
1.7	42.8	46.0	50.9	56.0	61.3	66.4	69.7	36.2	39.2	43.5	47.3	50.9	54.6	56.9
1.8	46.2	49.6	54.9	60.4	66.1	71.5	75.2	37.5	40.6	45.0	49.0	52.8	56.5	58.9
1.9	49.5	53.2	58.8	64.8	70.8	76.7	80.6	38.8	42.0	46.6	50.7	54.6	58.5	61.0

The data are presented as the absolute value. *BSA* body surface area; *LVESV* left ventricular end-systolic volume

**Table 6:** Centiles of LVSV with inclusion of trabeculations and papillary muscles in ventricular volume by BSA and sex.

	Boys							Girls						
BSA	5th	10th	25th	50th	75th	90th	95th	5th	10th	25th	50th	75th	90th	95th
0.6	21.4	22.4	24.2	26.3	28.5	30.8	32.3	21.0	22.0	23.7	25.5	27.4	28.9	29.9
0.7	25.6	26.8	28.9	31.4	34.1	36.8	38.5	24.8	26.0	28.0	30.2	32.3	34.2	35.3
0.8	29.7	31.1	33.5	36.4	39.5	42.7	44.7	28.6	30.0	32.3	34.8	37.3	39.5	40.7
0.9	33.4	35.0	37.7	40.9	44.5	48.0	50.3	32.4	33.9	36.5	39.4	42.2	44.7	46.1
1.0	36.6	38.3	41.3	44.9	48.7	52.6	55.1	36.2	38.0	40.9	44.1	47.2	50.0	51.6
1.1	40.6	42.5	45.8	49.7	54.0	58.3	61.1	40.2	42.2	45.4	49.0	52.4	55.5	57.3
1.2	45.3	47.4	51.1	55.5	60.2	65.0	68.1	44.0	46.1	49.7	53.6	57.4	60.7	62.7
1.3	50.0	52.3	56.4	61.2	66.5	71.8	75.2	47.6	50.0	53.8	58.0	62.2	65.8	67.9
1.4	54.6	57.2	61.7	66.9	72.7	78.5	82.2	51.2	53.7	57.8	62.4	66.8	70.7	72.9
1.5	59.1	61.8	66.6	72.3	78.6	84.8	88.9	54.3	57.0	61.4	66.2	70.9	75.0	77.4
1.6	63.0	65.9	71.1	77.1	83.8	90.4	94.7	57.0	59.8	64.4	69.5	74.4	78.7	81.2
1.7	66.4	69.5	74.9	81.3	88.3	95.3	99.9	59.4	62.3	67.1	72.3	77.4	81.9	84.6
1.8	69.4	72.7	78.3	85.0	92.3	99.7	104.5	61.7	64.7	69.7	75.1	80.5	85.1	87.8
1.9	72.4	75.7	81.7	88.6	96.3	103.9	108.9	64.0	67.1	72.3	77.9	83.5	88.3	91.1

The data are presented as the absolute value. *BSA* body surface area, *LVSV* left ventricular stroke volume

**Table 7:** Centiles of LVM with inclusion of trabeculations and papillary muscles in ventricular volume by BSA and sex.

	Boys							Girls						
BSA	5th	10th	25th	50th	75th	90th	95th	5th	10th	25th	50th	75th	90th	95th
0.6	16.8	17.8	19.4	21.4	23.4	25.4	26.7	14.1	14.8	16.0	17.2	18.4	19.6	20.4
0.7	19.8	21.0	22.9	25.2	27.6	29.9	31.4	18.6	19.5	21.0	22.6	24.2	25.8	26.9
0.8	22.8	24.1	26.3	28.9	31.7	34.4	36.1	22.6	23.7	25.6	27.5	29.4	31.4	32.7
0.9	25.9	27.4	29.9	32.9	36.0	39.1	41.0	25.9	27.2	29.3	31.5	33.7	36.0	37.4
1.0	28.8	30.4	33.3	36.6	40.1	43.5	45.7	29.0	30.4	32.7	35.2	37.7	40.2	41.8
1.1	32.3	34.2	37.4	41.1	45.0	48.8	51.3	32.3	33.9	36.5	39.2	42.0	44.8	46.6
1.2	36.8	38.9	42.6	46.7	51.2	55.6	58.4	35.7	37.5	40.4	43.4	46.5	49.6	51.6
1.3	41.4	43.7	47.8	52.5	57.5	62.5	65.6	39.4	41.4	44.5	47.8	51.2	54.7	56.9
1.4	46.2	48.8	53.4	58.6	64.2	69.7	73.2	42.7	44.9	48.3	51.9	55.6	59.3	61.7
1.5	51.1	54.1	59.1	65.0	71.2	77.3	81.2	45.7	48.0	51.7	55.5	59.5	63.5	66.0
1.6	55.5	58.7	64.2	70.5	77.2	83.9	88.1	48.7	51.1	55.0	59.1	63.3	67.6	70.3
1.7	59.3	62.7	68.6	75.3	82.5	89.6	94.1	51.7	54.2	58.4	62.8	67.2	71.7	74.6
1.8	62.9	66.5	72.7	79.9	87.5	95.0	99.8	54.6	57.4	61.8	66.4	71.1	75.8	78.9
1.9	66.4	70.2	76.8	84.4	92.4	100.4	105.4	57.6	60.5	65.2	70.0	75.0	80.0	83.2

The data are presented as the absolute value. *BSA* body surface area, *LVM* left ventricular mass

For LV and RV structure with the exclusion of trabeculations and papillary muscles in ventricular volume, we provide additional percentile curves and tables for boys and girls (Supplemental Figs. S5–S8 and Supplemental Tables S12–S25).

### 3.3 Reference values of atrial size parameters

**Fig. 5:**
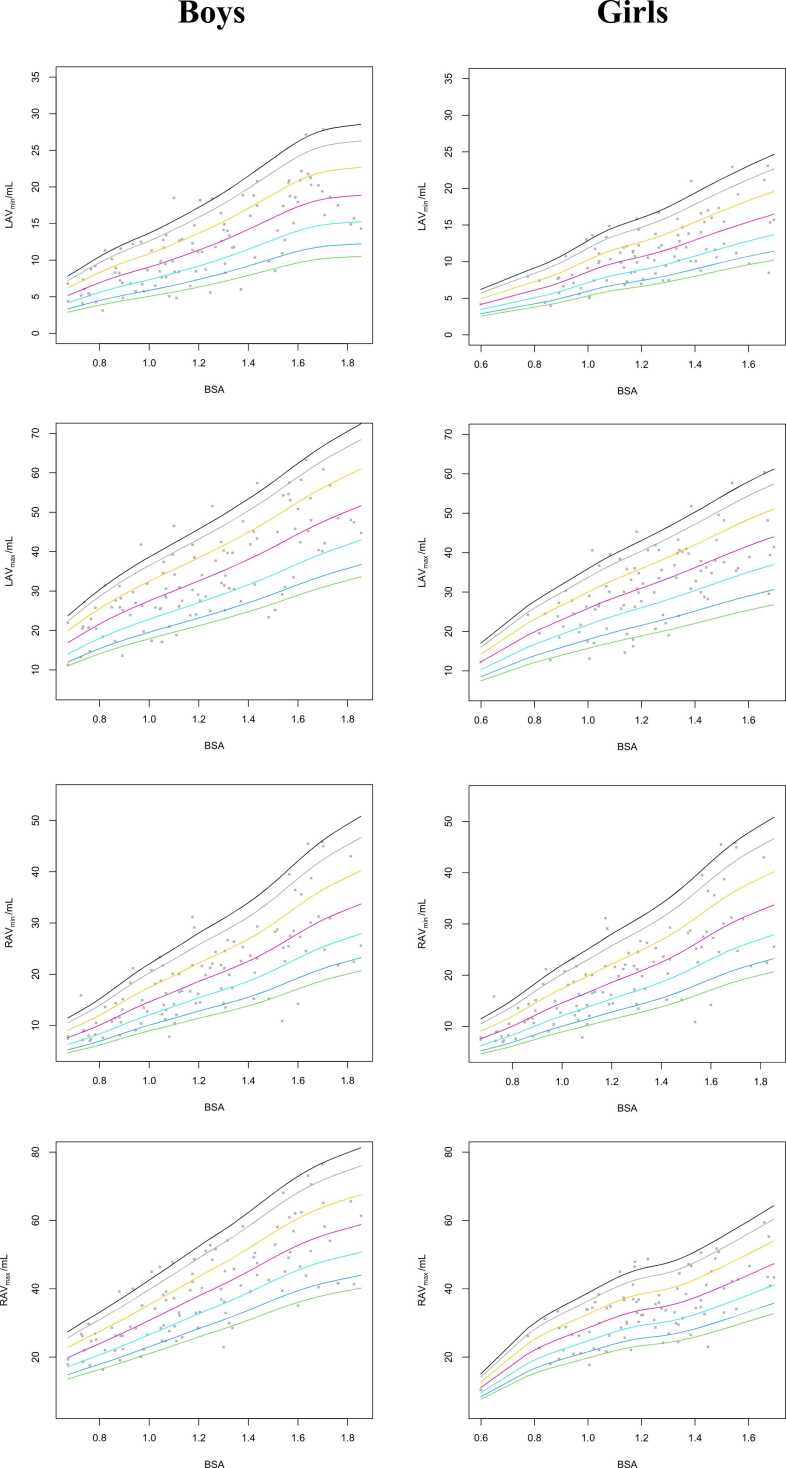
Reference percentiles curves for LAV_min_, LAV_max_, RAV_min_, and RAV_max_ by BSA for boys (left column) and girls (right column). *LAV_min_* minimal left atrial volume, *LAV_max_* maximal left atrial volume, *RAV_min_* minimal right atrial volume, *RAV_max_* maximal right atrial volume, *BSA* body surface area

Absolute values and values indexed to BSA for LA and RA volumes are presented in Table 8. In the youngest age group, significant differences attributable to the subject’s sex were not observed in absolute or indexed LA and RA volumes. In the other old age groups, LAV_max_, LAV_min,_ RAV_max_, and RAV_min_ were significantly higher in boys than those in girls. However, after BSA normalization, only RAV_max_ remained significantly greater in boys than in girls. Percentile curves and tables for LA and RA volumes are presented in Fig. 5**,** Supplemental Fig. S9**,** and Supplemental Tables S26–S33**.**

**Table 8:** Volumetric parameters of the LA and RA.

	Total		4–8 years		9–13 years		14–18 years	
	Boys (n = 102)	Girls (n = 89)	Boys (n = 35)	Girls (n = 23)	Boys (n = 51)	Girls (n = 51)	Boys (n = 16)	Girls (n = 15)
LAV_min_	12.2 ± 5.5	11.2 ± 4.1	7.7 ± 2.6	8.5 ± 2.8	13.3 ± 4.6*	11.4 ± 3.7	18.5 ± 4.8*	14.9 ± 4.2
LAV_max_	33.9 ± 12.0	31.6 ± 10.0	24.3 ± 7.0	25.9 ± 7.1	36.2 ± 10.1*	31.6 ± 9.6	47.4 ± 10.1*	40.0 ± 9.4
RAV_min_	19.7 ± 9.2	17.8 ± 6.6	12.8 ± 4.7	13.2 ± 4.8	21.3 ± 8.3*	18.0 ± 5.5	29.8 ± 7.8*	24.1 ± 7.1
RAV_max_	39.2 ± 14.1*	34.1 ± 9.4	27.0 ± 6.9	28.4 ± 7.5	43.2 ± 12.6*	33.7 ± 8.0	53.1 ± 9.5*	44.2 ± 8.7
iLAV_min_	9.7 ± 2.9	9.1 ± 2.5	8.5 ± 2.6	8.5 ± 2.4	10.1 ± 2.9	9.2 ± 2.4	11.5 ± 2.8	9.8 ± 2.8
iLAV_max_	27.7 ± 6.4	25.8 ± 6.2	26.9 ± 6.8	26.0 ± 5.9	27.7 ± 6.2	25.5 ± 6.6	29.6 ± 6.2	26.3 ± 5.8
iRAV_min_	15.7 ± 4.7	14.3 ± 3.9	13.9 ± 3.8	13.0 ± 3.6	16.0 ± 4.9	14.5 ± 3.7	18.6 ± 4.3	15.8 ± 4.4
iRAV_max_	31.9 ± 6.8*	28.0 ± 5.9	29.8 ± 6.2	28.5 ± 6.0	32.8 ± 7.3*	27.5 ± 6.0	33.2 ± 5.8*	29.0 ± 5.3

The data are presented as the mean ± standard deviation. *LAV_min_* minimal left atrial volume, *LAV_max_* maximal left atrial volume, *RAV_min_* minimal right atrial volume, *RAV_max_* maximal right atrial volume, *iLAV_min_* minimal left atrial volume index, *iLAV_max_* maximal left atrial volume index, *iRAV_min_* minimal right atrial volume index, *iRAV_max_* maximal right atrial volume index

*P < 0.05 vs. girls.

### 3.4 Intra-observer and inter-observer variability

Intra- and inter-observer variability is summarized in Supplemental Tables S34–S36. Intra-observer agreement was good, with the highest variation in measurements in LVESV, RVESV, and LVM. The variability in atrial volume measurements ranged from 9.8% to 12.1% for intra-observer assessment. Inter-observer agreement demonstrated greater variation for all variables. The limits of agreement were wider, and the COV was higher for ventricular parameters determined using the smooth segmentation method compared to those determined using the anatomic segmentation method.

### 3.5 Comparison with previous studies

Previous CMR studies reporting normal ranges of ventricular and atrial size and function in children were summarized in Table 9. Overall, eight studies from Caucasian populations met the predefined selection criteria and were included in the meta-analysis (highlighted in gray). Several studies reported results separately for boys and girls, and each sub-study was treated as an independent data source in this meta-analysis. Although Ishikawa’s study [24] included only 23 participants, it was included in the meta-analysis due to its unique focus on normal ventricular size in Asian children. Only one study [14] included trabeculations and papillary muscles in the ventricular volume, so comparisons for ventricular sizes were performed in studies with exclusion of trabeculations and papillary muscles in ventricular volume. Results for ethnicity-stratified analyses are summarized in Table 10 and Supplemental Figs. S10–S11. Asian children had significantly lower ventricular and atrial volumes than those observed in Caucasian children (all *p*<0.05).

**Table 9:** Summary of previous published data of cardiac volumes and function for healthy children.

Author	Year	Journal	N	Age	Male (%)	Ethnicity	Field (T)	volume	Contouring method
Robbers-Visser [6]	2009	JMRI	60	Range 8–17 years	50	Caucasian	1.5	LV, RV	Inclusion of trabeculations and papillary muscles in ventricular mass
Buechel [7]	2009	JCMR	50	Median 11 years (Range 0.7–18 years)	46	Caucasian	1.5	LV, RV	Inclusion of trabeculations and papillary muscles in ventricular volume
Sarikouch [8]	2010	Circ Cardiovasc Imaging	114	12.4 ± 4.1 years (Range 4–18 years)	48	Caucasian	1.5	LV, RV	Inclusion of trabeculations and papillary muscles in ventricular mass
van der Ven [9]	2019	Eur Heart J Cardiovasc Imaging	141	Median 12.7 years (0.6–18.5)	48	Caucasian	1.5	LV, RV	Inclusion of trabeculations and papillary muscles in LV mass and RV volume
Olivieri [10]	2020	JCMR	149	5.1 ± 3.6 years (Range 0–12 years)	47	Most Caucasian	1.5	LV, RV	Inclusion of trabeculations and papillary muscles in ventricular volume
Krupickova [11]	2021	JCMR	161	13.6 ± 2.9 years (Range 6–18 years)	60.3	Caucasian	1.5	LV, RV	Exclusion of trabeculations and papillary muscles from volume/mass
Voges [12]	2023	JMRI	154	13.9 ± 2.8 years	66	Caucasian	1.5	LV, RV	Inclusion of trabeculations and papillary muscles in ventricular volume/mass
Jhaveri [13]	2023	JCMR	20	31.8 ± 14 h (Range 9–56 h)	65	Caucasian	3.0	LV, RV	Inclusion of trabeculations and papillary muscles in LV mass
Real [14]	2023	EClinicalMedicine	123	16 ± 0.5 years (Range 15–18 years)	48	Caucasian	3.0	LV, RV, LA	Inclusion of trabeculations and papillary muscles in ventricular volume and Biplane Area-Length Methods for LA
Henderson [15]	2023	JCMR	96	14.3 ± 3.4 years	55	Most Caucasian	1.5	LA	Biplane Area-Length Methods
Sarikouch [16]	2011	JMRI	115	12.4 ± 4.1 years (Range 4–18 years)	49	Caucasian	1.5	LA, RA	Semi-automatic threshold-based atrial volumetric analysis
Voges [17]	2023	JMRI	155	13.9 ± 2.8 years	66	Caucasian	1.5	LA, RA	Monoplane and Biplane Area-Length Methods
Ishikawa [24]	2023	Int Heart J	23	7.2 ± 4.9	65	East Asian	1.5	LV, RV	Inclusion of trabeculations and papillary muscles in ventricular mass

*LV* left ventricle, *RV* right ventricle, *LVEDV* left ventricular end-diastolic volume, *LVEDVi* left ventricular end-diastolic volume index, *LVEF* left ventricular ejection fraction, *LVESV* left ventricular end-systolic volume, *LVESVi* left ventricular end-systolic volume index, *LVM* left ventricular mass, *LVMi* left ventricular mass index, *LVSV* left ventricular stroke volume, *LVSVi* left ventricular stroke volume index, *RVEDV* right ventricular end-diastolic volume; *RVEDVi* right ventricular end-diastolic volume index, *RVEF* right ventricular ejection fraction, *RVESV* right ventricular end-systolic volume, *RVESVi* right ventricular end-systolic volume index, *RVSV* right ventricular stroke volume, *RVSVi* right ventricular stroke volume index, *LAVmin* minimal left atrial volume, *LAVmax* maximal left atrial volume, *RAVmin* minimal right atrial volume, *RAVmax* maximal right atrial volume, *iLAVmin* minimal left atrial volume index, *iLAVmax* maximal left atrial volume index, *iRAVmin* minimal right atrial volume index, *iRAVmax* maximal right atrial volume index

**Table 10:** Pooled mean ventricular and atrial parameters with ethnicity stratification and expression of between-study heterogeneity and subgroup differences.

Parameters	Ethnicity	N	Mean (95%CI)	Between-study heterogeneity		Subgroup differences (Caucasians vs. Asians	
				H^2^	I^2^	Mean difference	P
LVEDVI	Caucasians	476	75.22(67.22,83.22)	1.00	0.00%	8.39	<0.001
	Asians	214	66.83(52.98,80.67)	1.00	0.00%		
LVESVI	Caucasians	476	25.13(20.92, 29.33)	1.00	0.00%	2.51	<0.001
	Asians	214	22.62(16.35,28.88)	1.00	0.00%		
LVSVI	Caucasians	416	48.50(41.89,55.11)	1.00	0.00%	3.30	<0.001
	Asians	191	45.20(34.03,56.37)	-	-		
LVMI	Caucasians	315	55.27(46.62,63.92)	1.00	0.00%	11.26	<0.001
	Asians	214	44.01(34.14,53.89)	1.00	0.00%		
RVEDVI	Caucasians	476	78.57(69.67,87.46)	1.00	0.00%	7.94	<0.001
	Asians	214	70.63(53.93,87.33)	1.00	0.00%		
RVESVI	Caucasians	476	29.37(24.59,34.14)	1.00	0.00%	3.16	<0.001
	Asians	214	26.21(16.29,36.13)	1.00	0.00%		
RVSVI	Caucasians	416	48.64(41.52,55.77)	1.00	0.00%	3.84	<0.001
	Asians	191	44.80(33.82,55.78)	-	-		
iLAV_min_	Caucasians	438	15.0(10.8,19.3)	1.00	0.00%	5.5	<0.001
	Asians	191	9.5(4.01,15.0)	-	-		
iLAV_max_	Caucasians	438	36.5(28.7,44.3)	1.00	0.00%	9.7	<0.001
	Asians	191	26.8(14.3,39.3)	-	-		
iRAV_min_	Caucasians	219	21.6(11.8,31.3)	1.00	0.00%	6.5	<0.001
	Asians	191	15.1(6.5,23.7)	-	-		
iRAV_max_	Caucasians	219	42.1(20.6, 63.6)	1.74	42.57%	12.1	<0.001
	Asians	191	30.0(16.87,43.13)	-	-		

*LV* left ventricle, *RV* right ventricle, *LVEDV* left ventricular end-diastolic volume, *LVEDVi* left ventricular end-diastolic volume index, *LVEF* left ventricular ejection fraction, *LVESV* left ventricular end-systolic volume, *LVESVi* left ventricular end-systolic volume index, *LVM* left ventricular mass, *LVMi* left ventricular mass index, *LVSV* left ventricular stroke volume, *LVSVi* left ventricular stroke volume index, *RVEDV* right ventricular end-diastolic volume, *RVEDVi* right ventricular end-diastolic volume index, *RVEF* right ventricular ejection fraction, *RVESV* right ventricular end-systolic volume, *RVESVi* right ventricular end-systolic volume index, *RVSV* right ventricular stroke volume, *RVSVi* right ventricular stroke volume index, *LAVmin* minimal left atrial volume, *LAVmax* maximal left atrial volume, *RAVmin* minimal right atrial volume, *RAVmax* maximal right atrial volume, *iLAVmin* minimal left atrial volume index, *iLAVmax* maximal left atrial volume index, *iRAVmin* minimal right atrial volume index, *iRAVmax* maximal right atrial volume inde

## 4. Discussion

This study presents age-, BSA-, and sex-specific ventricular and atrial reference ranges derived from healthy Chinese children. The primary contributions of our study can be summarized as follows: (1) To the best of our knowledge, although normal CMR reference ranges are available for Chinese adults [25], [26], [27], [28], [29], this is the first study to investigate reference ranges for ventricular and atrial volume and function in Chinese children. (2) This study considers variations in CMR ventricular contouring methods and provides coherent reference values for readers that habitually include or exclude trabeculations and papillary muscles in ventricular volume calculations. (3) This study presents the first meta-analysis of CMR normal reference ranges obtained from eight studies that included Asian and Caucasian children. The pooled results demonstrate significant differences in chamber size parameters by ethnicity. We believe that the present study may contribute to the accurate clinical diagnosis of various cardiovascular diseases.

Several multi-ethnic investigations have confirmed that East Asian adults typically exhibit smaller chamber sizes compared to those of Caucasian adults [30], [31]. Similarly, a recent meta-analysis highlighted that ventricular volumes among East Asian adults are notably lower than those of Caucasian adults, even after normalization for BSA [32]. Studies comparing normal CMR reference values for Asian and Caucasian children are lacking. Our study addresses this gap and finds that both atrial and ventricular sizes are greater in Caucasian children compared to those in Asian children.

Previous CMR studies involving 20–155 healthy children reported normal ranges of ventricular and atrial size and function across a broad age range [6], [7], [8], [9], [10], [11], [12], [13], [14], [15], [16], [17], [24]. Van der Ven *et al*. [9] collected data from a multi-center consortium of high-volume pediatric programs and conducted volumetric analyses in 141 healthy children. While that study represents a significant contribution to the field, it includes a relatively small sample size of children under 8 years of age (n = 19), which limits detailed analysis. Thus, we further expanded the sample size to 58 children within this age range and differences in volumes or masses between sexes in the youngest age group (4–8 years) were not found, indicating similar cardiac development between boys and girls attending preschool. Recently, Real *et al*. [14] established CMR reference values for adolescents (15–18 years) using a 3T scanner, including trabeculations and papillary muscles in LV volume calculations. Their analysis revealed elevated LV and RV volumes, as well as increased LV masses in boys, which is consistent with the results presented in this study. However, compared with results of that study, we found lower overall values for all indexed volumetric ventricular parameters in our oldest cohort (14–18 years).

Methodology used for image analysis is the most important technical factor contributing to heterogeneity across reference ranges, as it directly influences the derived measurements of cardiac function [5]. It has been demonstrated that diverse contouring methods can yield disparate CMR-derived volumetric outcomes [12], [32], [33]. Hence, it is essential to consider the segmentation methods employed when determining the most appropriate reference values. In our study, we observed that inclusion of trabeculations and papillary muscles in volume measurements resulted in larger ventricular volumes, smaller masses, and lower LVEF and RVEF measurements compared with the ones obtained using the anatomic segmentation method. These findings are consistent with those in published reports [12], [32], [33], confirming the impact of segmentation methods on CMR-derived measurements. Our study presents percentile curves and tables for both segmentation methods and provides reference values to help clinicians and researchers select the segmentation method that is typically favored for their practice. In addition, although both methods can be applied with good overall reproducibility, as shown in our reproducibility analysis, the anatomic segmentation method which includes papillary muscles and trabeculations in the ventricular volume demonstrated better performance. This suggests that the anatomic segmentation method may be a more reliable approach for application in children in our institution.

The measurement of atrial volumes is crucial in the treatment and follow-up of pediatric cardiac disorders, particularly in patients with congenital heart disease and atrioventricular valve disease [17]. A limited number of studies have reported CMR reference values for atrial volumes in healthy children [14], [15], [16], [17]. This is the first study to establish the reference values of LA and RA volumes in Asian pediatric populations. Compared with results of several studies conducted in Western populations [15], [16], [17], LA and RA volumetric parameters in our study were lower, which may further confirm differences attributable to ethnicity in atrial volumes. Additionally, boys exhibited greater absolute LA volumes but similar indexed LA volumes compared to girls, a finding that aligns with the results reported by Real et al. [14]. Interestingly, although the previous study suggested similar RA sizes between sexes, our study observed a trend toward greater iRAVmax in boys compared to that in girls, which could be attributed to differences in age distribution between the study populations.

Our study utilized a 3T scanner, as opposed to previous pediatric studies which predominantly used 1.5T scanners to examine cardiac chamber size and function [6], [7], [8], [9], [10], [11], [12], [24]. The higher signal-to-noise ratio and superior image resolution, along with reduced acquisition time offered by 3T CMR may make it more suitable for investigating the comparatively smaller hearts and higher heart rates of children, albeit at the cost of increased artifacts with steady-state free precession imaging. Nonetheless, in our study, only a minimal number of cases were excluded due to poor image quality or technical issues, underscoring the feasibility of conducting comprehensive, high-quality 3T CMR studies in pediatric populations.

## 5. Limitations

In this study, we did not include children under 4 years of age. Recruiting healthy volunteers from such populations is challenging, as children younger than 4 years typically require sedation or general anesthesia to undergo MRI examinations, making the indication for scanning much stricter. In addition, RV mass was not measured in this study due to concerns regarding the limited spatial resolution for accurately delineating thin RV walls in children. The challenge associated with tracing the thin RV wall is reflected in the relatively modest inter-observer agreement, as demonstrated in the study by van der Ben et al. [9]. Third, automated segmentation was not used for the RV, which may be related to the increased inter- and intra- observer variability. However, this decision was based on preliminary assessments that indicated suboptimal performance of automated segmentation tools in accurately capturing RV contours. Finally, we did not perform a meta-analysis on subgroups based on ethnicity for boys and girls separately. On the one hand, some studies did not report such data. On the other hand, sample sizes for analysis would be limited upon further subdivision of groups based on sex. Therefore, there is an urgent need for more CMR studies focusing on normal reference values for children to enable a more comprehensive analysis.

## 6. Conclusion

Our study provides normative reference values for CMR cardiac size and function in Chinese children based on BSA, age, and sex. Furthermore, we confirm that differences in cardiac size based on ethnicity can be found even in children. Our findings are valuable for clinical practice, as they can serve as a reference standard for the diagnosis of acquired and congenital heart diseases in children.

## Funding

This work was supported by 10.13039/501100001809National Natural Science Foundation of China (82120108015, 82102020, 82071874, 824B2052, 82271981), Universal Application Project of Health Commission of Sichuan Province (21PJ048), Sichuan Science and Technology Program (2020YJ0029, 2017TD0005, 2021YFS0175, 2023ZYD0121, 2023YFG0284, 24NSFSC1085), Clinical Research Finding of Chinese Society of Cardiovascular Disease (CSC) of 2019 (No. HFCSC2019B01), and 10.13039/501100012226Fundamental Research Funds for the Central Universities (SCU2020D4132).

## Author contributions

**Ke Xu**: Conceptualization, Methodology, Formal analysis, Writing - original draft, Writing - review and editing, Project administration. **Wei Bai:** Conceptualization, Investigation, Data curation, Writing - original draft, Writing - review and editing, Supervision. **Zhi Yang:** Resources, Validation. **Rong Xu:** Resources, Validation. **Lin-jun Xie:** Data curation, Formal analysis, Writing - review and editing. **Ling-yi Wen:** Formal analysis, Writing - review and editing. **Chuan Fu:** Methodology, Investigation, Data curation. **Jie-qian Zheng:** Methodology, Investigation, Data curation. **Xin-mao Ma:** Methodology, Investigation, Data curation. **Hang Fu:** Formal analysis, Visualization. **Zhong-qin Zhou:** Formal analysis, Visualization. **Cheng-cheng Zhu:** Writing - review and editing. **Xiao-yue Zhou:** Methodology, Validation. **Hua-yan Xu:** Conceptualization, Supervision, Project administration, Writing - review and editing. **Ying-kun Guo:** Conceptualization, Supervision, Project administration, Funding acquisition.

## Ethics approval and consent

The study was performed in accordance with the Declaration of Helsinki and was approved by the Ethics Committee of West China Second University Hospital of Sichuan University (No.2022–105). Written informed consent was obtained from the children’s parents or legal guardians.

## Consent for publication

Not applicable.

## Declaration of competing interests

The authors declare that they have no known competing financial interests or personal relationships that could have appeared to influence the work reported in this paper.

## Footnotes

Appendix A Supplementary data associated with this article can be found in the online version at doi:10.1016/j.jocmr.2025.101885.
