# Supplementary material for: Age-, body surface area-, and sex-specific reference values for cardiovascular magnetic resonance imaging derived ventricular and atrial size and function for Chinese healthy children
Source: J Cardiovasc Magn Reson. 2025 Mar 21;27(1):101885. doi: 10.1016/j.jocmr.2025.101885 (PMC12182818; doi:10.1016/j.jocmr.2025.101885)

**Supplementary Figures**

**Supplementary Figure S1. Contouring method with exclusion of trabeculations and papillary muscles in the ventricular volume at end-systole and end-diastole.**


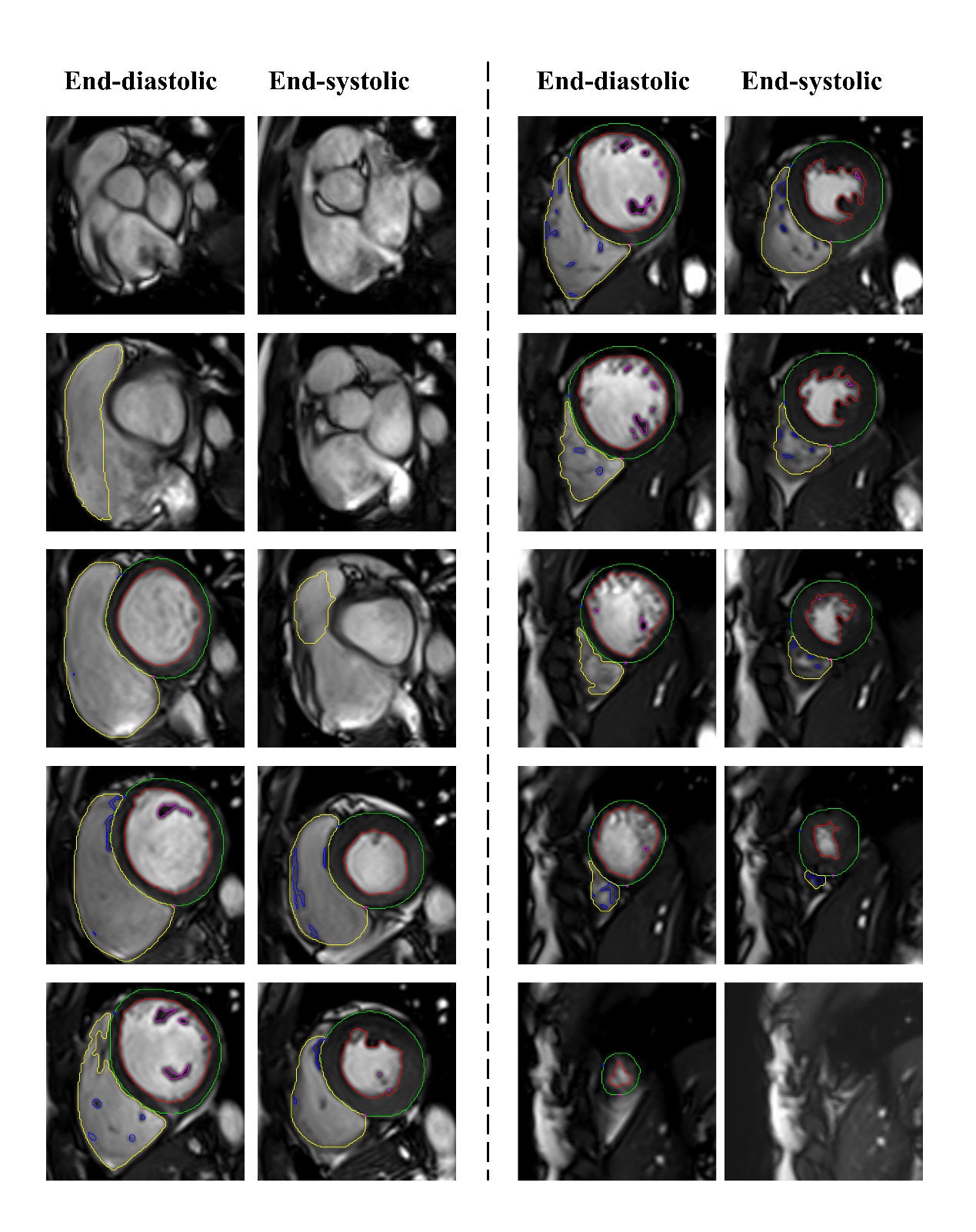


**Supplementary Figure S2. Example of atrial volumetric analysis in the 2- and 4-chamber views. Yellow contours for left atrium and blue contours for right atrium.**


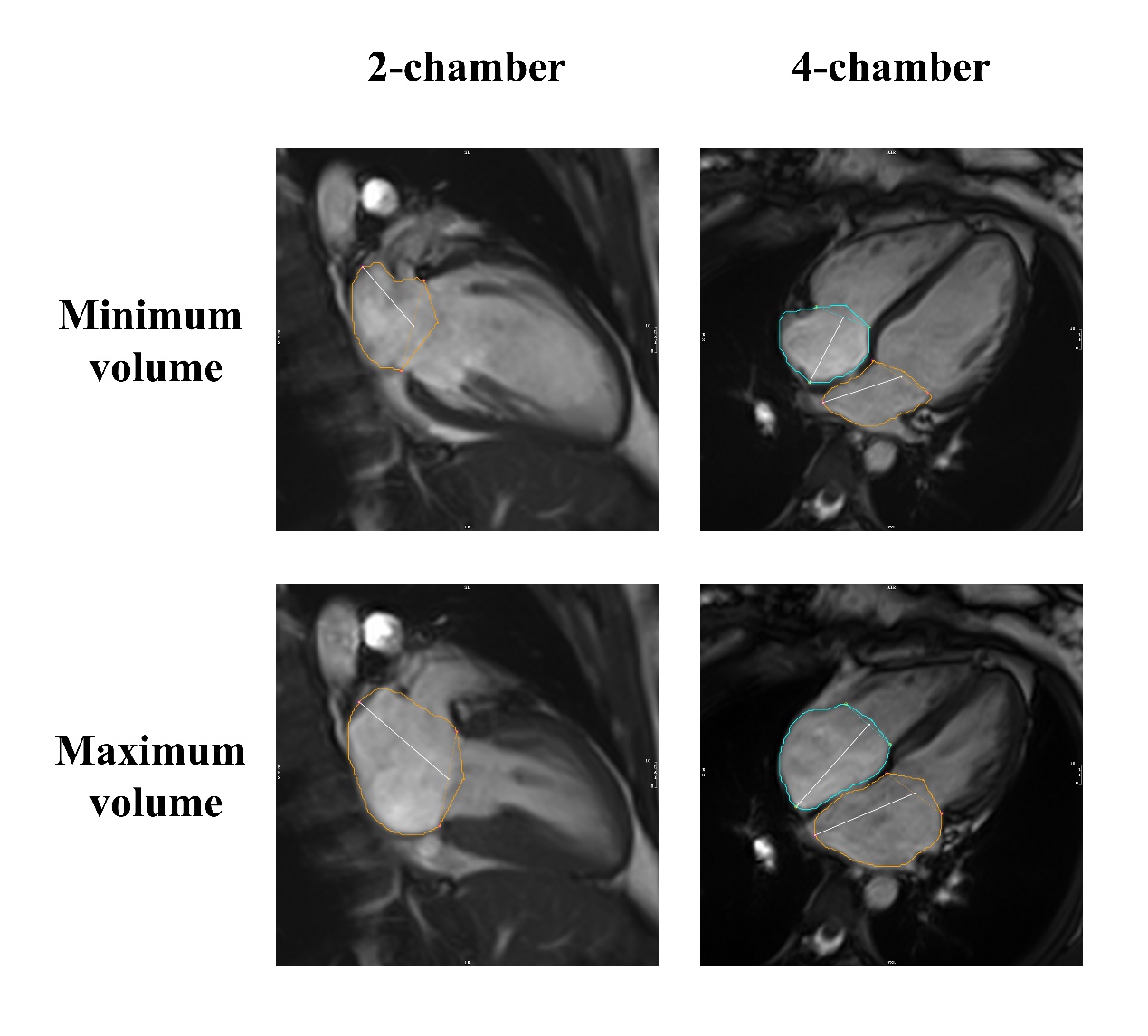


**Supplementary Figure S3**. **Reference percentiles curves for LVEDV, LVESV, LVSV, and LVM by age for boys (left column) and girls (right column) with inclusion of trabeculations and papillary muscles in the volume.** LVEDV: Left ventricular end diastolic volume; LVESV: Left ventricular end systolic volume; LVSV: Left ventricular stroke volume; LVM: Left ventricular mass


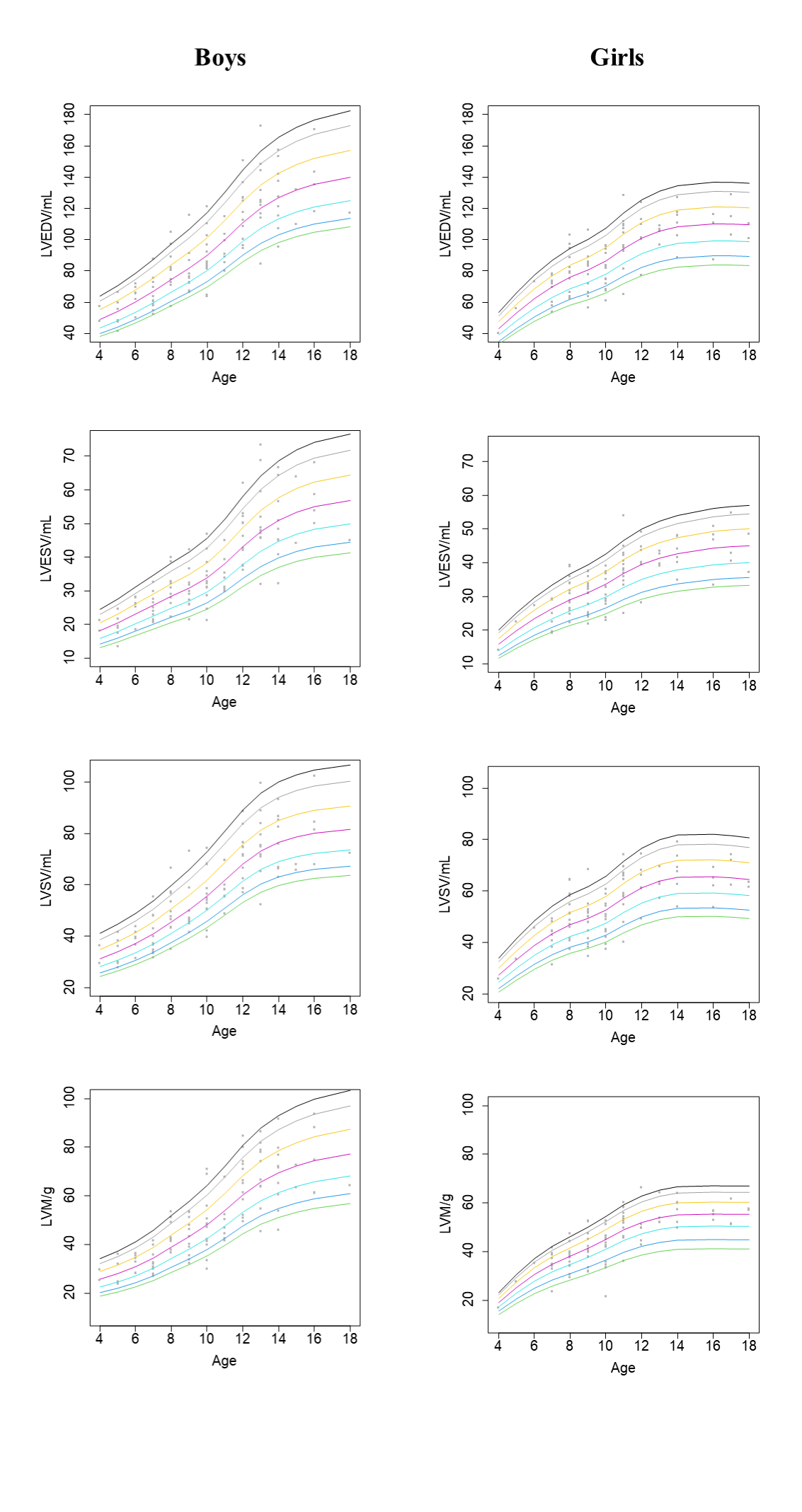


**Supplementary Figure S4**. **Reference percentiles curves for RVEDV, RVESV, and RVSV by age for boys (left column) and girls (right column) with inclusion of trabeculations and papillary muscles in the volume.** RVEDV: right ventricular end disatolic volume; RVESV, right ventricular end systolic volume; RVSV, right ventricular stroke volume


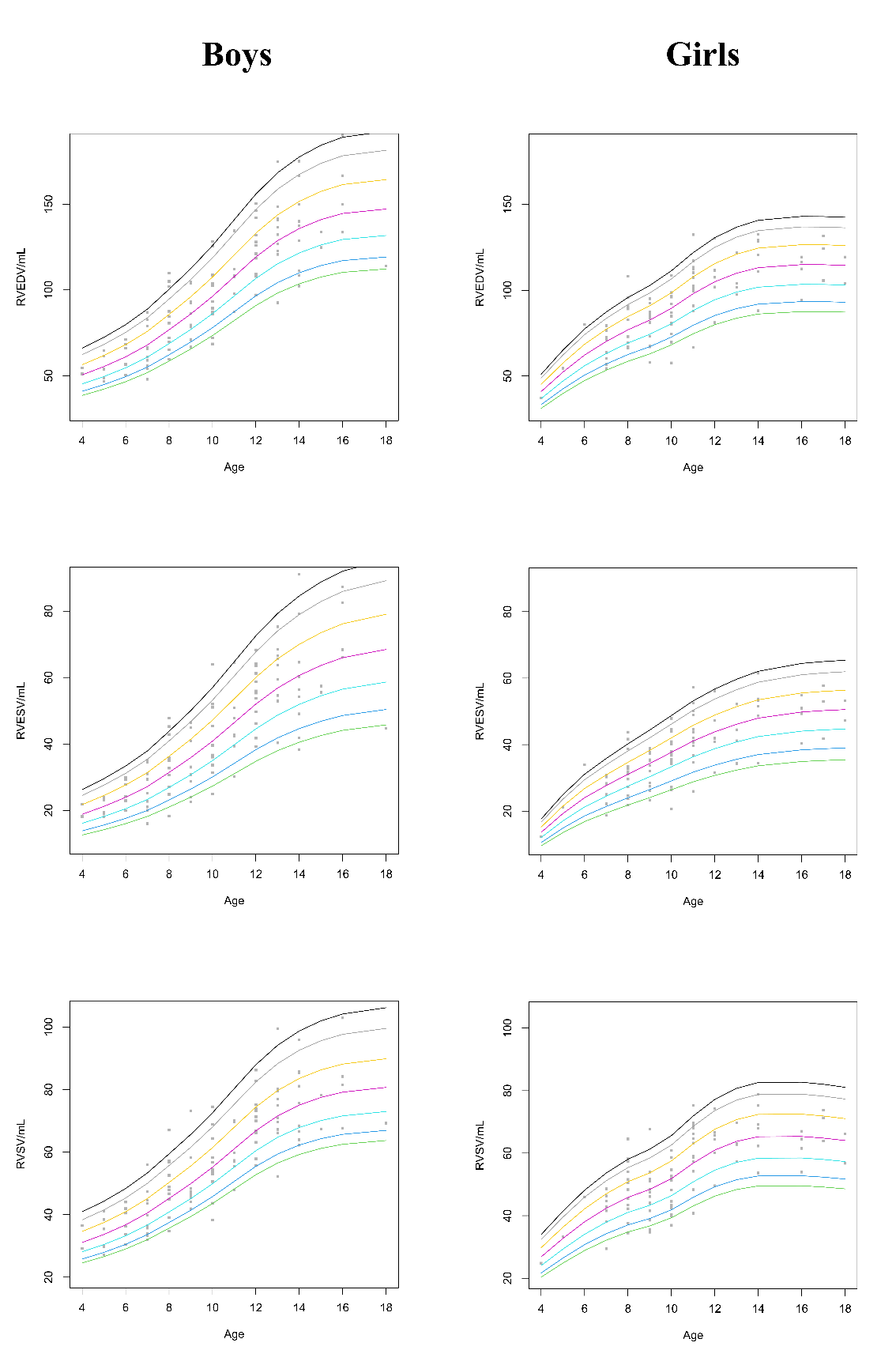


**Supplementary Figure S5.** **Reference percentiles curves for LVEDV, LVESV, LVSV, and LVM by BSA for boys (left column) and girls (right column) with exclusion of trabeculations and papillary muscles in the volume.** Abbreviations as in Supplementary Figure S3.


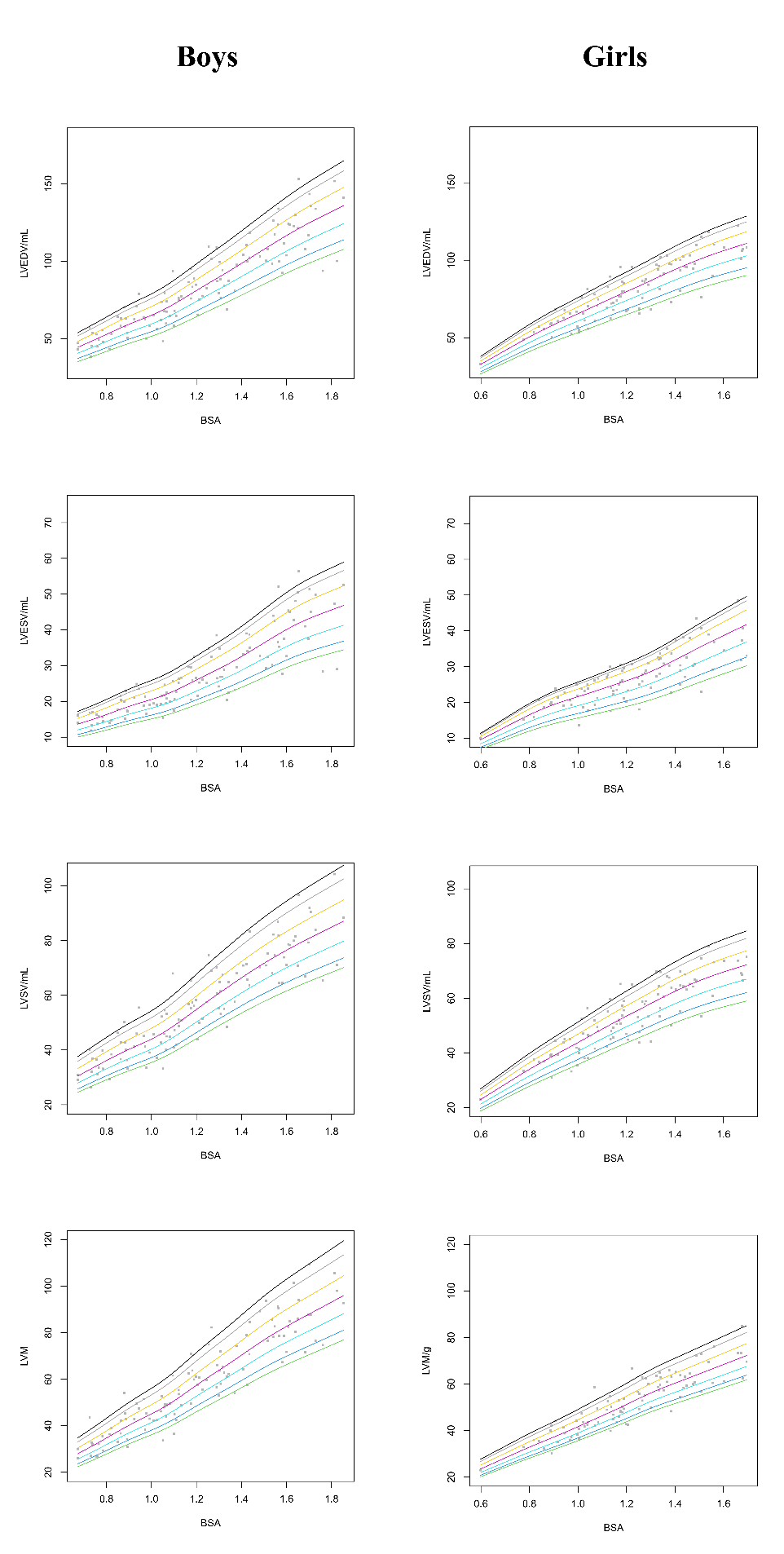


**Supplementary Figure S6.** **Reference percentiles curves for LVEDV, LVESV, LVSV, and LVM by age for boys (left column) and girls (right column) with exclusion of trabeculations and papillary muscles in the volume.** Abbreviations as in Supplementary Figure S3.


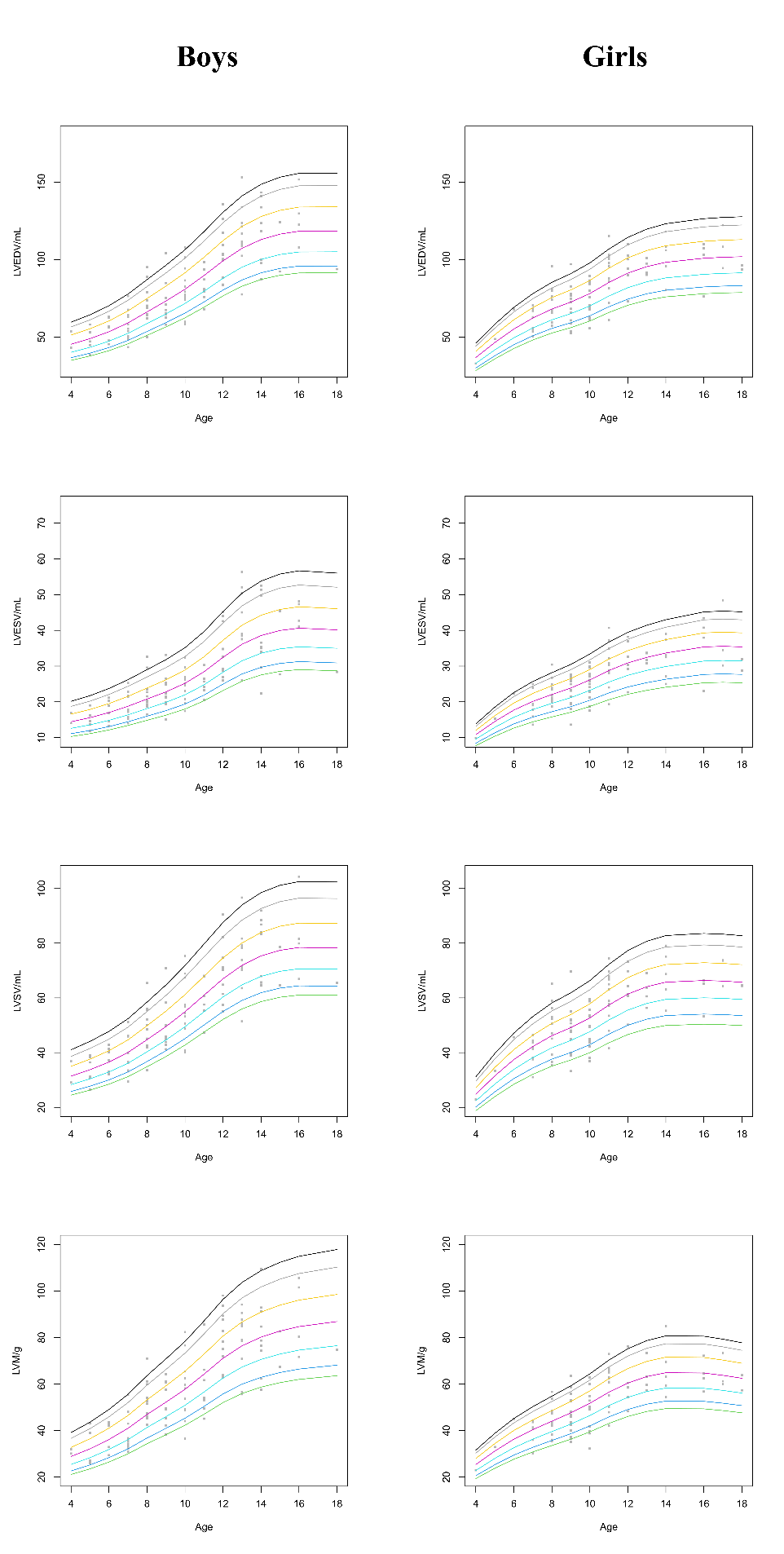


**Supplementary Figure S7.** **Reference percentiles curves for RVEDV, RVESV, and RVSV by BSA for boys (left column) and girls (right column) with exclusion of trabeculations and papillary muscles in the volume.** Abbreviations as in Supplementary Figure S4.


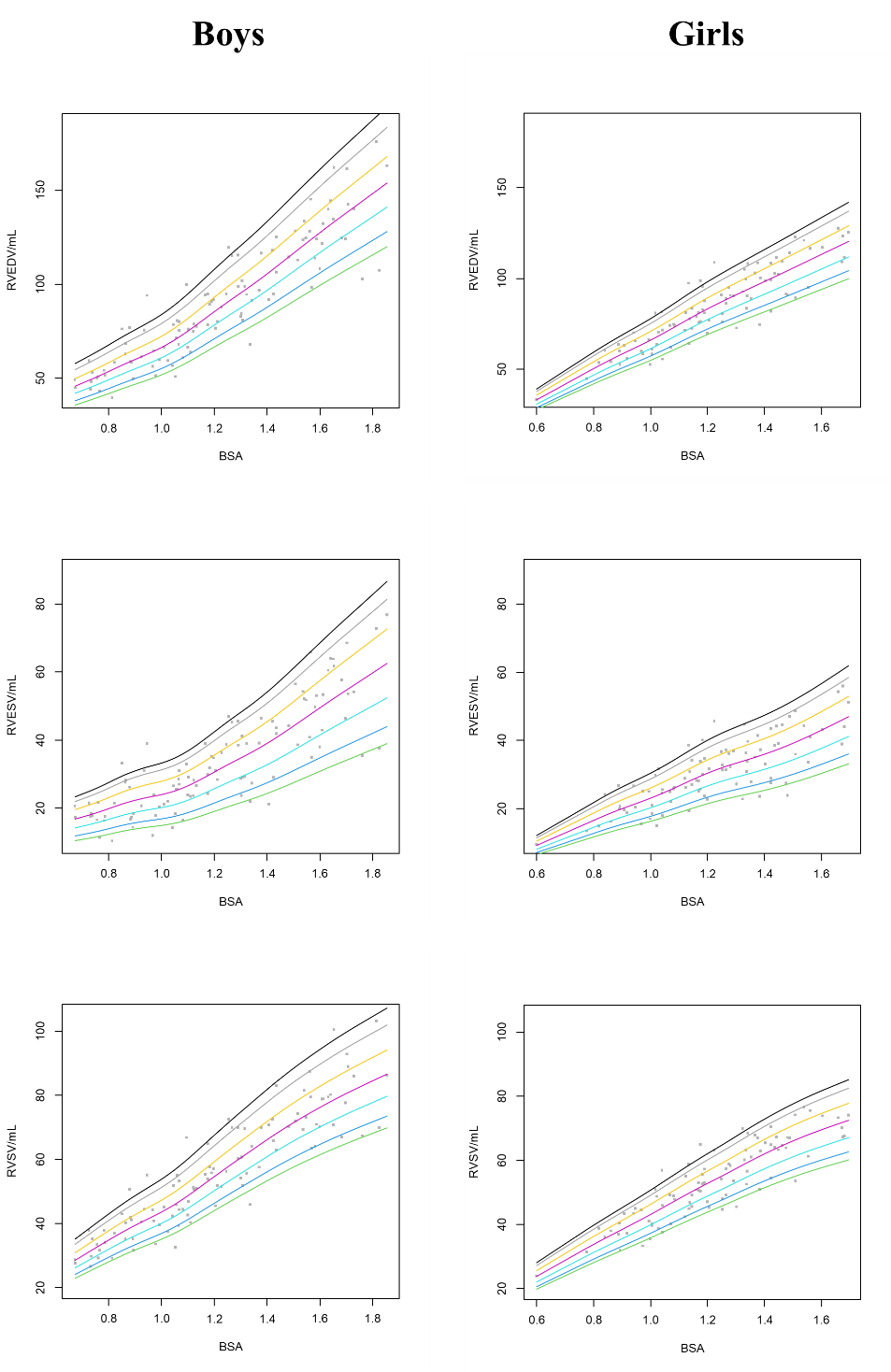


**Supplementary Figure S8.** **Reference percentiles curves for RVEDV, RVESV, and RVSV by age for boys (left column) and girls (right column) with exclusion of trabeculations and papillary muscles in the volume.** Abbreviations as in Supplementary Figure S4.


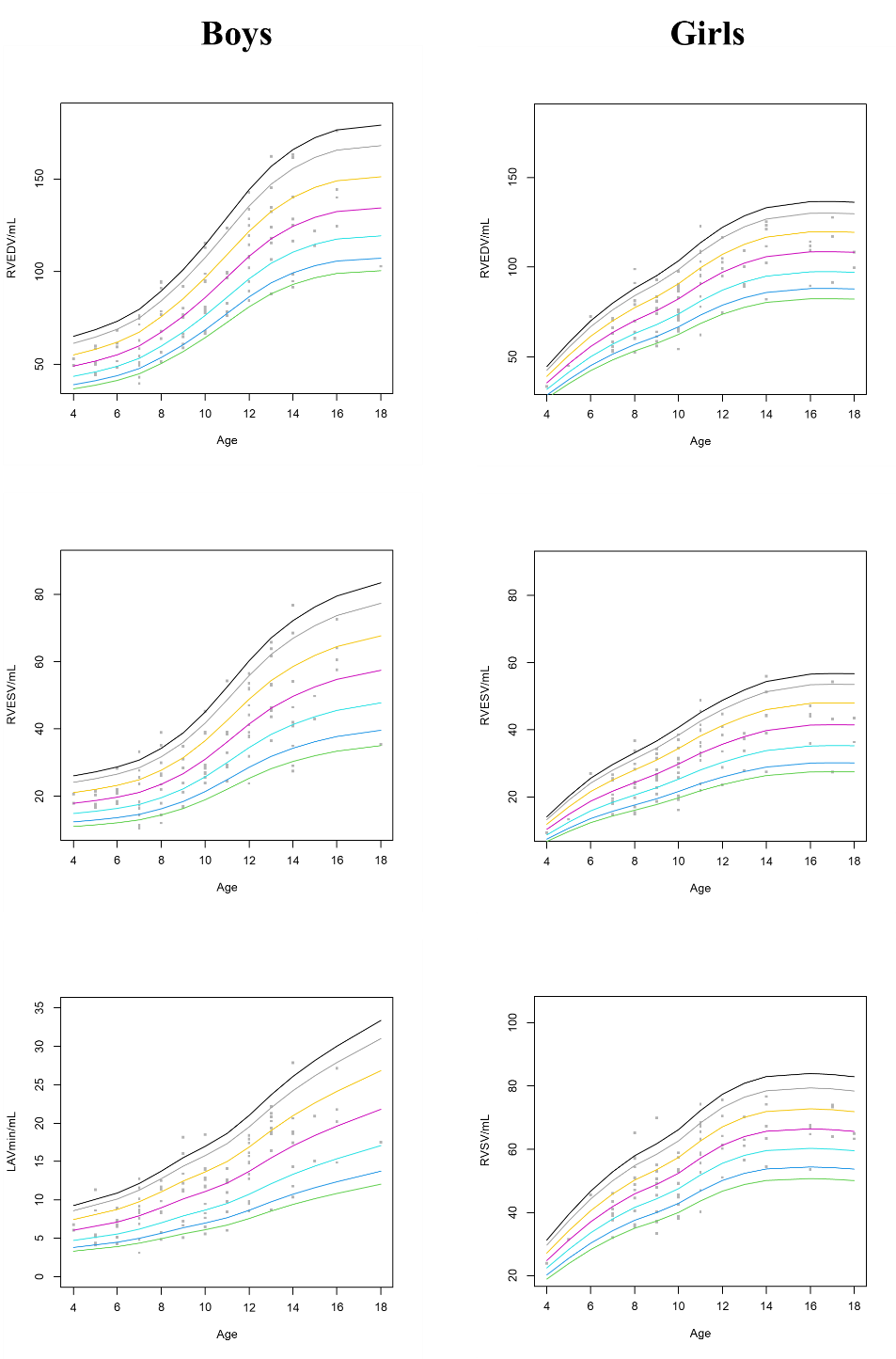


**Supplementary Figure S9.** **Reference percentiles curves for LAV_min,_ LAV_max_, RAV_min_, and RAV_max_ by age for boys (left column) and girls (right column).** LAV_min_: Minimal left atrial volume; LAV_max_: Maximal left atrial volume; RAV_min_: Minimal right atrial volume; RAV_max_: Maximal right atrial volume.


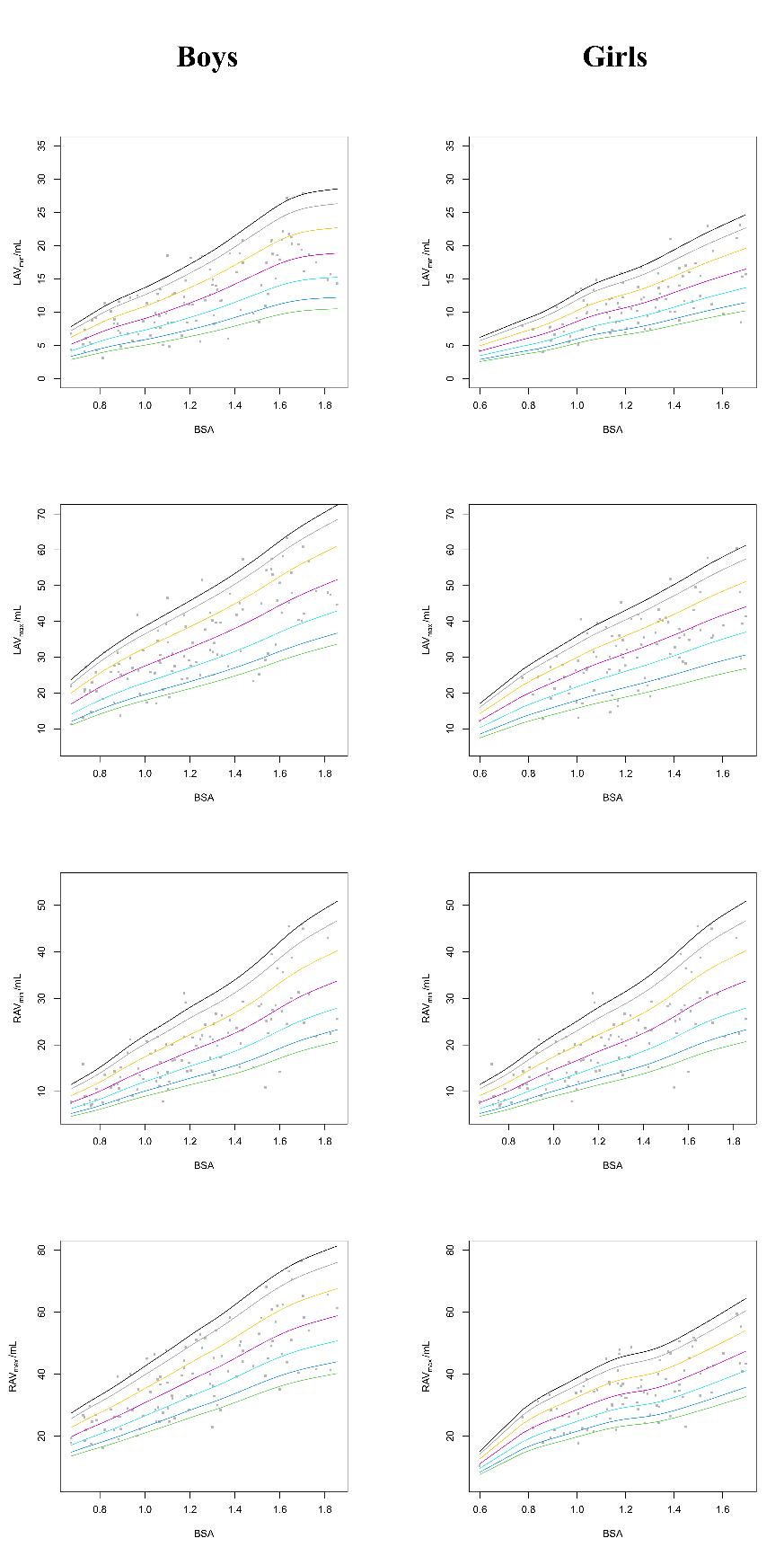


**Supplementary Figure S10.** **Forest plots of ventricular parameters indexed to BSA.** LVEDVi: Left ventricular end-diastolic volume index; LVESVi: Left ventricular end-systolic volume index; LVMi: Left ventricular mass index; LVSVi: Left ventricular stroke volume index; RVEDVi: Right ventricular end-diastolic volume index; RVESVi: Right ventricular end-systolic volume index; RVSVi: Right ventricular stroke volume index;


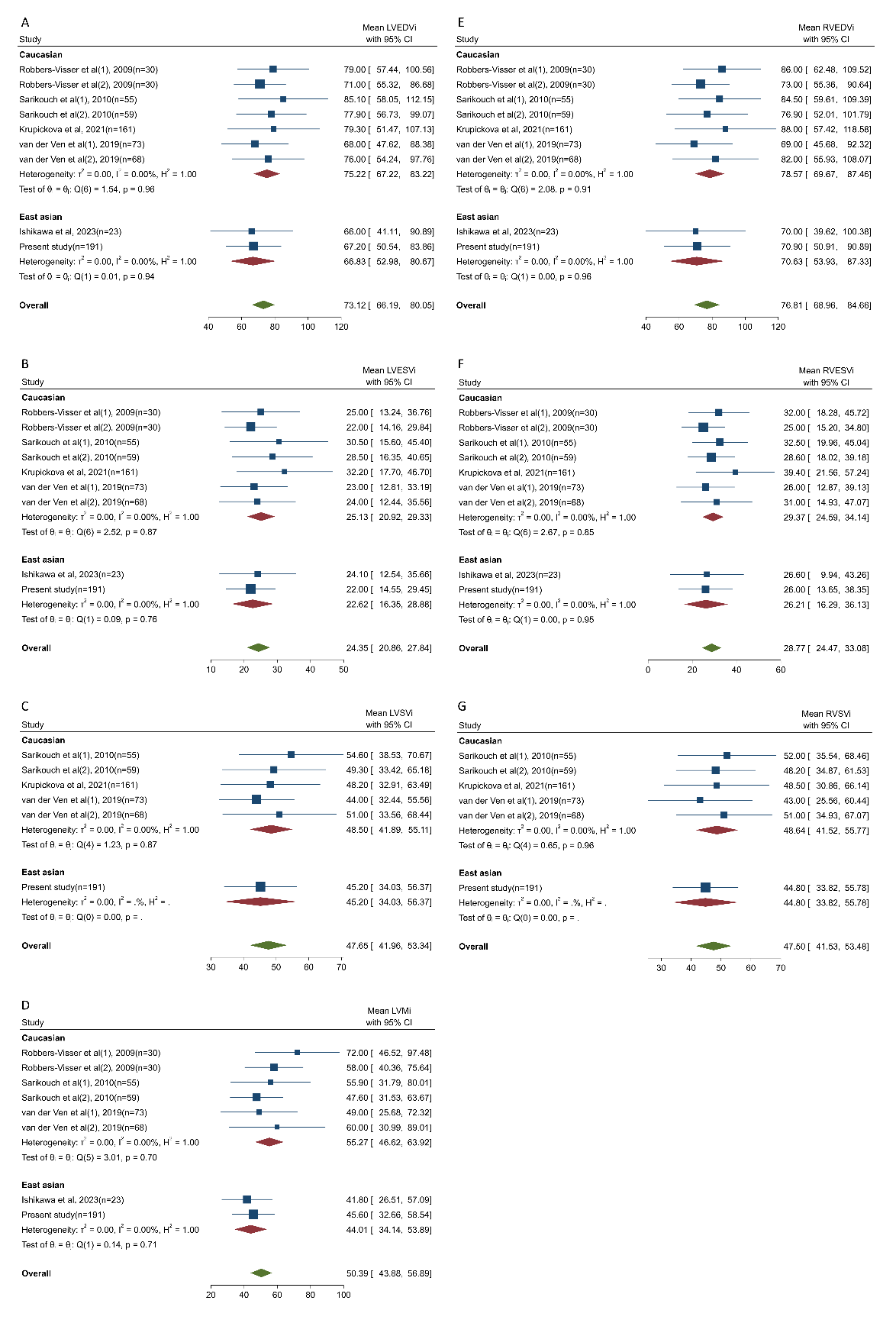


**Supplementary Figure S11.** **Forest plots of atrial parameters indexed to BSA.** iLAV_min_ : Minimal left atrial volume index; iLAV_max_ : Maximal left atrial volume index; iRAV_min_ : Minimal right atrial volume index; iRAV_max_ : Maximal right atrial volume index.


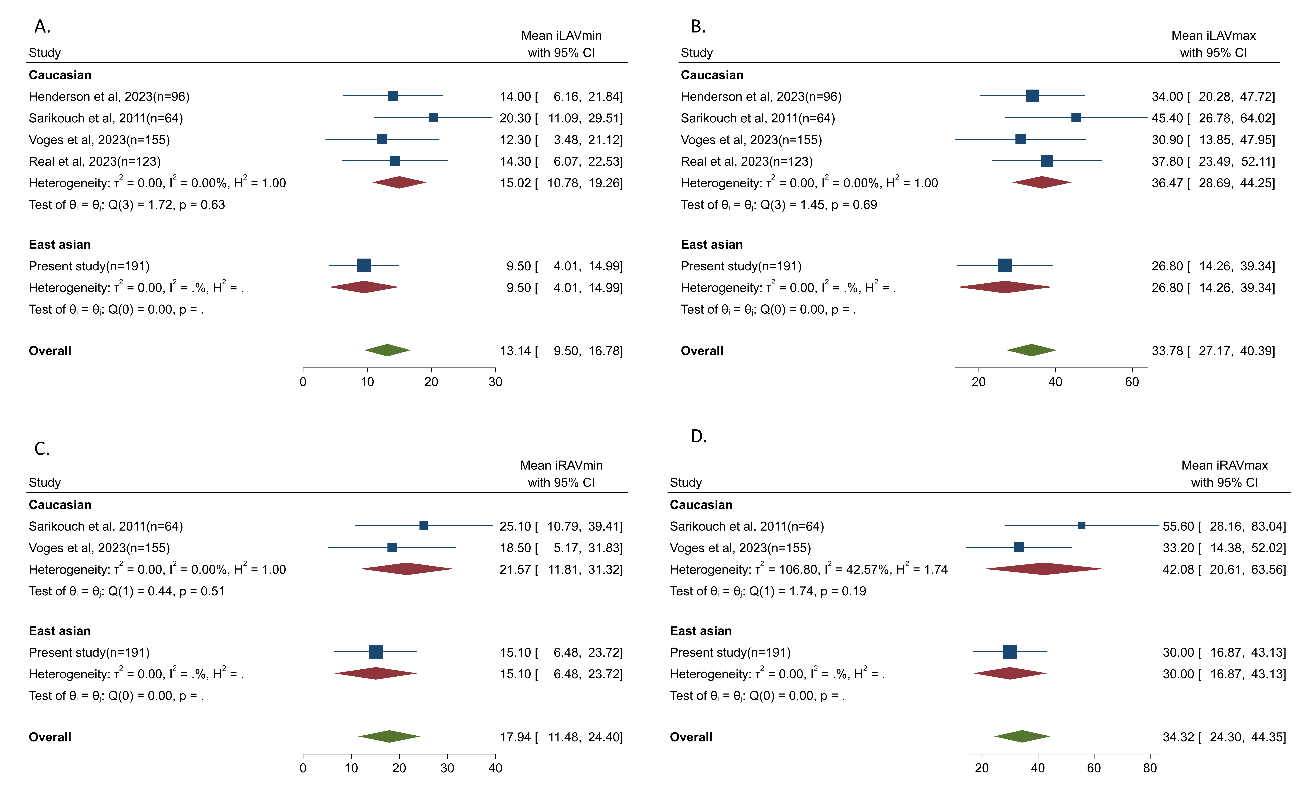

Supplement: Supplementary file 1 — Supplementary material [file mmc1.docx]
