# Supplementary material for: Age-, body surface area-, and sex-specific reference values for cardiovascular magnetic resonance imaging derived ventricular and atrial size and function for Chinese healthy children
Source: J Cardiovasc Magn Reson. 2025 Mar 21;27(1):101885. doi: 10.1016/j.jocmr.2025.101885 (PMC12182818; doi:10.1016/j.jocmr.2025.101885)
Supplement: Supplementary file 2 — Supplementary material [file mmc2.docx]

**Supplementary Tables**

**Supplementary Table S1. Comparison of different countouring methods for ventricular size and function**

|  | Smooth Segmentation | Anatomic Segmentation | P |
| --- | --- | --- | --- |
| LVEDV (mL) | 91.3±26.0 | 82.6±24.0 | <0.001 |
| LVESV(mL) | 35.7±11.2 | 27.2±9.2 | <0.001 |
| LVSV(mL) | 55.8±15.3 | 55.4±15.4 | <0.001 |
| LVEF(%) | 61.4±2.7 | 67.4±3.1 | <0.001 |
| LVM(g) | 47.2±15.3 | 55.2±17.5 | <0.001 |
| RVEDV(mL) | 95.9±29.0 | 87.5±27.4 | <0.001 |
| RVESV(mL) | 40.4±14.8 | 32.5±13.0 | <0.001 |
| RVSV(mL) | 55.4±15.2 | 55.1±15.5 | <0.001 |
| RVEF(%) | 58.5±4.5 | 63.6±5.1 | <0.001 |

LVEDV: Left ventricular end-diastolic volume; LVESV: Left ventricular end-systolic volume; LVM: Left ventricular mass; LVSV: Left ventricular stroke volume; LVEF: Left ventricular ejection fraction; RVEDV: Right ventricular end-diastolic volume; RVEF: Right ventricular ejection fraction; RVESV: Right ventricular end-systolic volume; RVSV: Right ventricular stroke volume.

**Supplementary Table S2. Centiles of RVEDV with inclusion of trabeculations and papillary muscles in ventricular volume by BSA and sex.**

| BSA | Boys | | | | | | |  | Girls | | | | | | |
| --- | --- | --- | --- | --- | --- | --- | --- | --- | --- | --- | --- | --- | --- | --- | --- |
|  | 5th | 10th | 25th | 50th | 75th | 90th | 95th |  | 5th | 10th | 25th | 50th | 75th | 90th | 95th |
| 0.6 | 34.8 | 36.8 | 40.1 | 43.5 | 47.2 | 51.5 | 54.5 |  | 31.3 | 32.6 | 34.9 | 37.6 | 40.3 | 42.9 | 44.5 |
| 0.7 | 40.9 | 43.2 | 47.0 | 51.0 | 55.4 | 60.4 | 63.9 |  | 39.2 | 40.8 | 43.7 | 47.0 | 50.5 | 53.8 | 55.8 |
| 0.8 | 47.2 | 49.9 | 54.3 | 58.9 | 64.0 | 69.8 | 73.9 |  | 46.8 | 48.8 | 52.2 | 56.1 | 60.3 | 64.2 | 66.6 |
| 0.9 | 53.8 | 56.8 | 61.8 | 67.1 | 72.9 | 79.5 | 84.2 |  | 53.9 | 56.2 | 60.1 | 64.7 | 69.5 | 73.9 | 76.7 |
| 1.0 | 60.2 | 63.6 | 69.2 | 75.1 | 81.6 | 89.0 | 94.2 |  | 60.8 | 63.4 | 67.8 | 73.0 | 78.4 | 83.4 | 86.6 |
| 1.1 | 68.2 | 72.1 | 78.4 | 85.1 | 92.4 | 100.8 | 106.7 |  | 68.2 | 71.1 | 76.1 | 81.8 | 87.9 | 93.6 | 97.1 |
| 1.2 | 77.2 | 81.5 | 88.7 | 96.3 | 104.6 | 114.0 | 120.8 |  | 75.1 | 78.3 | 83.8 | 90.2 | 96.9 | 103.1 | 107.0 |
| 1.3 | 85.7 | 90.6 | 98.6 | 107.0 | 116.2 | 126.7 | 134.2 |  | 81.4 | 84.9 | 90.9 | 97.8 | 105.0 | 111.8 | 115.9 |
| 1.4 | 94.1 | 99.5 | 108.2 | 117.5 | 127.6 | 139.1 | 147.3 |  | 87.8 | 91.6 | 98.0 | 105.4 | 113.2 | 120.5 | 125.0 |
| 1.5 | 102.7 | 108.5 | 118.1 | 128.2 | 139.2 | 151.8 | 160.7 |  | 94.4 | 98.3 | 105.2 | 113.3 | 121.6 | 129.5 | 134.3 |
| 1.6 | 110.8 | 117.1 | 127.4 | 138.2 | 150.1 | 163.7 | 173.4 |  | 100.6 | 104.9 | 112.3 | 120.8 | 129.7 | 138.1 | 143.3 |
| 1.7 | 118.2 | 124.9 | 135.9 | 147.5 | 160.2 | 174.7 | 185.0 |  | 106.7 | 111.2 | 119.0 | 128.0 | 137.5 | 146.3 | 151.8 |
| 1.8 | 125.2 | 132.3 | 144.0 | 156.3 | 169.7 | 185.1 | 196.0 |  | 112.6 | 117.4 | 125.6 | 135.2 | 145.2 | 154.5 | 160.3 |
| 1.9 | 132.3 | 139.8 | 152.1 | 165.1 | 179.3 | 195.5 | 207.1 |  | 118.6 | 123.6 | 132.3 | 142.3 | 152.9 | 162.7 | 168.8 |

BSA: Body surface area; RVEDV: Right ventricular end-diastolic volume

**Supplementary Table S3. Centiles of RVESV with inclusion of trabeculations and papillary muscles in ventricular volume by BSA and sex.**

| BSA | Boys | | | | | | |  | Girls | | | | | | |
| --- | --- | --- | --- | --- | --- | --- | --- | --- | --- | --- | --- | --- | --- | --- | --- |
|  | 5th | 10th | 25th | 50th | 75th | 90th | 95th |  | 5th | 10th | 25th | 50th | 75th | 90th | 95th |
| 0.6 | 11.9 | 12.8 | 14.6 | 16.9 | 19.4 | 21.8 | 23.2 |  | 9.0 | 9.7 | 10.9 | 12.2 | 13.5 | 14.6 | 15.3 |
| 0.7 | 14.1 | 15.2 | 17.3 | 20.0 | 23.1 | 25.8 | 27.5 |  | 12.5 | 13.5 | 15.2 | 17.0 | 18.7 | 20.3 | 21.3 |
| 0.8 | 16.5 | 17.8 | 20.2 | 23.4 | 27.0 | 30.2 | 32.1 |  | 16.0 | 17.2 | 19.3 | 21.6 | 23.9 | 25.9 | 27.1 |
| 0.9 | 19.2 | 20.7 | 23.6 | 27.4 | 31.5 | 35.2 | 37.5 |  | 19.2 | 20.7 | 23.3 | 26.0 | 28.7 | 31.2 | 32.6 |
| 1.0 | 21.7 | 23.4 | 26.6 | 30.8 | 35.5 | 39.7 | 42.3 |  | 22.3 | 24.1 | 27.0 | 30.2 | 33.4 | 36.2 | 37.9 |
| 1.1 | 24.6 | 26.5 | 30.2 | 35.0 | 40.3 | 45.1 | 48.0 |  | 25.5 | 27.5 | 30.8 | 34.5 | 38.1 | 41.3 | 43.2 |
| 1.2 | 28.3 | 30.5 | 34.7 | 40.2 | 46.2 | 51.7 | 55.1 |  | 28.1 | 30.4 | 34.0 | 38.1 | 42.1 | 45.6 | 47.7 |
| 1.3 | 31.7 | 34.2 | 38.9 | 45.1 | 51.9 | 58.1 | 61.8 |  | 30.1 | 32.5 | 36.4 | 40.8 | 45.0 | 48.8 | 51.1 |
| 1.4 | 35.0 | 37.7 | 42.9 | 49.7 | 57.2 | 64.1 | 68.2 |  | 32.3 | 34.8 | 39.0 | 43.6 | 48.2 | 52.3 | 54.7 |
| 1.5 | 38.5 | 41.6 | 47.3 | 54.8 | 63.1 | 70.6 | 75.1 |  | 34.9 | 37.6 | 42.2 | 47.2 | 52.1 | 56.5 | 59.1 |
| 1.6 | 42.1 | 45.4 | 51.7 | 59.9 | 68.9 | 77.2 | 82.1 |  | 37.6 | 40.6 | 45.5 | 50.9 | 56.2 | 61.0 | 63.8 |
| 1.7 | 45.5 | 49.1 | 55.9 | 64.7 | 74.5 | 83.4 | 88.7 |  | 40.4 | 43.6 | 48.8 | 54.6 | 60.3 | 65.4 | 68.5 |
| 1.8 | 48.8 | 52.6 | 59.9 | 69.4 | 79.9 | 89.4 | 95.1 |  | 43.1 | 46.5 | 52.1 | 58.3 | 64.4 | 69.9 | 73.1 |
| 1.9 | 52.2 | 56.3 | 64.1 | 74.2 | 85.4 | 95.6 | 101.7 |  | 45.8 | 49.5 | 55.4 | 62.0 | 68.5 | 74.3 | 77.7 |

BSA: Body surface area; RVESV: Right ventricular end-systolic volume

**Supplementary Table S4. Centiles of RVSV with inclusion of trabeculations and papillary muscles in ventricular volume by BSA and sex.**

| BSA | Boys | | | | | | |  | Girls | | | | | | |
| --- | --- | --- | --- | --- | --- | --- | --- | --- | --- | --- | --- | --- | --- | --- | --- |
|  | 5th | 10th | 25th | 50th | 75th | 90th | 95th |  | 5th | 10th | 25th | 50th | 75th | 90th | 95th |
| 0.6 | 21.3 | 22.3 | 24.1 | 26.2 | 28.5 | 30.7 | 32.1 |  | 20.2 | 21.2 | 22.9 | 24.7 | 26.6 | 28.2 | 29.2 |
| 0.7 | 25.4 | 26.6 | 28.8 | 31.3 | 34.0 | 36.7 | 38.3 |  | 24.2 | 25.4 | 27.4 | 29.6 | 31.9 | 33.8 | 35.0 |
| 0.8 | 29.5 | 30.9 | 33.4 | 36.3 | 39.4 | 42.5 | 44.4 |  | 28.1 | 29.5 | 31.8 | 34.4 | 37.0 | 39.3 | 40.6 |
| 0.9 | 33.2 | 34.8 | 37.6 | 40.9 | 44.4 | 47.9 | 50.1 |  | 31.8 | 33.3 | 35.9 | 38.9 | 41.8 | 44.4 | 45.9 |
| 1.0 | 36.5 | 38.2 | 41.3 | 44.9 | 48.8 | 52.6 | 55.0 |  | 35.5 | 37.2 | 40.1 | 43.4 | 46.7 | 49.6 | 51.2 |
| 1.1 | 40.4 | 42.3 | 45.7 | 49.7 | 54.1 | 58.3 | 60.9 |  | 39.3 | 41.2 | 44.5 | 48.1 | 51.8 | 55.0 | 56.8 |
| 1.2 | 44.9 | 47.1 | 50.9 | 55.3 | 60.1 | 64.8 | 67.8 |  | 43.2 | 45.3 | 48.8 | 52.8 | 56.8 | 60.3 | 62.4 |
| 1.3 | 49.5 | 51.9 | 56.0 | 60.9 | 66.2 | 71.4 | 74.6 |  | 46.9 | 49.2 | 53.1 | 57.5 | 61.8 | 65.6 | 67.8 |
| 1.4 | 54.1 | 56.7 | 61.2 | 66.6 | 72.4 | 78.0 | 81.6 |  | 50.6 | 53.1 | 57.3 | 62.0 | 66.7 | 70.8 | 73.2 |
| 1.5 | 58.5 | 61.3 | 66.2 | 72.0 | 78.2 | 84.3 | 88.2 |  | 53.9 | 56.5 | 60.9 | 66.0 | 70.9 | 75.3 | 77.9 |
| 1.6 | 62.3 | 65.3 | 70.5 | 76.7 | 83.4 | 89.8 | 93.9 |  | 56.6 | 59.4 | 64.1 | 69.3 | 74.6 | 79.1 | 81.8 |
| 1.7 | 65.4 | 68.6 | 74.1 | 80.6 | 87.6 | 94.4 | 98.7 |  | 59.0 | 61.9 | 66.8 | 72.3 | 77.8 | 82.5 | 85.3 |
| 1.8 | 68.0 | 71.2 | 76.9 | 83.7 | 91.0 | 98.1 | 102.5 |  | 61.4 | 64.4 | 69.5 | 75.2 | 80.9 | 85.9 | 88.8 |
| 1.9 | 70.3 | 73.7 | 79.6 | 86.6 | 94.1 | 101.4 | 106.0 |  | 63.8 | 67.0 | 72.2 | 78.2 | 84.1 | 89.2 | 92.3 |

BSA: Body surface area; RVSV: Right ventricular stroke volume

**Supplementary Table S5. Centiles of LVEDV with inclusion of trabeculations and papillary muscles in volume by age and sex.**

| Age | Boys | | | | | | |  | Girls | | | | | | |
| --- | --- | --- | --- | --- | --- | --- | --- | --- | --- | --- | --- | --- | --- | --- | --- |
|  | 5th | 10th | 25th | 50th | 75th | 90th | 95th |  | 5th | 10th | 25th | 50th | 75th | 90th | 95th |
| 4.0 | 37.9 | 39.8 | 43.5 | 48.9 | 55.0 | 60.5 | 63.8 |  | 32.9 | 35.0 | 38.7 | 42.9 | 47.3 | 51.2 | 53.6 |
| 5.0 | 41.7 | 43.8 | 48.0 | 53.8 | 60.6 | 66.7 | 70.3 |  | 40.3 | 42.9 | 47.4 | 52.6 | 58.0 | 62.8 | 65.6 |
| 6.0 | 46.3 | 48.7 | 53.3 | 59.8 | 67.2 | 74.0 | 78.1 |  | 47.0 | 50.0 | 55.3 | 61.4 | 67.6 | 73.2 | 76.6 |
| 7.0 | 51.7 | 54.3 | 59.4 | 66.7 | 75.0 | 82.6 | 87.1 |  | 52.7 | 56.1 | 62.0 | 68.9 | 75.8 | 82.1 | 85.9 |
| 8.0 | 57.7 | 60.6 | 66.3 | 74.4 | 83.7 | 92.2 | 97.2 |  | 57.7 | 61.5 | 67.9 | 75.4 | 83.0 | 89.9 | 94.0 |
| 9.0 | 63.4 | 66.6 | 72.9 | 81.8 | 92.0 | 101.3 | 106.8 |  | 61.5 | 65.5 | 72.4 | 80.4 | 88.5 | 95.8 | 100.3 |
| 10.0 | 69.7 | 73.2 | 80.1 | 89.9 | 101.1 | 111.3 | 117.4 |  | 66.1 | 70.5 | 77.9 | 86.4 | 95.2 | 103.0 | 107.8 |
| 11.0 | 77.3 | 81.1 | 88.8 | 99.7 | 112.2 | 123.4 | 130.2 |  | 72.2 | 76.9 | 85.0 | 94.3 | 103.9 | 112.5 | 117.7 |
| 12.0 | 85.3 | 89.6 | 98.1 | 110.1 | 123.8 | 136.3 | 143.8 |  | 77.3 | 82.3 | 91.0 | 101.0 | 111.2 | 120.4 | 125.9 |
| 13.0 | 92.0 | 96.6 | 105.8 | 118.7 | 133.6 | 147.0 | 155.1 |  | 80.7 | 85.9 | 95.0 | 105.4 | 116.1 | 125.7 | 131.5 |
| 14.0 | 96.8 | 101.6 | 111.3 | 124.9 | 140.5 | 154.6 | 163.1 |  | 82.8 | 88.2 | 97.5 | 108.2 | 119.1 | 129.0 | 134.9 |
| 15.0 | 99.9 | 105.0 | 114.9 | 129.0 | 145.1 | 159.7 | 168.4 |  | 83.8 | 89.3 | 98.7 | 109.5 | 120.6 | 130.6 | 136.6 |
| 16.0 | 101.9 | 107.0 | 117.1 | 131.4 | 147.9 | 162.7 | 171.7 |  | 84.1 | 89.6 | 99.1 | 110.0 | 121.1 | 131.1 | 137.1 |
| 17.0 | 102.6 | 107.8 | 118.0 | 132.4 | 149.0 | 164.0 | 172.9 |  | 84.1 | 89.6 | 99.0 | 109.9 | 121.0 | 131.0 | 137.1 |
| 18.0 | 102.8 | 107.9 | 118.1 | 132.6 | 149.2 | 164.2 | 173.2 |  | 83.8 | 89.2 | 98.6 | 109.4 | 120.5 | 130.5 | 136.5 |

BSA: Body surface area; LVEDV: Left ventricular end-diastolic volume

**Supplementary Table S6. Centiles of LVESV with inclusion of trabeculations and papillary muscles in volume by age and sex.**

| Age | Boys | | | | | | |  | Girls | | | | | | |
| --- | --- | --- | --- | --- | --- | --- | --- | --- | --- | --- | --- | --- | --- | --- | --- |
|  | 5th | 10th | 25th | 50th | 75th | 90th | 95th |  | 5th | 10th | 25th | 50th | 75th | 90th | 95th |
| 4.0 | 13.1 | 14.1 | 15.9 | 18.1 | 20.5 | 23.0 | 24.5 |  | 11.7 | 12.5 | 14.0 | 15.7 | 17.5 | 19.1 | 20.0 |
| 5.0 | 14.6 | 15.7 | 17.7 | 20.2 | 23.0 | 25.7 | 27.4 |  | 14.5 | 15.6 | 17.4 | 19.6 | 21.9 | 23.8 | 25.0 |
| 6.0 | 16.5 | 17.7 | 20.0 | 22.8 | 25.9 | 28.9 | 30.9 |  | 17.1 | 18.3 | 20.5 | 23.0 | 25.7 | 28.0 | 29.3 |
| 7.0 | 18.4 | 19.8 | 22.4 | 25.5 | 29.0 | 32.4 | 34.6 |  | 19.3 | 20.7 | 23.1 | 26.0 | 29.0 | 31.6 | 33.1 |
| 8.0 | 20.5 | 22.0 | 24.9 | 28.3 | 32.2 | 35.9 | 38.4 |  | 21.3 | 22.9 | 25.6 | 28.8 | 32.1 | 34.9 | 36.6 |
| 9.0 | 22.3 | 24.0 | 27.1 | 30.8 | 35.0 | 39.1 | 41.8 |  | 22.9 | 24.6 | 27.5 | 31.0 | 34.5 | 37.6 | 39.4 |
| 10.0 | 24.5 | 26.4 | 29.8 | 33.9 | 38.5 | 43.0 | 46.0 |  | 24.9 | 26.7 | 29.8 | 33.6 | 37.4 | 40.8 | 42.7 |
| 11.0 | 27.5 | 29.6 | 33.4 | 38.1 | 43.2 | 48.3 | 51.6 |  | 27.2 | 29.2 | 32.7 | 36.8 | 41.0 | 44.6 | 46.8 |
| 12.0 | 31.0 | 33.4 | 37.7 | 42.9 | 48.7 | 54.5 | 58.1 |  | 29.2 | 31.3 | 35.0 | 39.4 | 43.9 | 47.8 | 50.1 |
| 13.0 | 34.0 | 36.6 | 41.3 | 47.1 | 53.5 | 59.8 | 63.8 |  | 30.5 | 32.8 | 36.6 | 41.3 | 46.0 | 50.1 | 52.5 |
| 14.0 | 36.0 | 38.7 | 43.7 | 49.8 | 56.6 | 63.2 | 67.5 |  | 31.5 | 33.8 | 37.8 | 42.6 | 47.4 | 51.7 | 54.2 |
| 15.0 | 37.1 | 40.0 | 45.1 | 51.4 | 58.3 | 65.2 | 69.6 |  | 32.2 | 34.6 | 38.7 | 43.5 | 48.5 | 52.8 | 55.4 |
| 16.0 | 37.6 | 40.4 | 45.6 | 52.0 | 59.0 | 66.0 | 70.4 |  | 32.7 | 35.1 | 39.3 | 44.2 | 49.3 | 53.7 | 56.3 |
| 17.0 | 37.4 | 40.2 | 45.4 | 51.7 | 58.8 | 65.7 | 70.1 |  | 33.1 | 35.5 | 39.7 | 44.7 | 49.8 | 54.2 | 56.8 |
| 18.0 | 36.9 | 39.7 | 44.8 | 51.0 | 57.9 | 64.8 | 69.1 |  | 33.3 | 35.7 | 39.9 | 44.9 | 50.1 | 54.5 | 57.1 |

BSA: Body surface area; LVESV: Left ventricular end-systolic volume

**Supplementary Table S7. Centiles of LVSV with inclusion of trabeculations and papillary muscles in volume by age and sex.**

| Age | Boys | | | | | | |  | Girls | | | | | | |
| --- | --- | --- | --- | --- | --- | --- | --- | --- | --- | --- | --- | --- | --- | --- | --- |
|  | 5th | 10th | 25th | 50th | 75th | 90th | 95th |  | 5th | 10th | 25th | 50th | 75th | 90th | 95th |
| 4.0 | 24.7 | 26.0 | 28.3 | 31.3 | 34.8 | 38.4 | 40.8 |  | 20.5 | 22.0 | 24.4 | 27.0 | 29.7 | 32.2 | 33.8 |
| 5.0 | 26.7 | 28.1 | 30.6 | 33.9 | 37.6 | 41.5 | 44.1 |  | 25.0 | 26.8 | 29.7 | 32.9 | 36.2 | 39.3 | 41.2 |
| 6.0 | 29.1 | 30.6 | 33.4 | 36.9 | 41.0 | 45.3 | 48.1 |  | 29.1 | 31.2 | 34.6 | 38.3 | 42.1 | 45.7 | 47.9 |
| 7.0 | 32.1 | 33.8 | 36.8 | 40.7 | 45.2 | 49.9 | 53.1 |  | 32.5 | 34.9 | 38.7 | 42.9 | 47.1 | 51.1 | 53.6 |
| 8.0 | 35.8 | 37.7 | 41.1 | 45.5 | 50.5 | 55.7 | 59.2 |  | 35.4 | 37.9 | 42.1 | 46.7 | 51.3 | 55.6 | 58.3 |
| 9.0 | 39.6 | 41.6 | 45.4 | 50.2 | 55.8 | 61.5 | 65.4 |  | 37.6 | 40.2 | 44.6 | 49.5 | 54.4 | 59.0 | 61.9 |
| 10.0 | 43.7 | 46.0 | 50.2 | 55.5 | 61.6 | 68.0 | 72.3 |  | 40.2 | 43.1 | 47.8 | 52.9 | 58.2 | 63.1 | 66.2 |
| 11.0 | 48.4 | 50.9 | 55.6 | 61.4 | 68.2 | 75.3 | 80.1 |  | 43.8 | 46.9 | 52.0 | 57.7 | 63.4 | 68.8 | 72.1 |
| 12.0 | 53.1 | 55.9 | 61.0 | 67.4 | 74.9 | 82.7 | 87.9 |  | 46.8 | 50.1 | 55.6 | 61.6 | 67.7 | 73.4 | 77.0 |
| 13.0 | 57.0 | 60.0 | 65.4 | 72.3 | 80.4 | 88.7 | 94.3 |  | 48.7 | 52.2 | 57.9 | 64.1 | 70.5 | 76.5 | 80.2 |
| 14.0 | 59.8 | 62.9 | 68.7 | 75.9 | 84.3 | 93.1 | 98.9 |  | 49.8 | 53.3 | 59.2 | 65.5 | 72.0 | 78.2 | 82.0 |
| 15.0 | 61.8 | 65.0 | 70.9 | 78.4 | 87.1 | 96.1 | 102.2 |  | 50.0 | 53.6 | 59.5 | 65.9 | 72.4 | 78.6 | 82.4 |
| 16.0 | 63.1 | 66.3 | 72.4 | 80.0 | 88.9 | 98.1 | 104.3 |  | 49.8 | 53.4 | 59.2 | 65.6 | 72.1 | 78.3 | 82.1 |
| 17.0 | 63.8 | 67.1 | 73.3 | 81.0 | 90.0 | 99.3 | 105.6 |  | 49.4 | 52.9 | 58.8 | 65.1 | 71.5 | 77.6 | 81.4 |
| 18.0 | 64.3 | 67.6 | 73.8 | 81.6 | 90.6 | 100.0 | 106.3 |  | 48.8 | 52.3 | 58.1 | 64.3 | 70.7 | 76.7 | 80.5 |

BSA: Body surface area; LVSV: Left ventricular stroke volume

**Supplementary Table S8. Centiles of LVM with inclusion of trabeculations and papillary muscles in volume by age and sex.**

| Age | Boys | | | | | | |  | Girls | | | | | | |
| --- | --- | --- | --- | --- | --- | --- | --- | --- | --- | --- | --- | --- | --- | --- | --- |
|  | 5th | 10th | 25th | 50th | 75th | 90th | 95th |  | 5th | 10th | 25th | 50th | 75th | 90th | 95th |
| 4.0 | 18.1 | 19.5 | 22.1 | 25.2 | 28.7 | 32.3 | 34.5 |  | 14.1 | 15.5 | 17.4 | 19.1 | 20.8 | 22.6 | 23.7 |
| 5.0 | 19.5 | 21.0 | 23.8 | 27.2 | 31.0 | 34.7 | 37.2 |  | 18.7 | 20.5 | 23.0 | 25.3 | 27.5 | 29.8 | 31.4 |
| 6.0 | 21.4 | 23.1 | 26.1 | 29.8 | 34.0 | 38.1 | 40.8 |  | 22.5 | 24.7 | 27.8 | 30.6 | 33.2 | 36.0 | 37.9 |
| 7.0 | 23.9 | 25.8 | 29.1 | 33.3 | 38.0 | 42.6 | 45.6 |  | 25.4 | 27.9 | 31.4 | 34.5 | 37.5 | 40.7 | 42.8 |
| 8.0 | 27.3 | 29.4 | 33.3 | 38.0 | 43.3 | 48.6 | 52.0 |  | 27.6 | 30.3 | 34.1 | 37.5 | 40.8 | 44.2 | 46.5 |
| 9.0 | 30.6 | 33.0 | 37.3 | 42.6 | 48.5 | 54.5 | 58.3 |  | 29.7 | 32.6 | 36.7 | 40.3 | 43.8 | 47.5 | 50.0 |
| 10.0 | 34.1 | 36.7 | 41.5 | 47.5 | 54.1 | 60.7 | 65.0 |  | 32.2 | 35.3 | 39.8 | 43.7 | 47.5 | 51.5 | 54.2 |
| 11.0 | 38.1 | 41.1 | 46.5 | 53.1 | 60.6 | 67.9 | 72.7 |  | 35.2 | 38.6 | 43.4 | 47.7 | 51.8 | 56.2 | 59.1 |
| 12.0 | 42.5 | 45.8 | 51.9 | 59.3 | 67.5 | 75.8 | 81.1 |  | 37.6 | 41.3 | 46.4 | 51.0 | 55.5 | 60.2 | 63.3 |
| 13.0 | 46.1 | 49.7 | 56.2 | 64.3 | 73.3 | 82.2 | 88.0 |  | 39.5 | 43.3 | 48.8 | 53.6 | 58.2 | 63.2 | 66.4 |
| 14.0 | 48.7 | 52.5 | 59.4 | 67.9 | 77.3 | 86.8 | 92.9 |  | 40.7 | 44.6 | 50.2 | 55.2 | 60.0 | 65.0 | 68.4 |
| 15.0 | 50.6 | 54.5 | 61.7 | 70.5 | 80.3 | 90.1 | 96.4 |  | 41.0 | 45.0 | 50.6 | 55.6 | 60.4 | 65.5 | 68.9 |
| 16.0 | 51.9 | 56.0 | 63.3 | 72.4 | 82.5 | 92.5 | 99.0 |  | 40.7 | 44.6 | 50.3 | 55.2 | 60.0 | 65.1 | 68.4 |
| 17.0 | 52.7 | 56.9 | 64.3 | 73.5 | 83.8 | 94.0 | 100.6 |  | 40.1 | 44.0 | 49.5 | 54.4 | 59.2 | 64.2 | 67.5 |
| 18.0 | 53.3 | 57.4 | 64.9 | 74.2 | 84.6 | 94.9 | 101.5 |  | 39.5 | 43.3 | 48.7 | 53.6 | 58.2 | 63.1 | 66.4 |

BSA: Body surface area; LVM: Left ventricular mass

**Supplementary Table S9. Centiles of RVEDV with inclusion of trabeculations and papillary muscles in volume by age and sex.**

| Age | Boys | | | | | | |  | Girls | | | | | | |
| --- | --- | --- | --- | --- | --- | --- | --- | --- | --- | --- | --- | --- | --- | --- | --- |
|  | 5th | 10th | 25th | 50th | 75th | 90th | 95th |  | 5th | 10th | 25th | 50th | 75th | 90th | 95th |
| 4.0 | 38.5 | 40.9 | 45.2 | 50.5 | 56.4 | 62.3 | 66.1 |  | 31.2 | 33.3 | 36.9 | 41.0 | 45.1 | 48.8 | 51.0 |
| 5.0 | 42.2 | 44.8 | 49.6 | 55.4 | 61.8 | 68.2 | 72.4 |  | 39.8 | 42.5 | 47.0 | 52.3 | 57.6 | 62.3 | 65.1 |
| 6.0 | 46.5 | 49.4 | 54.6 | 61.0 | 68.1 | 75.2 | 79.8 |  | 47.4 | 50.6 | 56.0 | 62.3 | 68.6 | 74.2 | 77.5 |
| 7.0 | 51.8 | 55.0 | 60.8 | 68.0 | 75.9 | 83.7 | 88.8 |  | 53.5 | 57.0 | 63.2 | 70.2 | 77.3 | 83.6 | 87.4 |
| 8.0 | 58.6 | 62.2 | 68.8 | 76.8 | 85.8 | 94.7 | 100.4 |  | 58.6 | 62.5 | 69.3 | 77.0 | 84.8 | 91.7 | 95.8 |
| 9.0 | 65.6 | 69.7 | 77.0 | 86.0 | 96.1 | 106.0 | 112.5 |  | 62.9 | 67.1 | 74.3 | 82.6 | 91.0 | 98.4 | 102.8 |
| 10.0 | 73.4 | 78.0 | 86.2 | 96.3 | 107.5 | 118.7 | 125.9 |  | 68.2 | 72.7 | 80.6 | 89.5 | 98.6 | 106.6 | 111.4 |
| 11.0 | 82.2 | 87.3 | 96.5 | 107.8 | 120.4 | 132.9 | 140.9 |  | 74.6 | 79.6 | 88.2 | 98.0 | 107.9 | 116.7 | 122.0 |
| 12.0 | 91.0 | 96.6 | 106.8 | 119.3 | 133.2 | 147.1 | 156.0 |  | 80.0 | 85.3 | 94.5 | 105.0 | 115.6 | 125.1 | 130.7 |
| 13.0 | 98.2 | 104.3 | 115.3 | 128.8 | 143.9 | 158.8 | 168.4 |  | 83.7 | 89.3 | 98.9 | 110.0 | 121.1 | 131.0 | 136.8 |
| 14.0 | 103.6 | 110.0 | 121.6 | 135.9 | 151.8 | 167.5 | 177.7 |  | 86.2 | 92.0 | 101.8 | 113.2 | 124.6 | 134.8 | 140.8 |
| 15.0 | 107.6 | 114.3 | 126.3 | 141.1 | 157.6 | 173.9 | 184.5 |  | 87.3 | 93.1 | 103.1 | 114.6 | 126.2 | 136.6 | 142.7 |
| 16.0 | 110.3 | 117.1 | 129.5 | 144.7 | 161.5 | 178.3 | 189.1 |  | 87.6 | 93.4 | 103.5 | 115.0 | 126.6 | 137.0 | 143.1 |
| 17.0 | 111.7 | 118.6 | 131.1 | 146.5 | 163.5 | 180.5 | 191.5 |  | 87.5 | 93.4 | 103.4 | 114.9 | 126.6 | 136.9 | 143.0 |
| 18.0 | 112.3 | 119.2 | 131.8 | 147.3 | 164.4 | 181.5 | 192.5 |  | 87.2 | 93.1 | 103.1 | 114.6 | 126.2 | 136.5 | 142.6 |

RVEDV: Right ventricular end-diastolic volume

**Supplementary Table S10. Centiles of RVESV with inclusion of trabeculations and papillary muscles in volume by age and sex.**

| Age | Boys | | | | | | |  | Girls | | | | | | |
| --- | --- | --- | --- | --- | --- | --- | --- | --- | --- | --- | --- | --- | --- | --- | --- |
|  | 5th | 10th | 25th | 50th | 75th | 90th | 95th |  | 5th | 10th | 25th | 50th | 75th | 90th | 95th |
| 4.0 | 12.6 | 13.9 | 16.1 | 18.8 | 21.7 | 24.5 | 26.2 |  | 9.5 | 10.5 | 12.0 | 13.6 | 15.2 | 16.7 | 17.6 |
| 5.0 | 14.2 | 15.6 | 18.2 | 21.2 | 24.5 | 27.6 | 29.6 |  | 13.5 | 14.9 | 17.0 | 19.3 | 21.5 | 23.6 | 24.9 |
| 6.0 | 16.0 | 17.6 | 20.5 | 23.9 | 27.6 | 31.2 | 33.4 |  | 16.8 | 18.5 | 21.2 | 24.0 | 26.8 | 29.4 | 31.1 |
| 7.0 | 18.2 | 20.0 | 23.3 | 27.2 | 31.4 | 35.4 | 37.9 |  | 19.4 | 21.4 | 24.5 | 27.7 | 30.9 | 33.9 | 35.8 |
| 8.0 | 21.0 | 23.1 | 26.9 | 31.4 | 36.3 | 40.9 | 43.8 |  | 21.8 | 24.0 | 27.5 | 31.1 | 34.7 | 38.1 | 40.2 |
| 9.0 | 24.0 | 26.4 | 30.7 | 35.9 | 41.4 | 46.7 | 50.0 |  | 24.0 | 26.5 | 30.3 | 34.3 | 38.3 | 42.0 | 44.4 |
| 10.0 | 27.3 | 30.1 | 35.0 | 40.9 | 47.2 | 53.2 | 57.0 |  | 26.4 | 29.1 | 33.3 | 37.7 | 42.0 | 46.2 | 48.8 |
| 11.0 | 31.0 | 34.2 | 39.7 | 46.4 | 53.6 | 60.4 | 64.7 |  | 28.8 | 31.7 | 36.3 | 41.1 | 45.8 | 50.3 | 53.2 |
| 12.0 | 34.8 | 38.3 | 44.6 | 52.0 | 60.1 | 67.8 | 72.6 |  | 30.7 | 33.8 | 38.8 | 43.9 | 48.9 | 53.7 | 56.7 |
| 13.0 | 38.0 | 41.9 | 48.7 | 56.9 | 65.6 | 74.0 | 79.3 |  | 32.3 | 35.6 | 40.8 | 46.1 | 51.4 | 56.5 | 59.7 |
| 14.0 | 40.5 | 44.6 | 51.9 | 60.6 | 70.0 | 79.0 | 84.6 |  | 33.6 | 37.0 | 42.4 | 48.0 | 53.5 | 58.8 | 62.1 |
| 15.0 | 42.5 | 46.9 | 54.5 | 63.7 | 73.5 | 82.9 | 88.8 |  | 34.4 | 37.9 | 43.5 | 49.1 | 54.8 | 60.2 | 63.6 |
| 16.0 | 44.1 | 48.6 | 56.5 | 66.0 | 76.2 | 85.9 | 92.0 |  | 34.9 | 38.4 | 44.0 | 49.8 | 55.5 | 61.0 | 64.4 |
| 17.0 | 45.1 | 49.7 | 57.8 | 67.5 | 77.9 | 87.9 | 94.1 |  | 35.2 | 38.8 | 44.4 | 50.2 | 56.0 | 61.6 | 65.0 |
| 18.0 | 45.8 | 50.4 | 58.6 | 68.5 | 79.0 | 89.2 | 95.5 |  | 35.4 | 39.0 | 44.7 | 50.6 | 56.4 | 61.9 | 65.4 |

RVESV: Right ventricular end-systolic volume

**Supplementary Table S11. Centiles of RVSV with inclusion of trabeculations and papillary muscles in volume by age and sex.**

| Age | Boys | | | | | | |  | Girls | | | | | | |
| --- | --- | --- | --- | --- | --- | --- | --- | --- | --- | --- | --- | --- | --- | --- | --- |
|  | 5th | 10th | 25th | 50th | 75th | 90th | 95th |  | 5th | 10th | 25th | 50th | 75th | 90th | 95th |
| 4.0 | 24.5 | 25.8 | 28.1 | 31.1 | 34.6 | 38.4 | 40.9 |  | 20.4 | 21.7 | 24.0 | 26.8 | 29.8 | 32.4 | 34.0 |
| 5.0 | 26.6 | 27.9 | 30.4 | 33.7 | 37.5 | 41.5 | 44.3 |  | 24.8 | 26.4 | 29.3 | 32.7 | 36.3 | 39.5 | 41.4 |
| 6.0 | 29.0 | 30.5 | 33.2 | 36.8 | 40.9 | 45.3 | 48.3 |  | 28.9 | 30.7 | 34.1 | 38.0 | 42.2 | 45.9 | 48.2 |
| 7.0 | 32.0 | 33.6 | 36.6 | 40.5 | 45.1 | 50.0 | 53.3 |  | 32.2 | 34.2 | 38.0 | 42.4 | 47.0 | 51.2 | 53.7 |
| 8.0 | 35.7 | 37.5 | 40.8 | 45.2 | 50.3 | 55.7 | 59.4 |  | 34.8 | 37.1 | 41.1 | 45.9 | 50.9 | 55.4 | 58.1 |
| 9.0 | 39.4 | 41.4 | 45.1 | 49.9 | 55.6 | 61.6 | 65.6 |  | 36.7 | 39.1 | 43.3 | 48.4 | 53.7 | 58.4 | 61.3 |
| 10.0 | 43.5 | 45.8 | 49.9 | 55.2 | 61.4 | 68.0 | 72.6 |  | 39.3 | 41.9 | 46.4 | 51.9 | 57.5 | 62.6 | 65.6 |
| 11.0 | 48.2 | 50.6 | 55.2 | 61.0 | 68.0 | 75.3 | 80.3 |  | 43.1 | 45.9 | 50.8 | 56.8 | 63.0 | 68.6 | 71.9 |
| 12.0 | 52.8 | 55.5 | 60.4 | 66.9 | 74.4 | 82.5 | 88.0 |  | 46.3 | 49.3 | 54.6 | 61.0 | 67.7 | 73.6 | 77.2 |
| 13.0 | 56.5 | 59.4 | 64.7 | 71.6 | 79.7 | 88.3 | 94.2 |  | 48.4 | 51.5 | 57.1 | 63.8 | 70.8 | 77.0 | 80.7 |
| 14.0 | 59.3 | 62.3 | 67.9 | 75.1 | 83.6 | 92.6 | 98.8 |  | 49.5 | 52.7 | 58.4 | 65.3 | 72.4 | 78.8 | 82.6 |
| 15.0 | 61.2 | 64.3 | 70.1 | 77.5 | 86.3 | 95.6 | 102.0 |  | 49.8 | 53.0 | 58.7 | 65.6 | 72.8 | 79.2 | 83.1 |
| 16.0 | 62.5 | 65.7 | 71.6 | 79.2 | 88.2 | 97.7 | 104.2 |  | 49.6 | 52.8 | 58.5 | 65.3 | 72.5 | 78.9 | 82.7 |
| 17.0 | 63.3 | 66.5 | 72.5 | 80.2 | 89.2 | 98.9 | 105.4 |  | 49.2 | 52.4 | 58.0 | 64.8 | 71.9 | 78.3 | 82.0 |
| 18.0 | 63.7 | 67.0 | 73.0 | 80.7 | 89.9 | 99.6 | 106.2 |  | 48.6 | 51.7 | 57.3 | 64.1 | 71.1 | 77.3 | 81.1 |

RVSV: Right ventricular stroke volume

**Supplementary Table S12. Centiles of LVEDV with exclusion of trabeculations and papillary muscles in ventricular volume by BSA and sex.**

| BSA | Boys | | | | | | |  | Girls | | | | | | |
| --- | --- | --- | --- | --- | --- | --- | --- | --- | --- | --- | --- | --- | --- | --- | --- |
|  | 5th | 10th | 25th | 50th | 75th | 90th | 95th |  | 5th | 10th | 25th | 50th | 75th | 90th | 95th |
| 0.6 | 31.4 | 33.2 | 36.3 | 39.7 | 43.1 | 46.3 | 48.1 |  | 26.7 | 28.2 | 30.5 | 32.8 | 35.1 | 36.9 | 38.0 |
| 0.7 | 36.6 | 38.7 | 42.2 | 46.1 | 50.2 | 53.8 | 56.0 |  | 34.0 | 35.9 | 38.8 | 41.9 | 44.7 | 47.1 | 48.5 |
| 0.8 | 41.7 | 44.1 | 48.2 | 52.7 | 57.3 | 61.4 | 63.9 |  | 41.1 | 43.3 | 46.9 | 50.5 | 53.9 | 56.9 | 58.5 |
| 0.9 | 46.9 | 49.6 | 54.1 | 59.2 | 64.4 | 69.0 | 71.8 |  | 47.5 | 50.1 | 54.1 | 58.4 | 62.3 | 65.7 | 67.7 |
| 1.0 | 51.5 | 54.5 | 59.5 | 65.0 | 70.7 | 75.8 | 78.9 |  | 53.2 | 56.1 | 60.7 | 65.5 | 69.9 | 73.7 | 75.9 |
| 1.1 | 57.3 | 60.6 | 66.1 | 72.3 | 78.6 | 84.3 | 87.7 |  | 59.1 | 62.3 | 67.4 | 72.7 | 77.6 | 81.8 | 84.2 |
| 1.2 | 64.2 | 67.9 | 74.1 | 81.0 | 88.0 | 94.4 | 98.3 |  | 64.9 | 68.4 | 74.0 | 79.7 | 85.1 | 89.8 | 92.4 |
| 1.3 | 71.0 | 75.1 | 82.0 | 89.7 | 97.5 | 104.5 | 108.8 |  | 70.6 | 74.5 | 80.5 | 86.8 | 92.7 | 97.7 | 100.6 |
| 1.4 | 78.0 | 82.5 | 90.0 | 98.5 | 107.1 | 114.8 | 119.5 |  | 76.5 | 80.6 | 87.2 | 94.0 | 100.4 | 105.8 | 109.0 |
| 1.5 | 85.2 | 90.1 | 98.4 | 107.6 | 117.0 | 125.4 | 130.5 |  | 81.8 | 86.2 | 93.3 | 100.5 | 107.4 | 113.2 | 116.5 |
| 1.6 | 92.3 | 97.5 | 106.5 | 116.5 | 126.6 | 135.7 | 141.3 |  | 86.3 | 91.1 | 98.5 | 106.2 | 113.4 | 119.5 | 123.0 |
| 1.7 | 98.7 | 104.3 | 113.9 | 124.6 | 135.4 | 145.2 | 151.1 |  | 90.4 | 95.3 | 103.1 | 111.1 | 118.6 | 125.1 | 128.8 |
| 1.8 | 104.5 | 110.5 | 120.6 | 131.9 | 143.4 | 153.8 | 160.0 |  | 94.3 | 99.5 | 107.6 | 115.9 | 123.8 | 130.5 | 134.4 |
| 1.9 | 110.2 | 116.5 | 127.2 | 139.1 | 151.2 | 162.2 | 168.8 |  | 98.2 | 103.6 | 112.0 | 120.8 | 129.0 | 136.0 | 140.0 |

BSA: Body surface area; LVEDV: Left ventricular end-diastolic volume

**Supplementary Table S13. Centiles of LVESV with exclusion of trabeculations and papillary muscles in ventricular volume by BSA and sex.**

| BSA | Boys | | | | | | |  | Girls | | | | | | |
| --- | --- | --- | --- | --- | --- | --- | --- | --- | --- | --- | --- | --- | --- | --- | --- |
|  | 5th | 10th | 25th | 50th | 75th | 90th | 95th |  | 5th | 10th | 25th | 50th | 75th | 90th | 95th |
| 0.6 | 9.0 | 9.6 | 10.8 | 12.2 | 13.7 | 14.8 | 15.4 |  | 6.9 | 7.5 | 8.5 | 9.6 | 10.5 | 11.1 | 11.4 |
| 0.7 | 10.4 | 11.2 | 12.5 | 14.2 | 15.9 | 17.2 | 17.9 |  | 9.5 | 10.3 | 11.6 | 13.2 | 14.5 | 15.3 | 15.7 |
| 0.8 | 12.0 | 12.8 | 14.4 | 16.3 | 18.2 | 19.7 | 20.5 |  | 12.0 | 12.9 | 14.6 | 16.5 | 18.2 | 19.2 | 19.7 |
| 0.9 | 13.7 | 14.6 | 16.4 | 18.6 | 20.8 | 22.5 | 23.4 |  | 14.0 | 15.2 | 17.1 | 19.4 | 21.3 | 22.5 | 23.0 |
| 1.0 | 15.1 | 16.2 | 18.1 | 20.5 | 23.0 | 24.8 | 25.8 |  | 15.6 | 16.9 | 19.1 | 21.6 | 23.7 | 25.0 | 25.7 |
| 1.1 | 16.8 | 18.0 | 20.2 | 22.9 | 25.6 | 27.7 | 28.8 |  | 17.2 | 18.6 | 21.0 | 23.8 | 26.1 | 27.6 | 28.3 |
| 1.2 | 19.1 | 20.4 | 22.9 | 26.0 | 29.0 | 31.4 | 32.7 |  | 18.9 | 20.4 | 23.0 | 26.0 | 28.6 | 30.2 | 30.9 |
| 1.3 | 21.4 | 22.9 | 25.7 | 29.1 | 32.6 | 35.2 | 36.7 |  | 20.7 | 22.4 | 25.3 | 28.6 | 31.4 | 33.2 | 34.0 |
| 1.4 | 23.9 | 25.6 | 28.7 | 32.6 | 36.4 | 39.4 | 41.0 |  | 23.0 | 24.9 | 28.1 | 31.8 | 35.0 | 36.9 | 37.8 |
| 1.5 | 26.7 | 28.6 | 32.0 | 36.4 | 40.6 | 44.0 | 45.7 |  | 25.6 | 27.6 | 31.2 | 35.3 | 38.8 | 41.0 | 42.0 |
| 1.6 | 29.5 | 31.5 | 35.3 | 40.1 | 44.8 | 48.5 | 50.4 |  | 28.0 | 30.3 | 34.2 | 38.7 | 42.5 | 44.9 | 46.0 |
| 1.7 | 31.7 | 34.0 | 38.0 | 43.2 | 48.2 | 52.2 | 54.3 |  | 30.3 | 32.8 | 37.0 | 41.9 | 46.0 | 48.6 | 49.8 |
| 1.8 | 33.5 | 35.9 | 40.2 | 45.6 | 50.9 | 55.1 | 57.4 |  | 32.7 | 35.3 | 39.9 | 45.1 | 49.6 | 52.4 | 53.6 |
| 1.9 | 35.2 | 37.7 | 42.2 | 47.9 | 53.5 | 57.9 | 60.3 |  | 35.0 | 37.8 | 42.7 | 48.4 | 53.1 | 56.1 | 57.5 |

BSA: Body surface area; LVESV: Left ventricular end-systolic volume

**Supplementary Table S14. Centiles of LVSV with exclusion of trabeculations and papillary muscles in ventricular volume by BSA and sex.**

| BSA | Boys | | | | | | |  | Girls | | | | | | |
| --- | --- | --- | --- | --- | --- | --- | --- | --- | --- | --- | --- | --- | --- | --- | --- |
|  | 5th | 10th | 25th | 50th | 75th | 90th | 95th |  | 5th | 10th | 25th | 50th | 75th | 90th | 95th |
| 0.6 | 21.9 | 23.0 | 24.9 | 27.2 | 29.6 | 32.0 | 33.6 |  | 18.8 | 19.8 | 21.3 | 23.0 | 24.6 | 26.1 | 26.9 |
| 0.7 | 25.5 | 26.8 | 29.1 | 31.7 | 34.5 | 37.3 | 39.2 |  | 23.4 | 24.6 | 26.5 | 28.6 | 30.7 | 32.5 | 33.5 |
| 0.8 | 29.1 | 30.6 | 33.1 | 36.1 | 39.4 | 42.6 | 44.6 |  | 27.8 | 29.2 | 31.5 | 34.0 | 36.4 | 38.6 | 39.8 |
| 0.9 | 32.4 | 34.1 | 36.9 | 40.2 | 43.8 | 47.4 | 49.7 |  | 31.8 | 33.5 | 36.1 | 39.0 | 41.7 | 44.2 | 45.6 |
| 1.0 | 35.4 | 37.2 | 40.3 | 44.0 | 47.9 | 51.8 | 54.3 |  | 35.7 | 37.6 | 40.6 | 43.8 | 46.9 | 49.6 | 51.3 |
| 1.1 | 39.4 | 41.4 | 44.8 | 48.9 | 53.3 | 57.6 | 60.4 |  | 39.8 | 41.8 | 45.1 | 48.7 | 52.1 | 55.2 | 57.0 |
| 1.2 | 44.2 | 46.4 | 50.3 | 54.8 | 59.8 | 64.6 | 67.7 |  | 43.6 | 45.9 | 49.6 | 53.4 | 57.2 | 60.6 | 62.6 |
| 1.3 | 48.9 | 51.4 | 55.7 | 60.7 | 66.2 | 71.6 | 75.0 |  | 47.4 | 49.9 | 53.8 | 58.0 | 62.2 | 65.8 | 68.0 |
| 1.4 | 53.5 | 56.2 | 60.9 | 66.4 | 72.4 | 78.3 | 82.0 |  | 51.1 | 53.7 | 58.0 | 62.5 | 66.9 | 70.9 | 73.2 |
| 1.5 | 57.8 | 60.7 | 65.8 | 71.7 | 78.1 | 84.5 | 88.6 |  | 54.2 | 57.0 | 61.5 | 66.4 | 71.1 | 75.3 | 77.7 |
| 1.6 | 61.6 | 64.7 | 70.1 | 76.5 | 83.3 | 90.1 | 94.4 |  | 56.8 | 59.8 | 64.5 | 69.6 | 74.5 | 78.9 | 81.5 |
| 1.7 | 65.1 | 68.4 | 74.1 | 80.8 | 88.1 | 95.2 | 99.8 |  | 59.1 | 62.1 | 67.1 | 72.3 | 77.5 | 82.0 | 84.7 |
| 1.8 | 68.4 | 71.8 | 77.8 | 84.9 | 92.5 | 100.0 | 104.9 |  | 61.3 | 64.5 | 69.6 | 75.0 | 80.4 | 85.1 | 87.9 |
| 1.9 | 71.7 | 75.3 | 81.6 | 88.9 | 96.9 | 104.8 | 109.9 |  | 63.5 | 66.8 | 72.1 | 77.8 | 83.3 | 88.2 | 91.1 |

BSA: Body surface area; LVSV: Left ventricular stroke volume

**Supplementary Table S15. Centiles of LVM with exclusion of trabeculations and papillary muscles in ventricular volume by BSA and sex.**

| BSA | Boys | | | | | | |  | Girls | | | | | | |
| --- | --- | --- | --- | --- | --- | --- | --- | --- | --- | --- | --- | --- | --- | --- | --- |
|  | 5th | 10th | 25th | 50th | 75th | 90th | 95th |  | 5th | 10th | 25th | 50th | 75th | 90th | 95th |
| 0.6 | 19.4 | 20.4 | 22.2 | 24.2 | 26.3 | 28.6 | 30.1 |  | 20.1 | 20.8 | 22.0 | 23.5 | 25.2 | 26.8 | 27.7 |
| 0.7 | 23.5 | 24.8 | 27.0 | 29.4 | 32.0 | 34.7 | 36.6 |  | 24.2 | 25.0 | 26.4 | 28.3 | 30.3 | 32.2 | 33.3 |
| 0.8 | 27.8 | 29.3 | 31.9 | 34.7 | 37.8 | 41.0 | 43.2 |  | 28.1 | 29.0 | 30.7 | 32.9 | 35.2 | 37.4 | 38.7 |
| 0.9 | 32.2 | 33.9 | 36.9 | 40.2 | 43.7 | 47.5 | 50.0 |  | 31.8 | 32.8 | 34.8 | 37.2 | 39.8 | 42.3 | 43.8 |
| 1.0 | 36.1 | 38.1 | 41.4 | 45.1 | 49.1 | 53.3 | 56.1 |  | 35.6 | 36.8 | 38.9 | 41.6 | 44.6 | 47.4 | 49.1 |
| 1.1 | 40.6 | 42.8 | 46.5 | 50.7 | 55.2 | 59.9 | 63.1 |  | 39.6 | 40.9 | 43.3 | 46.4 | 49.7 | 52.7 | 54.6 |
| 1.2 | 46.0 | 48.5 | 52.7 | 57.4 | 62.5 | 67.9 | 71.5 |  | 43.7 | 45.2 | 47.8 | 51.2 | 54.8 | 58.2 | 60.3 |
| 1.3 | 51.2 | 54.0 | 58.7 | 63.9 | 69.5 | 75.5 | 79.5 |  | 48.0 | 49.6 | 52.5 | 56.1 | 60.1 | 63.9 | 66.1 |
| 1.4 | 56.4 | 59.5 | 64.6 | 70.4 | 76.6 | 83.2 | 87.6 |  | 51.6 | 53.3 | 56.4 | 60.4 | 64.7 | 68.7 | 71.1 |
| 1.5 | 61.7 | 65.0 | 70.7 | 76.9 | 83.8 | 91.0 | 95.8 |  | 55.0 | 56.8 | 60.2 | 64.4 | 68.9 | 73.2 | 75.8 |
| 1.6 | 66.3 | 69.9 | 76.0 | 82.8 | 90.1 | 97.8 | 103.0 |  | 58.5 | 60.4 | 63.9 | 68.4 | 73.3 | 77.8 | 80.6 |
| 1.7 | 70.5 | 74.4 | 80.8 | 88.0 | 95.8 | 104.1 | 109.6 |  | 62.0 | 64.0 | 67.7 | 72.5 | 77.6 | 82.4 | 85.4 |
| 1.8 | 74.7 | 78.7 | 85.6 | 93.2 | 101.5 | 110.2 | 116.1 |  | 65.4 | 67.6 | 71.5 | 76.5 | 82.0 | 87.0 | 90.2 |
| 1.9 | 78.9 | 83.2 | 90.4 | 98.4 | 107.2 | 116.4 | 122.6 |  | 68.9 | 71.2 | 75.3 | 80.6 | 86.3 | 91.6 | 94.9 |

BSA: Body surface area; LVM: Left ventricular mass

**Supplementary Table S16. Centiles of RVEDV with exclusion of trabeculations and papillary muscles in ventricular volume by BSA and sex.**

| BSA | Boys | | | | | | |  | Girls | | | | | | |
| --- | --- | --- | --- | --- | --- | --- | --- | --- | --- | --- | --- | --- | --- | --- | --- |
|  | 5th | 10th | 25th | 50th | 75th | 90th | 95th |  | 5th | 10th | 25th | 50th | 75th | 90th | 95th |
| 0.6 | 32.3 | 34.5 | 38.0 | 41.5 | 45.2 | 49.4 | 52.4 |  | 27.7 | 28.9 | 31.0 | 33.4 | 35.8 | 38.0 | 39.4 |
| 0.7 | 36.9 | 39.4 | 43.4 | 47.4 | 51.7 | 56.5 | 59.9 |  | 34.9 | 36.4 | 39.0 | 42.0 | 45.0 | 47.8 | 49.5 |
| 0.8 | 41.8 | 44.6 | 49.1 | 53.7 | 58.5 | 63.9 | 67.7 |  | 42.0 | 43.8 | 47.0 | 50.5 | 54.2 | 57.5 | 59.6 |
| 0.9 | 46.8 | 49.9 | 55.0 | 60.0 | 65.4 | 71.5 | 75.8 |  | 48.6 | 50.7 | 54.4 | 58.5 | 62.7 | 66.6 | 69.0 |
| 1.0 | 51.8 | 55.3 | 60.9 | 66.5 | 72.4 | 79.2 | 83.9 |  | 54.9 | 57.3 | 61.5 | 66.1 | 70.9 | 75.3 | 78.0 |
| 1.1 | 58.6 | 62.5 | 68.9 | 75.2 | 82.0 | 89.6 | 94.9 |  | 62.1 | 64.8 | 69.4 | 74.7 | 80.1 | 85.1 | 88.1 |
| 1.2 | 66.6 | 71.1 | 78.3 | 85.5 | 93.2 | 101.9 | 107.9 |  | 69.2 | 72.2 | 77.4 | 83.3 | 89.3 | 94.9 | 98.2 |
| 1.3 | 74.4 | 79.4 | 87.4 | 95.5 | 104.1 | 113.8 | 120.5 |  | 75.6 | 78.9 | 84.5 | 91.0 | 97.6 | 103.6 | 107.3 |
| 1.4 | 82.3 | 87.9 | 96.8 | 105.7 | 115.2 | 125.9 | 133.4 |  | 81.7 | 85.3 | 91.4 | 98.3 | 105.4 | 112.0 | 115.9 |
| 1.5 | 90.9 | 97.0 | 106.8 | 116.7 | 127.2 | 139.0 | 147.3 |  | 87.8 | 91.7 | 98.2 | 105.7 | 113.3 | 120.4 | 124.6 |
| 1.6 | 99.4 | 106.1 | 116.8 | 127.6 | 139.1 | 152.0 | 161.1 |  | 94.0 | 98.2 | 105.2 | 113.2 | 121.4 | 128.9 | 133.5 |
| 1.7 | 107.6 | 114.9 | 126.5 | 138.2 | 150.6 | 164.6 | 174.4 |  | 100.2 | 104.6 | 112.1 | 120.7 | 129.4 | 137.4 | 142.3 |
| 1.8 | 115.6 | 123.5 | 135.9 | 148.5 | 161.8 | 176.9 | 187.4 |  | 106.4 | 111.1 | 119.1 | 128.1 | 137.4 | 145.9 | 151.1 |
| 1.9 | 123.7 | 132.1 | 145.4 | 158.9 | 173.2 | 189.2 | 200.5 |  | 112.6 | 117.6 | 126.0 | 135.6 | 145.4 | 154.4 | 159.9 |

BSA: Body surface area; RVEDV: Right ventricular end-diastolic volume

**Supplementary Table S17. Centiles of RVESV with exclusion of trabeculations and papillary muscles in ventricular volume by BSA and sex.**

| BSA | Boys | | | | | | |  | Girls | | | | | | |
| --- | --- | --- | --- | --- | --- | --- | --- | --- | --- | --- | --- | --- | --- | --- | --- |
|  | 5th | 10th | 25th | 50th | 75th | 90th | 95th |  | 5th | 10th | 25th | 50th | 75th | 90th | 95th |
| 0.6 | 9.6 | 10.8 | 12.9 | 15.3 | 17.8 | 20.0 | 21.2 |  | 6.5 | 7.1 | 8.1 | 9.2 | 10.4 | 11.5 | 12.2 |
| 0.7 | 10.9 | 12.3 | 14.7 | 17.5 | 20.3 | 22.8 | 24.2 |  | 9.1 | 9.9 | 11.3 | 12.9 | 14.5 | 16.0 | 17.0 |
| 0.8 | 12.4 | 14.0 | 16.7 | 19.9 | 23.1 | 25.9 | 27.6 |  | 11.7 | 12.7 | 14.5 | 16.6 | 18.7 | 20.6 | 21.8 |
| 0.9 | 14.0 | 15.7 | 18.8 | 22.4 | 26.0 | 29.1 | 31.0 |  | 14.1 | 15.4 | 17.5 | 20.0 | 22.6 | 24.9 | 26.4 |
| 1.0 | 15.0 | 16.9 | 20.2 | 24.0 | 27.9 | 31.3 | 33.3 |  | 16.3 | 17.7 | 20.2 | 23.1 | 26.0 | 28.8 | 30.4 |
| 1.1 | 16.6 | 18.7 | 22.4 | 26.6 | 30.9 | 34.7 | 36.9 |  | 18.8 | 20.5 | 23.4 | 26.7 | 30.1 | 33.3 | 35.2 |
| 1.2 | 19.2 | 21.6 | 25.7 | 30.6 | 35.6 | 39.9 | 42.5 |  | 21.4 | 23.3 | 26.6 | 30.4 | 34.3 | 37.9 | 40.1 |
| 1.3 | 21.7 | 24.5 | 29.2 | 34.8 | 40.4 | 45.3 | 48.2 |  | 23.5 | 25.6 | 29.2 | 33.3 | 37.6 | 41.5 | 43.9 |
| 1.4 | 24.4 | 27.5 | 32.8 | 39.1 | 45.4 | 50.9 | 54.1 |  | 25.3 | 27.6 | 31.5 | 36.0 | 40.6 | 44.8 | 47.4 |
| 1.5 | 27.6 | 31.1 | 37.1 | 44.1 | 51.3 | 57.5 | 61.2 |  | 27.6 | 30.1 | 34.3 | 39.2 | 44.2 | 48.8 | 51.6 |
| 1.6 | 30.9 | 34.8 | 41.6 | 49.5 | 57.5 | 64.5 | 68.6 |  | 30.3 | 33.0 | 37.7 | 43.0 | 48.5 | 53.6 | 56.7 |
| 1.7 | 34.2 | 38.5 | 45.9 | 54.7 | 63.5 | 71.3 | 75.8 |  | 33.2 | 36.2 | 41.3 | 47.2 | 53.2 | 58.8 | 62.2 |
| 1.8 | 37.4 | 42.0 | 50.2 | 59.7 | 69.4 | 77.9 | 82.8 |  | 36.2 | 39.4 | 45.0 | 51.4 | 58.0 | 64.0 | 67.7 |
| 1.9 | 40.6 | 45.6 | 54.5 | 64.9 | 75.4 | 84.5 | 89.9 |  | 39.1 | 42.7 | 48.7 | 55.6 | 62.7 | 69.3 | 73.3 |

BSA: Body surface area; RVESV: Right ventricular end-systolic volume

**Supplementary Table S18. Centiles of RVSV with exclusion of trabeculations and papillary muscles in ventricular volume by BSA and sex.**

| BSA | Boys | | | | | | |  | Girls | | | | | | |
| --- | --- | --- | --- | --- | --- | --- | --- | --- | --- | --- | --- | --- | --- | --- | --- |
|  | 5th | 10th | 25th | 50th | 75th | 90th | 95th |  | 5th | 10th | 25th | 50th | 75th | 90th | 95th |
| 0.6 | 20.0 | 21.1 | 22.9 | 24.8 | 27.0 | 29.2 | 30.7 |  | 19.9 | 20.7 | 22.2 | 23.9 | 25.7 | 27.2 | 28.1 |
| 0.7 | 24.1 | 25.4 | 27.5 | 29.9 | 32.4 | 35.1 | 36.9 |  | 24.1 | 25.1 | 26.8 | 29.0 | 31.1 | 33.0 | 34.0 |
| 0.8 | 28.1 | 29.6 | 32.0 | 34.8 | 37.8 | 41.0 | 43.1 |  | 28.2 | 29.3 | 31.4 | 33.9 | 36.4 | 38.6 | 39.8 |
| 0.9 | 31.8 | 33.5 | 36.3 | 39.4 | 42.8 | 46.4 | 48.8 |  | 32.0 | 33.3 | 35.7 | 38.5 | 41.4 | 43.8 | 45.3 |
| 1.0 | 35.2 | 37.0 | 40.1 | 43.6 | 47.3 | 51.3 | 53.9 |  | 35.9 | 37.4 | 40.0 | 43.2 | 46.4 | 49.2 | 50.7 |
| 1.1 | 39.3 | 41.3 | 44.8 | 48.7 | 52.9 | 57.3 | 60.2 |  | 40.0 | 41.7 | 44.6 | 48.1 | 51.7 | 54.8 | 56.5 |
| 1.2 | 44.1 | 46.4 | 50.3 | 54.6 | 59.3 | 64.3 | 67.6 |  | 43.9 | 45.8 | 49.0 | 52.9 | 56.8 | 60.2 | 62.1 |
| 1.3 | 48.8 | 51.4 | 55.7 | 60.5 | 65.7 | 71.2 | 74.8 |  | 47.8 | 49.7 | 53.2 | 57.5 | 61.7 | 65.4 | 67.5 |
| 1.4 | 53.4 | 56.2 | 60.9 | 66.2 | 71.9 | 77.9 | 81.9 |  | 51.6 | 53.7 | 57.5 | 62.0 | 66.7 | 70.6 | 72.9 |
| 1.5 | 57.7 | 60.7 | 65.8 | 71.5 | 77.6 | 84.1 | 88.4 |  | 54.9 | 57.2 | 61.2 | 66.1 | 71.0 | 75.2 | 77.6 |
| 1.6 | 61.5 | 64.7 | 70.2 | 76.2 | 82.8 | 89.7 | 94.3 |  | 57.8 | 60.2 | 64.4 | 69.5 | 74.7 | 79.2 | 81.7 |
| 1.7 | 65.0 | 68.4 | 74.2 | 80.6 | 87.5 | 94.8 | 99.7 |  | 60.4 | 62.9 | 67.3 | 72.6 | 78.1 | 82.7 | 85.3 |
| 1.8 | 68.2 | 71.8 | 77.8 | 84.5 | 91.8 | 99.5 | 104.6 |  | 62.9 | 65.5 | 70.1 | 75.7 | 81.3 | 86.1 | 88.9 |
| 1.9 | 71.3 | 75.1 | 81.4 | 88.4 | 96.0 | 104.0 | 109.4 |  | 65.5 | 68.2 | 72.9 | 78.7 | 84.6 | 89.6 | 92.5 |

BSA: Body surface area; RVSV: Right ventricular stroke volume

**Supplementary Table S19. Centiles of LVEDV with exclusion of trabeculations and papillary muscles in volume by age and sex.**

| Age | Boys | | | | | | |  | Girls | | | | | | |
| --- | --- | --- | --- | --- | --- | --- | --- | --- | --- | --- | --- | --- | --- | --- | --- |
|  | 5th | 10th | 25th | 50th | 75th | 90th | 95th |  | 5th | 10th | 25th | 50th | 75th | 90th | 95th |
| 4.0 | 35.0 | 36.7 | 40.1 | 45.3 | 51.2 | 56.5 | 59.6 |  | 28.4 | 30.0 | 33.0 | 36.8 | 40.7 | 44.1 | 46.1 |
| 5.0 | 37.8 | 39.6 | 43.4 | 48.9 | 55.3 | 61.0 | 64.3 |  | 35.9 | 37.9 | 41.7 | 46.5 | 51.5 | 55.8 | 58.2 |
| 6.0 | 41.2 | 43.2 | 47.3 | 53.4 | 60.4 | 66.6 | 70.2 |  | 42.6 | 45.0 | 49.5 | 55.2 | 61.1 | 66.2 | 69.1 |
| 7.0 | 45.6 | 47.8 | 52.4 | 59.1 | 66.9 | 73.7 | 77.7 |  | 48.1 | 50.8 | 55.8 | 62.2 | 69.0 | 74.7 | 78.0 |
| 8.0 | 51.1 | 53.6 | 58.7 | 66.2 | 74.9 | 82.5 | 87.0 |  | 52.6 | 55.5 | 61.0 | 68.1 | 75.4 | 81.7 | 85.3 |
| 9.0 | 56.5 | 59.3 | 64.9 | 73.2 | 82.9 | 91.4 | 96.3 |  | 56.0 | 59.2 | 65.1 | 72.5 | 80.4 | 87.1 | 90.9 |
| 10.0 | 62.4 | 65.4 | 71.7 | 80.8 | 91.5 | 100.8 | 106.3 |  | 60.3 | 63.7 | 70.0 | 78.1 | 86.5 | 93.7 | 97.8 |
| 11.0 | 69.2 | 72.6 | 79.5 | 89.7 | 101.5 | 111.9 | 118.0 |  | 65.8 | 69.5 | 76.4 | 85.2 | 94.4 | 102.2 | 106.7 |
| 12.0 | 76.5 | 80.3 | 87.9 | 99.2 | 112.2 | 123.7 | 130.4 |  | 70.5 | 74.4 | 81.8 | 91.2 | 101.1 | 109.5 | 114.3 |
| 13.0 | 82.8 | 86.8 | 95.1 | 107.2 | 121.3 | 133.8 | 141.0 |  | 73.7 | 77.9 | 85.6 | 95.5 | 105.8 | 114.6 | 119.6 |
| 14.0 | 87.1 | 91.4 | 100.1 | 112.9 | 127.7 | 140.8 | 148.5 |  | 75.9 | 80.2 | 88.1 | 98.3 | 108.9 | 117.9 | 123.1 |
| 15.0 | 89.8 | 94.2 | 103.2 | 116.4 | 131.7 | 145.2 | 153.1 |  | 77.1 | 81.5 | 89.6 | 99.9 | 110.7 | 119.9 | 125.2 |
| 16.0 | 91.3 | 95.8 | 104.9 | 118.3 | 133.8 | 147.6 | 155.6 |  | 77.9 | 82.3 | 90.4 | 100.9 | 111.8 | 121.0 | 126.4 |
| 17.0 | 91.6 | 96.1 | 105.3 | 118.7 | 134.3 | 148.1 | 156.1 |  | 78.4 | 82.8 | 91.0 | 101.5 | 112.5 | 121.8 | 127.2 |
| 18.0 | 91.4 | 95.8 | 105.0 | 118.4 | 134.0 | 147.7 | 155.7 |  | 78.7 | 83.1 | 91.4 | 101.9 | 112.9 | 122.3 | 127.6 |

LVEDV: Left ventricular end-diastolic volume

**Supplementary Table S20. Centiles of LVESV with exclusion of trabeculations and papillary muscles in volume by age and sex.**

| Age | Boys | | | | | | |  | Girls | | | | | | |
| --- | --- | --- | --- | --- | --- | --- | --- | --- | --- | --- | --- | --- | --- | --- | --- |
|  | 5th | 10th | 25th | 50th | 75th | 90th | 95th |  | 5th | 10th | 25th | 50th | 75th | 90th | 95th |
| 4.0 | 10.3 | 11.1 | 12.6 | 14.5 | 16.6 | 18.7 | 20.2 |  | 7.8 | 8.5 | 9.6 | 10.8 | 12.0 | 13.1 | 13.8 |
| 5.0 | 11.1 | 12.0 | 13.6 | 15.6 | 17.9 | 20.2 | 21.7 |  | 10.4 | 11.3 | 12.9 | 14.5 | 16.1 | 17.6 | 18.5 |
| 6.0 | 12.2 | 13.1 | 14.8 | 17.0 | 19.5 | 22.1 | 23.8 |  | 12.7 | 13.8 | 15.7 | 17.7 | 19.7 | 21.5 | 22.6 |
| 7.0 | 13.4 | 14.5 | 16.4 | 18.8 | 21.5 | 24.4 | 26.2 |  | 14.4 | 15.7 | 17.9 | 20.1 | 22.4 | 24.4 | 25.7 |
| 8.0 | 14.9 | 16.0 | 18.1 | 20.8 | 23.9 | 27.0 | 29.0 |  | 15.8 | 17.2 | 19.6 | 22.1 | 24.5 | 26.8 | 28.2 |
| 9.0 | 16.3 | 17.5 | 19.9 | 22.8 | 26.2 | 29.6 | 31.8 |  | 17.1 | 18.7 | 21.2 | 23.9 | 26.5 | 29.0 | 30.5 |
| 10.0 | 18.0 | 19.4 | 22.0 | 25.2 | 28.9 | 32.7 | 35.2 |  | 18.7 | 20.4 | 23.2 | 26.1 | 29.1 | 31.7 | 33.4 |
| 11.0 | 20.3 | 21.9 | 24.8 | 28.4 | 32.6 | 36.9 | 39.7 |  | 20.6 | 22.5 | 25.5 | 28.8 | 32.0 | 34.9 | 36.7 |
| 12.0 | 23.2 | 25.0 | 28.3 | 32.5 | 37.2 | 42.1 | 45.3 |  | 22.1 | 24.2 | 27.4 | 30.9 | 34.3 | 37.5 | 39.4 |
| 13.0 | 25.8 | 27.8 | 31.5 | 36.1 | 41.4 | 46.8 | 50.4 |  | 23.3 | 25.4 | 28.8 | 32.4 | 36.1 | 39.4 | 41.4 |
| 14.0 | 27.5 | 29.7 | 33.6 | 38.6 | 44.2 | 50.0 | 53.8 |  | 24.1 | 26.3 | 29.9 | 33.7 | 37.4 | 40.9 | 43.0 |
| 15.0 | 28.5 | 30.8 | 34.8 | 40.0 | 45.8 | 51.8 | 55.8 |  | 24.9 | 27.1 | 30.8 | 34.7 | 38.5 | 42.1 | 44.3 |
| 16.0 | 29.0 | 31.3 | 35.4 | 40.6 | 46.6 | 52.7 | 56.7 |  | 25.3 | 27.7 | 31.4 | 35.4 | 39.3 | 42.9 | 45.1 |
| 17.0 | 29.0 | 31.2 | 35.4 | 40.6 | 46.5 | 52.6 | 56.6 |  | 25.5 | 27.8 | 31.6 | 35.6 | 39.5 | 43.2 | 45.4 |
| 18.0 | 28.7 | 30.9 | 35.0 | 40.2 | 46.0 | 52.1 | 56.0 |  | 25.4 | 27.7 | 31.4 | 35.4 | 39.3 | 43.0 | 45.2 |

LVESV: Left ventricular end-systolic volume

**Supplementary Table S21. Centiles of LVSV with exclusion of trabeculations and papillary muscles in volume by age and sex.**

| Age | Boys | | | | | | |  | Girls | | | | | | |
| --- | --- | --- | --- | --- | --- | --- | --- | --- | --- | --- | --- | --- | --- | --- | --- |
|  | 5th | 10th | 25th | 50th | 75th | 90th | 95th |  | 5th | 10th | 25th | 50th | 75th | 90th | 95th |
| 4.0 | 24.4 | 25.8 | 28.3 | 31.4 | 35.0 | 38.6 | 41.0 |  | 18.8 | 20.2 | 22.4 | 24.7 | 27.2 | 29.6 | 31.2 |
| 5.0 | 26.3 | 27.7 | 30.4 | 33.8 | 37.6 | 41.5 | 44.1 |  | 23.9 | 25.7 | 28.5 | 31.5 | 34.6 | 37.7 | 39.7 |
| 6.0 | 28.5 | 30.0 | 32.9 | 36.6 | 40.7 | 45.0 | 47.8 |  | 28.4 | 30.5 | 33.9 | 37.4 | 41.1 | 44.8 | 47.2 |
| 7.0 | 31.2 | 32.9 | 36.1 | 40.1 | 44.7 | 49.3 | 52.4 |  | 32.1 | 34.4 | 38.2 | 42.2 | 46.4 | 50.5 | 53.2 |
| 8.0 | 34.8 | 36.8 | 40.3 | 44.8 | 49.8 | 55.0 | 58.5 |  | 35.1 | 37.6 | 41.8 | 46.2 | 50.7 | 55.2 | 58.2 |
| 9.0 | 38.6 | 40.7 | 44.6 | 49.6 | 55.2 | 61.0 | 64.8 |  | 37.3 | 40.0 | 44.5 | 49.1 | 53.9 | 58.8 | 61.9 |
| 10.0 | 42.8 | 45.2 | 49.5 | 55.0 | 61.2 | 67.6 | 71.8 |  | 40.0 | 42.9 | 47.6 | 52.7 | 57.8 | 63.0 | 66.3 |
| 11.0 | 47.5 | 50.1 | 54.9 | 61.0 | 67.9 | 75.0 | 79.7 |  | 43.6 | 46.8 | 51.9 | 57.4 | 63.0 | 68.6 | 72.3 |
| 12.0 | 52.2 | 55.1 | 60.4 | 67.0 | 74.6 | 82.4 | 87.6 |  | 46.6 | 50.0 | 55.6 | 61.4 | 67.4 | 73.4 | 77.3 |
| 13.0 | 56.0 | 59.1 | 64.8 | 71.9 | 80.1 | 88.5 | 94.0 |  | 48.7 | 52.2 | 58.0 | 64.1 | 70.4 | 76.7 | 80.7 |
| 14.0 | 58.7 | 61.9 | 67.9 | 75.4 | 83.9 | 92.7 | 98.5 |  | 49.9 | 53.6 | 59.5 | 65.7 | 72.2 | 78.6 | 82.8 |
| 15.0 | 60.3 | 63.6 | 69.7 | 77.4 | 86.2 | 95.2 | 101.2 |  | 50.4 | 54.0 | 60.0 | 66.3 | 72.8 | 79.4 | 83.5 |
| 16.0 | 61.1 | 64.5 | 70.7 | 78.5 | 87.4 | 96.5 | 102.5 |  | 50.4 | 54.0 | 60.0 | 66.3 | 72.8 | 79.3 | 83.5 |
| 17.0 | 61.2 | 64.6 | 70.8 | 78.6 | 87.6 | 96.7 | 102.7 |  | 50.2 | 53.9 | 59.8 | 66.1 | 72.6 | 79.1 | 83.3 |
| 18.0 | 61.0 | 64.4 | 70.5 | 78.3 | 87.2 | 96.3 | 102.4 |  | 49.9 | 53.5 | 59.4 | 65.7 | 72.1 | 78.6 | 82.7 |

LVSV: Left ventricular stroke volume

**Supplementary Table S22. Centiles of LVM with exclusion of trabeculations and papillary muscles in volume by age and sex.**

| Age | Boys | | | | | | |  | Girls | | | | | | |
| --- | --- | --- | --- | --- | --- | --- | --- | --- | --- | --- | --- | --- | --- | --- | --- |
|  | 5th | 10th | 25th | 50th | 75th | 90th | 95th |  | 5th | 10th | 25th | 50th | 75th | 90th | 95th |
| 4.0 | 21.1 | 22.6 | 25.4 | 28.8 | 32.7 | 36.6 | 39.1 |  | 19.3 | 20.6 | 22.8 | 25.4 | 28.0 | 30.2 | 31.6 |
| 5.0 | 23.5 | 25.2 | 28.3 | 32.1 | 36.4 | 40.8 | 43.6 |  | 23.7 | 25.3 | 28.0 | 31.2 | 34.4 | 37.1 | 38.8 |
| 6.0 | 26.5 | 28.4 | 31.8 | 36.2 | 41.0 | 45.9 | 49.1 |  | 27.6 | 29.4 | 32.6 | 36.3 | 40.0 | 43.3 | 45.2 |
| 7.0 | 30.0 | 32.1 | 36.1 | 41.0 | 46.5 | 52.0 | 55.6 |  | 30.8 | 32.8 | 36.3 | 40.4 | 44.5 | 48.1 | 50.3 |
| 8.0 | 34.3 | 36.8 | 41.3 | 46.9 | 53.2 | 59.5 | 63.6 |  | 33.5 | 35.7 | 39.5 | 44.0 | 48.5 | 52.5 | 54.8 |
| 9.0 | 38.2 | 41.0 | 46.0 | 52.2 | 59.2 | 66.3 | 70.9 |  | 36.2 | 38.6 | 42.7 | 47.6 | 52.5 | 56.7 | 59.2 |
| 10.0 | 42.2 | 45.3 | 50.8 | 57.7 | 65.5 | 73.3 | 78.3 |  | 39.4 | 42.0 | 46.5 | 51.7 | 57.0 | 61.7 | 64.4 |
| 11.0 | 47.0 | 50.4 | 56.6 | 64.2 | 72.9 | 81.5 | 87.2 |  | 43.0 | 45.8 | 50.7 | 56.5 | 62.3 | 67.3 | 70.3 |
| 12.0 | 52.0 | 55.8 | 62.6 | 71.1 | 80.6 | 90.2 | 96.5 |  | 46.0 | 49.0 | 54.3 | 60.4 | 66.6 | 72.0 | 75.2 |
| 13.0 | 55.9 | 60.0 | 67.3 | 76.4 | 86.7 | 97.0 | 103.7 |  | 48.2 | 51.3 | 56.8 | 63.2 | 69.7 | 75.4 | 78.7 |
| 14.0 | 58.7 | 62.9 | 70.6 | 80.2 | 90.9 | 101.8 | 108.8 |  | 49.4 | 52.6 | 58.3 | 64.9 | 71.5 | 77.3 | 80.7 |
| 15.0 | 60.6 | 65.0 | 72.9 | 82.8 | 93.9 | 105.1 | 112.4 |  | 49.7 | 53.0 | 58.6 | 65.2 | 71.9 | 77.8 | 81.2 |
| 16.0 | 62.0 | 66.4 | 74.6 | 84.7 | 96.1 | 107.5 | 114.9 |  | 49.3 | 52.5 | 58.2 | 64.7 | 71.4 | 77.2 | 80.6 |
| 17.0 | 62.9 | 67.4 | 75.7 | 86.0 | 97.5 | 109.1 | 116.7 |  | 48.6 | 51.7 | 57.3 | 63.7 | 70.3 | 76.0 | 79.3 |
| 18.0 | 63.6 | 68.1 | 76.5 | 86.9 | 98.5 | 110.3 | 117.9 |  | 47.6 | 50.7 | 56.1 | 62.5 | 68.9 | 74.5 | 77.8 |

LVM: Left ventricular mass

**Supplementary Table S23. Centiles of RVEDV with exclusion of trabeculations and papillary muscles in volume by age and sex.**

| Age | Boys | | | | | | |  | Girls | | | | | | |
| --- | --- | --- | --- | --- | --- | --- | --- | --- | --- | --- | --- | --- | --- | --- | --- |
|  | 5th | 10th | 25th | 50th | 75th | 90th | 95th |  | 5th | 10th | 25th | 50th | 75th | 90th | 95th |
| 4.0 | 36.6 | 39.1 | 43.5 | 49.0 | 55.1 | 61.2 | 65.2 |  | 28.3 | 30.0 | 33.1 | 37.1 | 41.3 | 44.8 | 46.8 |
| 5.0 | 38.7 | 41.2 | 45.9 | 51.7 | 58.1 | 64.6 | 68.8 |  | 36.1 | 38.2 | 42.2 | 47.3 | 52.6 | 57.1 | 59.7 |
| 6.0 | 41.2 | 44.0 | 48.9 | 55.1 | 62.0 | 68.9 | 73.4 |  | 43.1 | 45.7 | 50.4 | 56.5 | 62.9 | 68.2 | 71.3 |
| 7.0 | 44.8 | 47.8 | 53.2 | 59.9 | 67.4 | 74.9 | 79.8 |  | 48.8 | 51.7 | 57.0 | 63.9 | 71.1 | 77.2 | 80.6 |
| 8.0 | 50.4 | 53.7 | 59.8 | 67.4 | 75.8 | 84.2 | 89.7 |  | 53.6 | 56.7 | 62.6 | 70.2 | 78.1 | 84.7 | 88.5 |
| 9.0 | 56.8 | 60.6 | 67.5 | 76.0 | 85.5 | 95.0 | 101.2 |  | 57.5 | 60.8 | 67.1 | 75.3 | 83.8 | 90.9 | 94.9 |
| 10.0 | 64.4 | 68.6 | 76.4 | 86.0 | 96.8 | 107.6 | 114.6 |  | 62.3 | 65.9 | 72.7 | 81.5 | 90.7 | 98.5 | 102.9 |
| 11.0 | 72.7 | 77.5 | 86.3 | 97.1 | 109.3 | 121.5 | 129.4 |  | 68.2 | 72.2 | 79.7 | 89.4 | 99.5 | 107.9 | 112.8 |
| 12.0 | 81.0 | 86.4 | 96.2 | 108.3 | 121.9 | 135.4 | 144.2 |  | 73.3 | 77.6 | 85.7 | 96.1 | 106.9 | 116.0 | 121.2 |
| 13.0 | 88.1 | 93.9 | 104.6 | 117.7 | 132.5 | 147.2 | 156.8 |  | 77.2 | 81.7 | 90.2 | 101.1 | 112.5 | 122.1 | 127.5 |
| 14.0 | 93.2 | 99.4 | 110.6 | 124.5 | 140.1 | 155.7 | 165.8 |  | 79.9 | 84.6 | 93.3 | 104.6 | 116.5 | 126.4 | 132.0 |
| 15.0 | 96.8 | 103.2 | 114.9 | 129.4 | 145.5 | 161.7 | 172.3 |  | 81.4 | 86.2 | 95.1 | 106.7 | 118.7 | 128.8 | 134.6 |
| 16.0 | 99.1 | 105.7 | 117.7 | 132.5 | 149.1 | 165.7 | 176.4 |  | 82.2 | 87.0 | 96.0 | 107.6 | 119.8 | 130.0 | 135.8 |
| 17.0 | 100.2 | 106.9 | 119.0 | 133.9 | 150.7 | 167.5 | 178.4 |  | 82.5 | 87.4 | 96.4 | 108.1 | 120.3 | 130.6 | 136.4 |
| 18.0 | 100.6 | 107.3 | 119.4 | 134.4 | 151.2 | 168.1 | 179.0 |  | 82.7 | 87.6 | 96.6 | 108.3 | 120.6 | 130.8 | 136.7 |

RVEDV: Right ventricular end-diastolic volume

**Supplementary Table S24. Centiles of RVESV with exclusion of trabeculations and papillary muscles in volume by age and sex.**

| Age | Boys | | | | | | |  | Girls | | | | | | |
| --- | --- | --- | --- | --- | --- | --- | --- | --- | --- | --- | --- | --- | --- | --- | --- |
|  | 5th | 10th | 25th | 50th | 75th | 90th | 95th |  | 5th | 10th | 25th | 50th | 75th | 90th | 95th |
| 4.0 | 10.9 | 12.4 | 14.9 | 17.9 | 21.1 | 24.2 | 26.0 |  | 6.8 | 7.5 | 8.8 | 10.4 | 12.0 | 13.3 | 14.1 |
| 5.0 | 11.4 | 12.9 | 15.6 | 18.7 | 22.1 | 25.2 | 27.2 |  | 9.8 | 10.8 | 12.6 | 14.8 | 17.1 | 19.1 | 20.2 |
| 6.0 | 12.0 | 13.6 | 16.4 | 19.8 | 23.3 | 26.6 | 28.7 |  | 12.4 | 13.7 | 15.9 | 18.8 | 21.7 | 24.2 | 25.6 |
| 7.0 | 12.9 | 14.6 | 17.6 | 21.2 | 25.0 | 28.5 | 30.7 |  | 14.4 | 15.8 | 18.5 | 21.7 | 25.1 | 28.0 | 29.7 |
| 8.0 | 14.4 | 16.3 | 19.6 | 23.6 | 27.8 | 31.8 | 34.2 |  | 16.1 | 17.7 | 20.6 | 24.3 | 28.1 | 31.4 | 33.2 |
| 9.0 | 16.3 | 18.4 | 22.2 | 26.7 | 31.5 | 36.0 | 38.8 |  | 17.8 | 19.6 | 22.8 | 26.9 | 31.1 | 34.6 | 36.7 |
| 10.0 | 18.9 | 21.4 | 25.8 | 31.0 | 36.6 | 41.8 | 45.1 |  | 19.7 | 21.7 | 25.3 | 29.9 | 34.5 | 38.5 | 40.8 |
| 11.0 | 22.0 | 24.9 | 30.1 | 36.2 | 42.6 | 48.7 | 52.5 |  | 21.9 | 24.1 | 28.1 | 33.1 | 38.2 | 42.6 | 45.1 |
| 12.0 | 25.3 | 28.6 | 34.5 | 41.5 | 48.9 | 55.9 | 60.3 |  | 23.6 | 26.0 | 30.3 | 35.8 | 41.3 | 46.1 | 48.8 |
| 13.0 | 28.1 | 31.9 | 38.4 | 46.2 | 54.4 | 62.2 | 67.1 |  | 25.1 | 27.7 | 32.3 | 38.0 | 43.9 | 49.0 | 51.9 |
| 14.0 | 30.3 | 34.3 | 41.4 | 49.8 | 58.6 | 67.0 | 72.2 |  | 26.4 | 29.0 | 33.8 | 39.9 | 46.1 | 51.4 | 54.4 |
| 15.0 | 32.0 | 36.2 | 43.7 | 52.6 | 62.0 | 70.8 | 76.3 |  | 27.1 | 29.8 | 34.8 | 41.0 | 47.4 | 52.8 | 55.9 |
| 16.0 | 33.4 | 37.8 | 45.6 | 54.8 | 64.6 | 73.8 | 79.6 |  | 27.4 | 30.2 | 35.2 | 41.5 | 47.9 | 53.4 | 56.6 |
| 17.0 | 34.3 | 38.9 | 46.9 | 56.4 | 66.4 | 75.9 | 81.8 |  | 27.5 | 30.3 | 35.3 | 41.6 | 48.1 | 53.6 | 56.8 |
| 18.0 | 35.0 | 39.6 | 47.8 | 57.5 | 67.8 | 77.5 | 83.5 |  | 27.5 | 30.2 | 35.3 | 41.5 | 48.0 | 53.5 | 56.7 |

RVESV: Right ventricular end-systolic volume

**Supplementary Table S25. Centiles of RVSV with exclusion of trabeculations and papillary muscles in volume by age and sex.**

| Age | Boys | | | | | | |  | Girls | | | | | | |
| --- | --- | --- | --- | --- | --- | --- | --- | --- | --- | --- | --- | --- | --- | --- | --- |
|  | 5th | 10th | 25th | 50th | 75th | 90th | 95th |  | 5th | 10th | 25th | 50th | 75th | 90th | 95th |
| 4.0 | 23.6 | 24.8 | 27.2 | 30.1 | 33.6 | 37.3 | 39.9 |  | 18.9 | 20.3 | 22.5 | 24.8 | 27.1 | 29.6 | 31.3 |
| 5.0 | 25.4 | 26.7 | 29.2 | 32.4 | 36.2 | 40.2 | 42.9 |  | 23.8 | 25.6 | 28.3 | 31.2 | 34.2 | 37.3 | 39.4 |
| 6.0 | 27.6 | 29.1 | 31.8 | 35.3 | 39.4 | 43.7 | 46.6 |  | 28.3 | 30.3 | 33.6 | 37.0 | 40.6 | 44.3 | 46.7 |
| 7.0 | 30.5 | 32.1 | 35.1 | 39.0 | 43.5 | 48.3 | 51.5 |  | 31.9 | 34.3 | 38.0 | 41.8 | 45.8 | 50.0 | 52.8 |
| 8.0 | 34.3 | 36.2 | 39.5 | 43.9 | 49.0 | 54.3 | 58.0 |  | 35.0 | 37.5 | 41.6 | 45.8 | 50.2 | 54.8 | 57.9 |
| 9.0 | 38.3 | 40.4 | 44.1 | 49.0 | 54.7 | 60.7 | 64.7 |  | 37.3 | 40.0 | 44.3 | 48.8 | 53.5 | 58.4 | 61.7 |
| 10.0 | 42.7 | 44.9 | 49.1 | 54.5 | 60.8 | 67.5 | 72.1 |  | 40.0 | 42.9 | 47.6 | 52.4 | 57.4 | 62.7 | 66.2 |
| 11.0 | 47.4 | 49.9 | 54.5 | 60.5 | 67.5 | 75.0 | 80.0 |  | 43.6 | 46.8 | 51.9 | 57.2 | 62.6 | 68.4 | 72.2 |
| 12.0 | 52.1 | 54.8 | 59.9 | 66.5 | 74.2 | 82.3 | 87.9 |  | 46.8 | 50.1 | 55.6 | 61.2 | 67.1 | 73.2 | 77.3 |
| 13.0 | 55.9 | 58.9 | 64.4 | 71.4 | 79.7 | 88.5 | 94.4 |  | 48.9 | 52.4 | 58.1 | 64.0 | 70.1 | 76.5 | 80.8 |
| 14.0 | 58.7 | 61.8 | 67.6 | 75.0 | 83.7 | 92.9 | 99.1 |  | 50.1 | 53.8 | 59.6 | 65.7 | 71.9 | 78.5 | 82.9 |
| 15.0 | 60.5 | 63.7 | 69.6 | 77.2 | 86.2 | 95.7 | 102.1 |  | 50.7 | 54.3 | 60.3 | 66.4 | 72.7 | 79.4 | 83.8 |
| 16.0 | 61.5 | 64.7 | 70.8 | 78.5 | 87.6 | 97.3 | 103.8 |  | 50.7 | 54.4 | 60.3 | 66.4 | 72.8 | 79.5 | 83.9 |
| 17.0 | 61.9 | 65.2 | 71.3 | 79.1 | 88.3 | 98.0 | 104.6 |  | 50.5 | 54.2 | 60.1 | 66.2 | 72.5 | 79.1 | 83.6 |
| 18.0 | 62.0 | 65.3 | 71.4 | 79.2 | 88.4 | 98.1 | 104.8 |  | 50.1 | 53.7 | 59.6 | 65.6 | 71.9 | 78.5 | 82.9 |

RVSV: Right ventricular stroke volume

**Supplementary Table S26. Centiles of LAV_min_ by BSA and sex.**

| BSA | Boys | | | | | | |  | Girls | | | | | | |
| --- | --- | --- | --- | --- | --- | --- | --- | --- | --- | --- | --- | --- | --- | --- | --- |
|  | 5th | 10th | 25th | 50th | 75th | 90th | 95th |  | 5th | 10th | 25th | 50th | 75th | 90th | 95th |
| 0.6 | 2.3 | 2.7 | 3.4 | 4.2 | 5.1 | 5.9 | 6.4 |  | 2.6 | 2.9 | 3.5 | 4.2 | 5.0 | 5.7 | 6.2 |
| 0.7 | 3.1 | 3.6 | 4.5 | 5.6 | 6.7 | 7.7 | 8.4 |  | 3.2 | 3.6 | 4.3 | 5.2 | 6.1 | 7.1 | 7.7 |
| 0.8 | 3.9 | 4.5 | 5.6 | 6.9 | 8.3 | 9.7 | 10.5 |  | 3.8 | 4.2 | 5.1 | 6.1 | 7.3 | 8.4 | 9.1 |
| 0.9 | 4.5 | 5.2 | 6.5 | 8.1 | 9.7 | 11.3 | 12.2 |  | 4.5 | 5.0 | 6.0 | 7.2 | 8.6 | 9.9 | 10.8 |
| 1.0 | 5.0 | 5.9 | 7.3 | 9.0 | 10.9 | 12.6 | 13.7 |  | 5.3 | 6.0 | 7.2 | 8.6 | 10.2 | 11.8 | 12.9 |
| 1.1 | 5.7 | 6.6 | 8.2 | 10.2 | 12.2 | 14.2 | 15.4 |  | 6.1 | 6.8 | 8.2 | 9.8 | 11.7 | 13.5 | 14.7 |
| 1.2 | 6.3 | 7.4 | 9.2 | 11.4 | 13.7 | 15.9 | 17.2 |  | 6.6 | 7.4 | 8.9 | 10.7 | 12.7 | 14.7 | 16.0 |
| 1.3 | 7.1 | 8.2 | 10.3 | 12.7 | 15.3 | 17.7 | 19.2 |  | 7.2 | 8.1 | 9.7 | 11.7 | 13.9 | 16.1 | 17.5 |
| 1.4 | 7.9 | 9.2 | 11.5 | 14.2 | 17.1 | 19.8 | 21.5 |  | 8.0 | 9.0 | 10.8 | 13.0 | 15.4 | 17.8 | 19.4 |
| 1.5 | 8.8 | 10.2 | 12.8 | 15.8 | 19.0 | 22.0 | 23.9 |  | 8.8 | 9.9 | 11.8 | 14.2 | 16.9 | 19.6 | 21.3 |
| 1.6 | 9.6 | 11.2 | 14.0 | 17.3 | 20.8 | 24.1 | 26.2 |  | 9.6 | 10.7 | 12.8 | 15.4 | 18.3 | 21.2 | 23.1 |
| 1.7 | 10.2 | 11.9 | 14.8 | 18.3 | 22.0 | 25.5 | 27.7 |  | 10.2 | 11.5 | 13.7 | 16.5 | 19.7 | 22.7 | 24.7 |
| 1.8 | 10.4 | 12.1 | 15.1 | 18.7 | 22.5 | 26.1 | 28.3 |  | 10.9 | 12.2 | 14.6 | 17.6 | 21.0 | 24.2 | 26.4 |
| 1.9 | 10.6 | 12.3 | 15.4 | 19.0 | 22.8 | 26.5 | 28.7 |  | 11.6 | 13.0 | 15.5 | 18.7 | 22.2 | 25.8 | 28.0 |

BSA: Body surface area; LAV_min_: Minimal left atrial volume

**Supplementary Table S27. Centiles of LAV_max_ by BSA and sex.**

| BSA | Boys | | | | | | |  | Girls | | | | | | |
| --- | --- | --- | --- | --- | --- | --- | --- | --- | --- | --- | --- | --- | --- | --- | --- |
|  | 5th | 10th | 25th | 50th | 75th | 90th | 95th |  | 5th | 10th | 25th | 50th | 75th | 90th | 95th |
| 0.6 | 9.2 | 10.0 | 11.7 | 14.1 | 16.7 | 18.7 | 19.8 |  | 7.5 | 8.6 | 10.3 | 12.3 | 14.3 | 16.0 | 17.1 |
| 0.7 | 11.7 | 12.8 | 14.9 | 18.0 | 21.2 | 23.8 | 25.2 |  | 10.0 | 11.4 | 13.7 | 16.4 | 19.0 | 21.3 | 22.7 |
| 0.8 | 14.1 | 15.4 | 18.0 | 21.6 | 25.5 | 28.7 | 30.4 |  | 12.1 | 13.9 | 16.7 | 19.9 | 23.1 | 26.0 | 27.7 |
| 0.9 | 16.1 | 17.6 | 20.6 | 24.8 | 29.3 | 32.8 | 34.8 |  | 14.0 | 15.9 | 19.3 | 22.9 | 26.6 | 29.9 | 31.9 |
| 1.0 | 17.9 | 19.6 | 22.9 | 27.5 | 32.5 | 36.5 | 38.6 |  | 15.7 | 18.0 | 21.7 | 25.8 | 30.0 | 33.7 | 35.9 |
| 1.1 | 19.6 | 21.4 | 25.0 | 30.1 | 35.5 | 39.8 | 42.2 |  | 17.4 | 19.9 | 24.0 | 28.6 | 33.2 | 37.3 | 39.7 |
| 1.2 | 21.2 | 23.2 | 27.1 | 32.6 | 38.5 | 43.2 | 45.7 |  | 18.9 | 21.6 | 26.0 | 31.0 | 36.0 | 40.4 | 43.1 |
| 1.3 | 22.9 | 25.0 | 29.2 | 35.2 | 41.6 | 46.6 | 49.4 |  | 20.4 | 23.3 | 28.1 | 33.5 | 38.8 | 43.7 | 46.5 |
| 1.4 | 24.8 | 27.0 | 31.6 | 38.0 | 44.9 | 50.4 | 53.4 |  | 22.0 | 25.2 | 30.4 | 36.2 | 42.0 | 47.2 | 50.3 |
| 1.5 | 26.8 | 29.2 | 34.2 | 41.1 | 48.6 | 54.5 | 57.7 |  | 23.8 | 27.1 | 32.8 | 39.0 | 45.3 | 50.9 | 54.3 |
| 1.6 | 29.0 | 31.6 | 37.0 | 44.5 | 52.6 | 59.0 | 62.4 |  | 25.4 | 29.0 | 35.1 | 41.7 | 48.4 | 54.4 | 58.0 |
| 1.7 | 31.0 | 33.8 | 39.5 | 47.6 | 56.2 | 63.0 | 66.8 |  | 26.9 | 30.7 | 37.1 | 44.2 | 51.2 | 57.6 | 61.4 |
| 1.8 | 32.7 | 35.7 | 41.8 | 50.3 | 59.4 | 66.6 | 70.5 |  | 28.3 | 32.3 | 39.1 | 46.5 | 53.9 | 60.6 | 64.6 |
| 1.9 | 34.4 | 37.6 | 43.9 | 52.8 | 62.4 | 70.0 | 74.1 |  | 29.7 | 34.0 | 41.0 | 48.9 | 56.7 | 63.7 | 67.9 |

BSA: Body surface area; LAV_max_: Maximal left atrial volume

**Supplementary Table S28. Centiles of RAV_min_ by BSA and sex.**

| BSA | Boys | | | | | | |  | Girls | | | | | | |
| --- | --- | --- | --- | --- | --- | --- | --- | --- | --- | --- | --- | --- | --- | --- | --- |
|  | 5th | 10th | 25th | 50th | 75th | 90th | 95th |  | 5th | 10th | 25th | 50th | 75th | 90th | 95th |
| 0.6 | 3.8 | 4.3 | 5.2 | 6.3 | 7.5 | 8.7 | 9.5 |  | 2.7 | 3.0 | 3.6 | 4.2 | 5.0 | 5.7 | 6.2 |
| 0.7 | 5.0 | 5.6 | 6.7 | 8.1 | 9.7 | 11.2 | 12.2 |  | 4.4 | 4.9 | 5.8 | 6.9 | 8.1 | 9.3 | 10.0 |
| 0.8 | 6.2 | 6.9 | 8.3 | 10.1 | 12.0 | 13.9 | 15.2 |  | 5.8 | 6.5 | 7.7 | 9.1 | 10.7 | 12.3 | 13.3 |
| 0.9 | 7.6 | 8.5 | 10.3 | 12.4 | 14.8 | 17.2 | 18.7 |  | 6.9 | 7.7 | 9.1 | 10.8 | 12.7 | 14.5 | 15.7 |
| 1.0 | 9.0 | 10.1 | 12.1 | 14.6 | 17.4 | 20.2 | 22.0 |  | 8.2 | 9.1 | 10.8 | 12.9 | 15.1 | 17.3 | 18.7 |
| 1.1 | 10.2 | 11.4 | 13.7 | 16.6 | 19.7 | 22.9 | 25.0 |  | 9.9 | 11.0 | 13.0 | 15.5 | 18.2 | 20.9 | 22.6 |
| 1.2 | 11.4 | 12.8 | 15.4 | 18.6 | 22.2 | 25.8 | 28.0 |  | 11.3 | 12.6 | 14.9 | 17.7 | 20.8 | 23.9 | 25.8 |
| 1.3 | 12.6 | 14.1 | 17.0 | 20.5 | 24.4 | 28.3 | 30.9 |  | 12.2 | 13.6 | 16.0 | 19.1 | 22.5 | 25.7 | 27.8 |
| 1.4 | 13.8 | 15.5 | 18.7 | 22.5 | 26.9 | 31.2 | 34.0 |  | 13.2 | 14.6 | 17.3 | 20.6 | 24.2 | 27.8 | 30.1 |
| 1.5 | 15.4 | 17.3 | 20.7 | 25.0 | 29.9 | 34.7 | 37.8 |  | 14.3 | 15.9 | 18.8 | 22.3 | 26.3 | 30.1 | 32.6 |
| 1.6 | 17.1 | 19.3 | 23.1 | 27.9 | 33.3 | 38.7 | 42.1 |  | 15.6 | 17.4 | 20.6 | 24.5 | 28.8 | 33.0 | 35.7 |
| 1.7 | 18.8 | 21.1 | 25.3 | 30.6 | 36.5 | 42.3 | 46.1 |  | 17.2 | 19.1 | 22.7 | 27.0 | 31.7 | 36.3 | 39.3 |
| 1.8 | 20.1 | 22.5 | 27.1 | 32.7 | 39.0 | 45.2 | 49.3 |  | 18.8 | 20.9 | 24.8 | 29.5 | 34.6 | 39.7 | 43.0 |
| 1.9 | 21.2 | 23.8 | 28.6 | 34.6 | 41.2 | 47.9 | 52.1 |  | 20.4 | 22.7 | 26.9 | 32.0 | 37.6 | 43.1 | 46.6 |

BSA: Body surface area; RAV_min_: Minimal right atrial volume

**Supplementary Table S29. Centiles of RAV_max_ by BSA and sex.**

| BSA | Boys | | | | | | |  | Girls | | | | | | |
| --- | --- | --- | --- | --- | --- | --- | --- | --- | --- | --- | --- | --- | --- | --- | --- |
|  | 5th | 10th | 25th | 50th | 75th | 90th | 95th |  | 5th | 10th | 25th | 50th | 75th | 90th | 95th |
| 0.6 | 11.8 | 13.0 | 14.9 | 17.3 | 19.9 | 22.4 | 24.0 |  | 7.7 | 8.4 | 9.7 | 11.2 | 12.7 | 14.2 | 15.2 |
| 0.7 | 14.2 | 15.5 | 17.9 | 20.8 | 23.9 | 26.8 | 28.7 |  | 11.8 | 12.9 | 14.9 | 17.1 | 19.5 | 21.8 | 23.3 |
| 0.8 | 16.4 | 17.9 | 20.7 | 24.0 | 27.5 | 31.0 | 33.1 |  | 15.3 | 16.7 | 19.2 | 22.1 | 25.2 | 28.2 | 30.0 |
| 0.9 | 18.6 | 20.4 | 23.5 | 27.2 | 31.3 | 35.2 | 37.7 |  | 17.7 | 19.3 | 22.2 | 25.6 | 29.2 | 32.6 | 34.7 |
| 1.0 | 21.0 | 23.0 | 26.5 | 30.8 | 35.3 | 39.8 | 42.6 |  | 19.8 | 21.6 | 24.8 | 28.6 | 32.6 | 36.4 | 38.8 |
| 1.1 | 23.4 | 25.6 | 29.5 | 34.3 | 39.4 | 44.3 | 47.4 |  | 21.9 | 23.9 | 27.4 | 31.6 | 36.1 | 40.3 | 42.9 |
| 1.2 | 25.9 | 28.4 | 32.7 | 37.9 | 43.6 | 49.0 | 52.4 |  | 23.3 | 25.5 | 29.3 | 33.8 | 38.5 | 43.0 | 45.9 |
| 1.3 | 28.3 | 30.9 | 35.6 | 41.3 | 47.5 | 53.4 | 57.2 |  | 24.3 | 26.5 | 30.4 | 35.1 | 40.0 | 44.7 | 47.6 |
| 1.4 | 30.8 | 33.7 | 38.9 | 45.1 | 51.8 | 58.3 | 62.3 |  | 25.9 | 28.3 | 32.5 | 37.4 | 42.7 | 47.7 | 50.8 |
| 1.5 | 33.5 | 36.7 | 42.3 | 49.1 | 56.4 | 63.4 | 67.9 |  | 28.1 | 30.7 | 35.2 | 40.6 | 46.3 | 51.8 | 55.2 |
| 1.6 | 36.0 | 39.4 | 45.5 | 52.7 | 60.6 | 68.2 | 72.9 |  | 30.5 | 33.3 | 38.2 | 44.0 | 50.2 | 56.1 | 59.8 |
| 1.7 | 38.0 | 41.6 | 47.9 | 55.6 | 63.9 | 71.8 | 76.9 |  | 32.9 | 35.9 | 41.2 | 47.5 | 54.2 | 60.6 | 64.6 |
| 1.8 | 39.5 | 43.2 | 49.8 | 57.7 | 66.3 | 74.6 | 79.8 |  | 35.3 | 38.5 | 44.2 | 51.0 | 58.2 | 65.0 | 69.3 |
| 1.9 | 40.8 | 44.6 | 51.5 | 59.7 | 68.6 | 77.2 | 82.6 |  | 37.7 | 41.1 | 47.2 | 54.5 | 62.2 | 69.5 | 74.0 |

BSA: Body surface area; RAV_max_: Maximal right atrial volume

**Supplementary Table S30. Centiles of LAV_min_ by age and sex.**

| Age | Boys | | | | | | |  | Girls | | | | | | |
| --- | --- | --- | --- | --- | --- | --- | --- | --- | --- | --- | --- | --- | --- | --- | --- |
|  | 5th | 10th | 25th | 50th | 75th | 90th | 95th |  | 5th | 10th | 25th | 50th | 75th | 90th | 95th |
| 4.0 | 3.3 | 3.8 | 4.7 | 6.0 | 7.5 | 8.6 | 9.3 |  | 2.8 | 3.2 | 4.0 | 4.9 | 6.0 | 6.9 | 7.4 |
| 5.0 | 3.6 | 4.1 | 5.2 | 6.6 | 8.1 | 9.4 | 10.1 |  | 3.6 | 4.1 | 5.0 | 6.3 | 7.6 | 8.7 | 9.3 |
| 6.0 | 3.9 | 4.5 | 5.6 | 7.1 | 8.8 | 10.1 | 10.9 |  | 4.2 | 4.8 | 5.9 | 7.4 | 8.9 | 10.2 | 11.0 |
| 7.0 | 4.4 | 5.0 | 6.2 | 7.9 | 9.8 | 11.3 | 12.1 |  | 4.7 | 5.4 | 6.7 | 8.3 | 10.0 | 11.5 | 12.4 |
| 8.0 | 5.0 | 5.7 | 7.0 | 9.0 | 11.1 | 12.8 | 13.7 |  | 5.2 | 5.9 | 7.2 | 9.0 | 10.9 | 12.5 | 13.4 |
| 9.0 | 5.6 | 6.4 | 7.9 | 10.1 | 12.5 | 14.4 | 15.5 |  | 5.5 | 6.3 | 7.7 | 9.6 | 11.7 | 13.4 | 14.4 |
| 10.0 | 6.1 | 7.0 | 8.7 | 11.1 | 13.7 | 15.8 | 17.0 |  | 6.0 | 6.8 | 8.4 | 10.4 | 12.6 | 14.5 | 15.6 |
| 11.0 | 6.7 | 7.7 | 9.6 | 12.2 | 15.0 | 17.4 | 18.7 |  | 6.6 | 7.5 | 9.3 | 11.5 | 14.0 | 16.0 | 17.3 |
| 12.0 | 7.6 | 8.6 | 10.8 | 13.7 | 16.9 | 19.5 | 21.0 |  | 7.4 | 8.4 | 10.4 | 12.9 | 15.6 | 17.9 | 19.3 |
| 13.0 | 8.5 | 9.7 | 12.1 | 15.5 | 19.1 | 22.0 | 23.7 |  | 8.1 | 9.2 | 11.3 | 14.1 | 17.1 | 19.6 | 21.1 |
| 14.0 | 9.4 | 10.7 | 13.4 | 17.0 | 21.0 | 24.3 | 26.1 |  | 8.5 | 9.6 | 11.9 | 14.8 | 17.9 | 20.6 | 22.1 |
| 15.0 | 10.2 | 11.6 | 14.4 | 18.4 | 22.7 | 26.2 | 28.2 |  | 8.6 | 9.8 | 12.1 | 15.1 | 18.2 | 21.0 | 22.5 |
| 16.0 | 10.8 | 12.3 | 15.4 | 19.6 | 24.2 | 27.9 | 30.0 |  | 8.7 | 9.9 | 12.2 | 15.1 | 18.3 | 21.1 | 22.6 |
| 17.0 | 11.4 | 13.1 | 16.3 | 20.7 | 25.5 | 29.5 | 31.7 |  | 8.6 | 9.8 | 12.1 | 15.1 | 18.3 | 21.0 | 22.6 |
| 18.0 | 12.0 | 13.7 | 17.1 | 21.8 | 26.9 | 31.0 | 33.4 |  | 8.6 | 9.8 | 12.1 | 15.0 | 18.2 | 20.9 | 22.4 |

LAV_min_: Minimal left atrial volume

**Supplementary Table S31. Centiles of LAV_max_ by age and sex.**

| Age | Boys | | | | | | |  | Girls | | | | | | |
| --- | --- | --- | --- | --- | --- | --- | --- | --- | --- | --- | --- | --- | --- | --- | --- |
|  | 5th | 10th | 25th | 50th | 75th | 90th | 95th |  | 5th | 10th | 25th | 50th | 75th | 90th | 95th |
| 4.0 | 14.0 | 15.7 | 18.7 | 22.2 | 25.9 | 29.4 | 31.6 |  | 8.9 | 10.1 | 12.4 | 15.7 | 18.8 | 20.9 | 21.9 |
| 5.0 | 14.0 | 15.7 | 18.6 | 22.1 | 25.8 | 29.2 | 31.4 |  | 11.0 | 12.4 | 15.3 | 19.3 | 23.2 | 25.8 | 27.0 |
| 6.0 | 14.2 | 15.9 | 19.0 | 22.5 | 26.3 | 29.8 | 31.9 |  | 12.9 | 14.6 | 18.0 | 22.7 | 27.2 | 30.2 | 31.6 |
| 7.0 | 15.1 | 17.0 | 20.2 | 24.0 | 28.0 | 31.7 | 34.0 |  | 14.5 | 16.4 | 20.2 | 25.5 | 30.6 | 34.0 | 35.6 |
| 8.0 | 16.7 | 18.7 | 22.3 | 26.5 | 30.9 | 35.0 | 37.5 |  | 15.6 | 17.7 | 21.8 | 27.6 | 33.1 | 36.7 | 38.4 |
| 9.0 | 18.4 | 20.7 | 24.6 | 29.2 | 34.1 | 38.6 | 41.4 |  | 16.5 | 18.6 | 23.0 | 29.0 | 34.8 | 38.7 | 40.5 |
| 10.0 | 20.4 | 22.9 | 27.2 | 32.3 | 37.7 | 42.8 | 45.9 |  | 17.4 | 19.7 | 24.3 | 30.7 | 36.8 | 40.9 | 42.8 |
| 11.0 | 22.5 | 25.2 | 30.0 | 35.7 | 41.6 | 47.1 | 50.6 |  | 18.8 | 21.2 | 26.2 | 33.1 | 39.7 | 44.1 | 46.1 |
| 12.0 | 24.8 | 27.8 | 33.1 | 39.3 | 45.8 | 51.9 | 55.7 |  | 20.2 | 22.8 | 28.1 | 35.5 | 42.6 | 47.4 | 49.6 |
| 13.0 | 27.0 | 30.3 | 36.0 | 42.8 | 49.9 | 56.6 | 60.7 |  | 21.6 | 24.4 | 30.1 | 37.9 | 45.5 | 50.6 | 52.9 |
| 14.0 | 28.7 | 32.2 | 38.3 | 45.5 | 53.0 | 60.1 | 64.5 |  | 22.7 | 25.7 | 31.7 | 40.0 | 48.0 | 53.4 | 55.9 |
| 15.0 | 29.8 | 33.4 | 39.7 | 47.2 | 55.0 | 62.4 | 66.9 |  | 23.7 | 26.7 | 33.0 | 41.7 | 50.0 | 55.5 | 58.1 |
| 16.0 | 30.5 | 34.3 | 40.8 | 48.4 | 56.4 | 64.0 | 68.7 |  | 24.4 | 27.6 | 34.1 | 43.0 | 51.6 | 57.3 | 60.0 |
| 17.0 | 31.2 | 35.0 | 41.6 | 49.4 | 57.6 | 65.4 | 70.1 |  | 25.1 | 28.4 | 35.0 | 44.2 | 53.0 | 58.9 | 61.7 |
| 18.0 | 31.8 | 35.6 | 42.4 | 50.3 | 58.7 | 66.6 | 71.4 |  | 25.7 | 29.1 | 35.9 | 45.3 | 54.3 | 60.4 | 63.2 |

LAV_max_: Maximal left atrial volume

**Supplementary Table S32. Centiles of RAV_min_ by age and sex.**

| Age | Boys | | | | | | |  | Girls | | | | | | |
| --- | --- | --- | --- | --- | --- | --- | --- | --- | --- | --- | --- | --- | --- | --- | --- |
|  | 5th | 10th | 25th | 50th | 75th | 90th | 95th |  | 5th | 10th | 25th | 50th | 75th | 90th | 95th |
| 4.0 | 4.7 | 5.3 | 6.5 | 8.0 | 9.8 | 11.7 | 13.0 |  | 3.2 | 3.5 | 4.2 | 5.2 | 6.4 | 7.5 | 8.2 |
| 5.0 | 5.8 | 6.6 | 8.0 | 9.8 | 12.1 | 14.4 | 16.0 |  | 4.9 | 5.4 | 6.4 | 8.0 | 9.8 | 11.5 | 12.6 |
| 6.0 | 6.9 | 7.8 | 9.4 | 11.6 | 14.2 | 17.0 | 18.9 |  | 6.3 | 7.0 | 8.3 | 10.3 | 12.6 | 14.9 | 16.2 |
| 7.0 | 7.6 | 8.6 | 10.4 | 12.9 | 15.8 | 18.8 | 20.9 |  | 7.4 | 8.1 | 9.7 | 12.0 | 14.8 | 17.4 | 19.0 |
| 8.0 | 8.4 | 9.5 | 11.5 | 14.2 | 17.4 | 20.8 | 23.0 |  | 8.4 | 9.3 | 11.1 | 13.7 | 16.9 | 19.9 | 21.7 |
| 9.0 | 9.3 | 10.4 | 12.7 | 15.6 | 19.1 | 22.9 | 25.4 |  | 9.3 | 10.3 | 12.3 | 15.2 | 18.7 | 22.0 | 24.1 |
| 10.0 | 10.3 | 11.6 | 14.1 | 17.4 | 21.3 | 25.4 | 28.2 |  | 10.4 | 11.4 | 13.6 | 16.8 | 20.7 | 24.4 | 26.7 |
| 11.0 | 11.7 | 13.1 | 15.9 | 19.6 | 24.1 | 28.8 | 31.9 |  | 11.5 | 12.7 | 15.2 | 18.8 | 23.1 | 27.2 | 29.7 |
| 12.0 | 13.4 | 15.1 | 18.3 | 22.6 | 27.6 | 33.0 | 36.6 |  | 12.6 | 13.9 | 16.5 | 20.4 | 25.1 | 29.6 | 32.3 |
| 13.0 | 15.2 | 17.1 | 20.8 | 25.6 | 31.4 | 37.5 | 41.6 |  | 13.4 | 14.8 | 17.6 | 21.8 | 26.8 | 31.5 | 34.4 |
| 14.0 | 16.7 | 18.8 | 22.8 | 28.1 | 34.5 | 41.2 | 45.7 |  | 13.9 | 15.4 | 18.3 | 22.6 | 27.9 | 32.8 | 35.8 |
| 15.0 | 17.8 | 20.1 | 24.4 | 30.1 | 36.8 | 44.0 | 48.8 |  | 14.1 | 15.5 | 18.5 | 22.9 | 28.2 | 33.1 | 36.2 |
| 16.0 | 18.7 | 21.0 | 25.5 | 31.5 | 38.6 | 46.1 | 51.1 |  | 13.9 | 15.4 | 18.3 | 22.7 | 27.9 | 32.8 | 35.9 |
| 17.0 | 19.2 | 21.7 | 26.3 | 32.5 | 39.8 | 47.5 | 52.7 |  | 13.7 | 15.1 | 18.0 | 22.3 | 27.4 | 32.3 | 35.3 |
| 18.0 | 19.7 | 22.2 | 26.9 | 33.2 | 40.6 | 48.5 | 53.9 |  | 13.5 | 14.8 | 17.7 | 21.9 | 26.9 | 31.7 | 34.6 |

RAV_min_: Minimal right atrial volume

**Supplementary Table S33. Centiles of RAV_max_ by age and sex.**

| Age | Boys | | | | | | |  | Girls | | | | | | |
| --- | --- | --- | --- | --- | --- | --- | --- | --- | --- | --- | --- | --- | --- | --- | --- |
|  | 5th | 10th | 25th | 50th | 75th | 90th | 95th |  | 5th | 10th | 25th | 50th | 75th | 90th | 95th |
| 4.0 | 13.4 | 14.6 | 17.0 | 20.1 | 23.4 | 26.4 | 28.1 |  | 9.2 | 10.2 | 11.9 | 14.0 | 16.1 | 18.1 | 19.3 |
| 5.0 | 15.1 | 16.5 | 19.1 | 22.6 | 26.4 | 29.7 | 31.7 |  | 12.9 | 14.3 | 16.8 | 19.6 | 22.6 | 25.4 | 27.1 |
| 6.0 | 16.7 | 18.2 | 21.2 | 25.0 | 29.2 | 32.9 | 35.1 |  | 15.9 | 17.6 | 20.6 | 24.2 | 27.8 | 31.2 | 33.3 |
| 7.0 | 18.4 | 20.1 | 23.3 | 27.5 | 32.1 | 36.2 | 38.6 |  | 18.0 | 20.0 | 23.5 | 27.5 | 31.6 | 35.5 | 37.9 |
| 8.0 | 20.5 | 22.4 | 26.0 | 30.7 | 35.8 | 40.3 | 43.0 |  | 19.7 | 21.9 | 25.6 | 30.0 | 34.5 | 38.8 | 41.4 |
| 9.0 | 22.9 | 25.0 | 29.0 | 34.3 | 40.0 | 45.1 | 48.1 |  | 20.8 | 23.1 | 27.1 | 31.7 | 36.5 | 41.0 | 43.7 |
| 10.0 | 25.5 | 27.8 | 32.3 | 38.1 | 44.5 | 50.1 | 53.5 |  | 21.8 | 24.2 | 28.4 | 33.2 | 38.3 | 43.0 | 45.8 |
| 11.0 | 28.1 | 30.7 | 35.6 | 42.1 | 49.1 | 55.4 | 59.0 |  | 22.8 | 25.3 | 29.7 | 34.8 | 40.0 | 44.9 | 47.9 |
| 12.0 | 31.0 | 33.9 | 39.3 | 46.5 | 54.2 | 61.1 | 65.2 |  | 23.7 | 26.3 | 30.8 | 36.1 | 41.6 | 46.6 | 49.8 |
| 13.0 | 33.7 | 36.8 | 42.7 | 50.5 | 58.9 | 66.4 | 70.8 |  | 24.7 | 27.5 | 32.2 | 37.7 | 43.4 | 48.7 | 52.0 |
| 14.0 | 35.4 | 38.6 | 44.8 | 53.0 | 61.8 | 69.6 | 74.3 |  | 26.0 | 28.9 | 33.8 | 39.6 | 45.6 | 51.2 | 54.6 |
| 15.0 | 36.2 | 39.5 | 45.8 | 54.1 | 63.2 | 71.2 | 75.9 |  | 27.4 | 30.4 | 35.7 | 41.7 | 48.1 | 54.0 | 57.6 |
| 16.0 | 36.3 | 39.7 | 46.0 | 54.4 | 63.5 | 71.5 | 76.3 |  | 28.8 | 32.0 | 37.5 | 43.9 | 50.6 | 56.8 | 60.6 |
| 17.0 | 36.1 | 39.4 | 45.7 | 54.0 | 63.0 | 71.0 | 75.8 |  | 30.3 | 33.6 | 39.4 | 46.1 | 53.1 | 59.6 | 63.5 |
| 18.0 | 35.6 | 38.9 | 45.1 | 53.3 | 62.2 | 70.1 | 74.8 |  | 31.5 | 35.0 | 41.0 | 48.0 | 55.3 | 62.1 | 66.2 |

RAV_max_ : Maximal right atrial volume

**Supplementary Table S34. Intra-observer and inter-observer reproducibility for the measurement of biventricular size and function with inclusion of trabeculations and papillary muscles in ventricular volume**

|  | Intra-observer reproducibility | | |  | Inter-observer reproducibility | | |
| --- | --- | --- | --- | --- | --- | --- | --- |
|  | Mean difference | Limits of agreement | COV |  | Mean difference | Limits of agreement | COV |
| LVEDV(mL) | 1.7 | -9.3 to 12.6 | 4.3 |  | 1.1 | -10.7 to 13.0 | 4.5 |
| LVESV(mL) | 1.4 | -6.3 to 9.0 | 8.2 |  | 1.1 | -7.6 to 9.8 | 9.0 |
| LVSV(mL) | 0.3 | -7.5 to 8.1 | 4.7 |  | 0.0 | -8.7 to 8.8 | 5.2 |
| LVEF(%) | -0.5 | -6.3 to 5.2 | 3.3 |  | -0.6 | -7.5 to 6.3 | 3.9 |
| LVM(g) | 1.5 | -6.8 to 9.7 | 5.8 |  | 1.5 | -6.8 to 9.7 | 6.1 |
| RVEDV(mL) | 6.6 | -5.2 to 18.3 | 6.2 |  | 5.0 | -10.9 to 20.9 | 6.6 |
| RVESV(mL) | 3.3 | -6.3 to 12.9 | 9.9 |  | 3.4 | -7.7 to 14.5 | 11.0 |
| RVSV(mL) | 3.3 | -7.7 to 14.3 | 4.7 |  | 1.6 | -12.2 to 15.4 | 8.5 |
| RVEF(%) | -0.6 | -9.4 to 8.3 | 5.4 |  | -1.5 | -11.2 to 8.2 | 6.1 |

COV: coefficient of variation; other abbreviations as in Supplementary table S1.

**Supplementary Table S35. Intra-observer and inter-observer reproducibility for the measurement of biventricular size and function with exclusion of trabeculations and papillary muscles in ventricular volume**

|  | Intra-observer reproducibility | | |  | Inter-observer reproducibility | | |
| --- | --- | --- | --- | --- | --- | --- | --- |
|  | Mean difference | Limits of agreement | COV |  | Mean difference | Limits of agreement | COV |
| LVEDV(mL) | 4.9 | -6.5 to 16.4 | 6.3 |  | 3.7 | -6.0 to 13.5 | 5.1 |
| LVESV(mL) | 2.9 | -6.3 to 12.2 | 14.4 |  | 3.0 | -6.4 to 12.4 | 14.7 |
| LVSV(mL) | 2 | -5.6 to 9.6 | 5.2 |  | 0.7 | -6.9 to 8.3 | 4.7 |
| LVEF(%) | -1.9 | -9.3 to 5.6 | 4.3 |  | -2.1 | -10.2 to 6.0 | 4.7 |
| LVM(g) | -1.7 | -13.5 to 10.2 | 6.9 |  | -1.4 | -13.0 to 10.2 | 6.8 |
| RVEDV(mL) | 5.0 | -10.6 to 20.7 | 7.1 |  | 5.4 | -13.3 to 24.1 | 8.2 |
| RVESV(mL) | 2.4 | -7.7 to 12.5 | 11.4 |  | 2.5 | -8.2 to 13.1 | 12.0 |
| RVSV(mL) | 2.7 | -6.7 to 12.0 | 6.6 |  | 2.9 | -11.2 to 17.0 | 9.3 |
| RVEF(%) | -0.1 | -7.1 to 6.9 | 3.9 |  | -0.2 | -9.2 to 8.8 | 5.0 |

COV: coefficient of variation; other abbreviations as in Supplementary table S1.

**Supplementary Table S36. Intra-observer and inter-observer reproducibility for the measurement of atrial volume**

|  | Intra-observer reproducibility | | |  | Inter-observer reproducibility | | |
| --- | --- | --- | --- | --- | --- | --- | --- |
|  | Mean difference | Limits of agreement | COV |  | Mean difference | Limits of agreement | COV |
| iLAV_min_ | -0.4 | -5.3 to 4.4 | 10.7 |  | -0.8 | -7 to 5.4 | 13.6 |
| iLAV_max_ | 0.2 | -11.7 to 12.1 | 9.9 |  | 0.1 | -12.7 to 12.9 | 10.6 |
| iRAV_min_ | 1.8 | -6.1 to 9.6 | 12.1 |  | 1.8 | -6.1 to 9.6 | 13.2 |
| iRAV_max_ | -0.2 | -13.5 to 13.1 | 9.8 |  | -0.4 | -14.1 to 13.3 | 10.1 |

COV: coefficient of variation; iLAV_min_ : Minimal left atrial volume index; iLAV_max_ : Maximal left atrial volume index; iRAV_min_ : Minimal right atrial volume index; iRAV_max_ : Maximal right atrial volume index.
